# COVID-19 and influenza vaccine-hesitancy subgroups

**DOI:** 10.1371/journal.pone.0308159

**Published:** 2024-07-30

**Authors:** Karl O. Mäki, Linda C. Karlsson, Johanna K. Kaakinen, Philipp Schmid, Stephan Lewandowsky, Jan Antfolk, Anna Soveri

**Affiliations:** 1 Department of Psychology and Speech-Language Pathology, University of Turku, Turku, Finland; 2 Department of Clinical Medicine, University of Turku, Turku, Finland; 3 Department of Psychology, Åbo Akademi University, Turku, Finland; 4 INVEST Research Flagship Centre, University of Turku, Turku, Finland; 5 Centre for Language Studies, Radboud University Nijmegen, Nijmegen, The Netherlands; 6 School of Psychological Science, University of Bristol, Bristol, United Kingdom; 7 School of Psychological Science, University of Western Australia, Perth, Australia; 8 Department of Psychology, University of Potsdam, Potsdam, Germany; University of Naples - Parthenope: Universita degli Studi di Napoli Parthenope, ITALY

## Abstract

Health communicators are faced with the challenge that people can hesitate vaccines for different reasons. Our aim was to identify and describe the qualities of distinct COVID-19 and influenza vaccine-hesitancy subgroups to facilitate the development of tailored vaccine-hesitancy communication. In two studies, we used agglomerative hierarchical cluster analysis to identify COVID-19 (N = 554) and influenza (N = 539) vaccine-hesitancy subgroups in the general population based on nine vaccine hesitancy-related variables (intent to get vaccinated, perceived vaccine safety, perceived vaccine efficacy, perceived disease threat, perceived vaccination responsibility, perceived vaccination convenience, distrust in authorities, conspiracy mentality, and reliance on anecdotal testimonies). We identified and described six distinct COVID-19 vaccine-hesitancy subgroups (*the Vaccination Positive*, *the Ambivalent*, *the Fearing Skeptic*, *the Unconvinced*, *the Constrained Skeptic*, and *the Vaccination Opponent*), and three influenza vaccine-hesitancy subgroups (*the Vaccination Positive*, *the Complacent*, and *the Vaccination Opponent*), with different levels of hesitancy. We discuss the implications of the results for health communicators. Our results shed light on the (dis)similarities between people who hesitate COVID-19 and influenza vaccines and suggest that there is greater variety in hesitancy concerning COVID-19 vaccinations than influenza vaccinations. These findings can be used to design and test tailored vaccination messages.

## Introduction

Vaccination is known to be one the safest and most cost-effective methods for reducing the risks of contagious diseases [1]. Vaccines do not merely protect individuals against disease, but they also provide societal and economic benefits by reducing the impact diseases have on healthcare systems [2]. In fact, vaccinations and non-pharmaceutical interventions were the two primary strategies for tackling the COVID-19 pandemic, as both strategies were needed to mitigate the spread of the disease [3]. Yet, despite their benefits, vaccines raise doubts and concerns in some individuals. When not addressed, these concerns can drive people to postpone and even reject important vaccinations [4]. This phenomenon has been labelled vaccine hesitancy [5].

Vaccine hesitancy is a complex phenomenon that varies between individuals, places, and contexts, and it can be summarized as a continuum between full vaccine acceptance and total vaccine refusal [5]. Individuals can hesitate different vaccines, but they can also hesitate the same vaccine for distinct reasons [5, 6]. It is thus important to identify and examine vaccine-hesitancy subgroups, and to do so separately for different vaccines. Identifying idiosyncrasies of subgroups is considered to be a necessary practice in communication campaigns, as it can facilitate efficient allocation of resources and may help communicators design tailored messages that meet specific audience needs and preferences [7]. In fact, research indicates that people perceive tailored messages as more relevant than non-tailored ones [8], and that vaccine messages have a greater impact on vaccine attitudes and vaccination intentions when tailored in accordance with intended audiences [9, 10]. In short, understanding why people hesitate to get vaccinated is key to designing effective vaccination messages and campaigns. Hence, the aims of the present study were to identify and describe COVID-19 and influenza vaccine-hesitancy subgroups in the general population. For this, we used cluster analysis, as clustering algorithms are especially well-suited for finding patterns in population data.

In the next three sections of this article, we first review the literature on vaccine hesitancy and its determinants, then we review the literature on COVID-19 and influenza vaccine-hesitancy subgroups, and then we recapitulate the aims of this article.

### Vaccine-hesitancy determinants

Behavioral change theories, such as the health belief model (HBM) [11] and the theory of planned behavior (TPB) [12], suggest that people can hesitate vaccines for various reasons, ranging from low motivation and negative vaccine attitudes to psychological characteristics such as distrust in authorities. The research evidence for these theories’ role in explaining vaccine hesitancy is substantial [13–16]. In line with the HBM and the TPB, vaccine-hesitancy specific models posit that people hesitate vaccines because of negative vaccine attitudes (the 3C model [5]; the 5C model [17]), and that people can be motivated to hold such attitudes (anti-vaccination attitude-roots [18]). Furthermore, recent research has stressed the importance of and the distinction between individualistic vaccination choices (e.g., based on perceived vaccine safety and the threat of the disease) and altruistic ones (e.g., based on perceived collective responsibility) [19].

In accordance with the above-mentioned models, research has found that people’s attitudes concerning the safety and efficacy of vaccines, the threat of disease, vaccination responsibility, and vaccination convenience are significantly related to people’s level of COVID-19 and influenza vaccine hesitancy [20–22]. More specifically, in a systematic literature review by Kumar and colleagues it was found that people were more hesitant about COVID-19 vaccines when they perceived the safety and efficacy of COVID-19 vaccines to be low, when they perceived the threat of COVID-19 to be low, and when they did not perceive it to be important to get vaccinated to protect others [20]. The results of another systematic literature review showed that people were more hesitant to take COVID-19 vaccines if they perceived vaccination to be inconvenient [21]. Similarly, Schmid and colleagues found in their systematic review that people were more hesitant toward influenza vaccines when they perceived influenza vaccines to be less safe and efficient, when they perceived the threat of influenza to be low, when they did not believe that getting the vaccine would protect others, and when they perceived influenza vaccinations to be inconvenient [22].

Moreover, people with higher trust in authorities are known to be more likely to follow authority recommendations [23], and consequently, to be more likely to accept vaccines [20, 22]. Related to trust, people who are more inclined to believe in conspiracy theories (i.e., have a higher conspiracy mentality) are also less likely to trust authorities [24], to follow authority guidelines [25], and to accept vaccines [20, 26–28]. Conspiratorial claims were also recently identified as a common theme in anti-vaccination arguments, together with an overreliance on anecdotal testimonies [18]. In fact, studies have found that anecdotal vaccine testimonies can bias people’s vaccination decisions [29, 30], and that vaccination opponents often capitalize on this fact by using such testimonies in protests against vaccinations [31, 32].

While studies on the determinants of vaccine hesitancy are important and add to the literature on why people hesitate to take vaccines, such studies provide little knowledge about how these determinants manifest in distinct vaccine-hesitancy profiles within populations. This type of research question necessitates other research strategies, such as the utilization of cluster analysis.

### Vaccine-hesitancy subgroups

A popular approach for gaining insight into vaccine-hesitancy subgroups is the use of clustering algorithms. Several studies have utilized clustering algorithms in the past to investigate COVID-19 and influenza vaccine hesitancy, and the large body of literature on this topic is heterogeneous. Our literature search for studies that have examined COVID-19 and influenza vaccine-hesitancy subgroups revealed 38 articles published before 19.6.2023 (see S4 File for a list of the articles found in the systematic search). The studies have included a wide range of variables in their cluster analyses, ranging from vaccine attitudes [33] and health behaviors [34] to conspiracy beliefs [35] and political orientations [6]. Furthermore, a variety of statistical clustering methods, such as K-means [36], hierarchical cluster analysis [37], and latent profile analysis [38], have been used. There is also great variation in the number of identified subgroups between studies, as the number of subgroups range from two in the study by Falcon and colleagues [39] to 16 in the study by Lindvall and Rönnerstrand [40] (see S4 File for the number of subgroups in each study).

**Fig 1:**
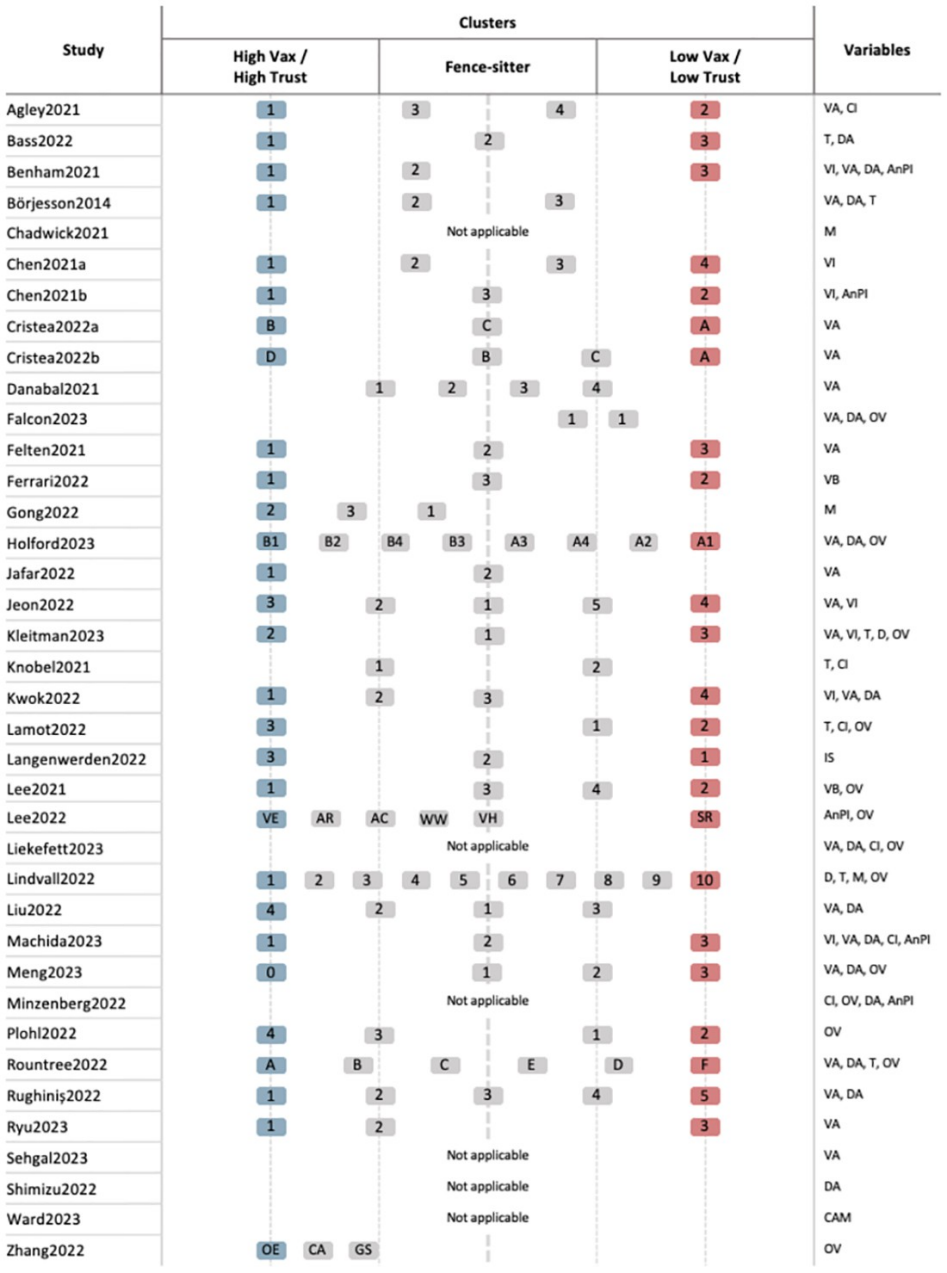
Subgroups identified in previous studies along a scale from high vaccine confidence and trust to low vaccine confidence and trust. High vaccine confidence and trust—(High Vax / High Trust), low vaccine confidence and trust—(Low Vax / Low Trust). The Fence-sitter column encompasses subgroups between the two extremes. The subgroups are labeled for easy cross-referencing with their corresponding studies. Variable abbreviations for variables that were included in the cluster analyses: VA–vaccine attitudes, T–trust in authorities, DA–disease attitudes, VI–vaccination intention, AnPI–adherence to nonpharmaceutical interventions, M–media use, OV–other variables, VB–vaccination behavior, D–demographics, CI–conspiracy ideation, IS–information source, CAM–CAM attitudes.

Due to the many differences between studies, it is difficult to compare and summarize the previously identified vaccine-hesitancy subgroups. Nevertheless, most studies have found that the identified subgroups differ from one another in terms of their degree of vaccine hesitancy (for a graphical representation of the COVID-19 and influenza vaccine-hesitancy subgroups identified in previous studies, see Fig 1). For example, Kwok and colleagues [41] analyzed responses to the 5C scale [17] and found four subgroups that mainly differed from one another regarding how strongly they agreed with the statements on each 5C factor–i.e., confidence, complacency, constraints, calculation, and collective responsibility. In another study, Holford and colleagues [6] performed a cluster analysis on individuals’ endorsement of anti-vaccination arguments that were categorized according to the theoretical framework of anti-vaccination attitude-roots [18]. The cluster analysis resulted in four vaccine-hesitant subgroups that mainly differed in how strongly they endorsed the arguments. In addition, two of the subgroups were characterized by a higher level of social and economic conservatism than the others.

Further contributing to the heterogeneity described above is the fact that some studies include COVID-19- or influenza-related variables in their cluster analyses, whereas others base their clusters on vaccine-general variables and investigate differences in COVID-19- or influenza-related variables between the subgroups. In the following, we focus on studies that construct subgroups based on COVID-19 and influenza variables. There are several studies that have identified subgroups based on attitudes, intentions and behaviors related to COVID-19 vaccines [6, 33, 34, 38, 39, 42–58]. For example, Falcon and colleagues [39] analyzed Spanish, vaccine-hesitant individuals’ reasons for not being vaccinated against COVID-19 and identified two subgroups of individuals: those reluctant to get vaccinated because of health-related issues (e.g., pregnancy or having already been infected with COVID-19) and those expressing distrust in the vaccines and their development, beliefs in conspiracy theories, and low perceived disease risk. In another study, Meng and colleagues [54] conducted cluster analysis on data from 23,397 US adults unvaccinated against COVID-19. They included variables related to the perceived risk of COVID-19, social norms, confidence in the vaccines, and practical issues, and they identified three hesitant subgroups: one slightly hesitant and two very hesitant. The two very hesitant subgroups differed from one another only in terms of mask-wearing behaviors. Despite differences in hesitancy levels, all three subgroups perceived COVID-19 vaccines as accessible.

While several studies have investigated influenza vaccine-hesitancy subgroups in healthcare professionals [59–61], only a few have examined influenza vaccine-hesitancy subgroups specifically in the general population. One such study was carried out in Sweden during the A/H1N1 (swine flu) pandemic in 2009–2010 which focused on the A/H1N1 influenza vaccine [62]. The study identified three subgroups, two of which perceived the risks of A/H1N1 influenza to be low and did not worry much about the disease. Nevertheless, one of these two groups reported a higher trust in authorities than the other. The third group perceived the A/H1N1 influenza risks to be higher and expressed more worry.

Even though the literature on COVID-19 and influenza vaccine-hesitancy subgroups is extensive, no study has, to the best of our knowledge, examined COVID-19 and influenza vaccine-hesitancy subgroups together in a way that makes direct subgroup comparisons possible, nor has any study investigated vaccine-hesitancy subgroups from both a message content and a receptivity to authority communication point of view.

### The current study

The aims of the present study were to utilize agglomerative hierarchical cluster analysis (AHCA) to identify and describe COVID-19 and influenza vaccine-hesitancy subgroups in the general population. As research has found a connection between people’s COVID-19 and influenza vaccination intentions [63], it is possible that the COVID-19 and influenza vaccine-hesitancy subgroups would exhibit similar hesitancy profiles. However, investigating vaccine-hesitancy subgroups separately for COVID-19 and influenza vaccines allowed us to examine both the dissimilarities and the similarities between their respective subgroups. This can provide vaccine-specific information that could be used when designing communication strategies for these vaccines.

In order to make the subgroups informative for the development of vaccine communication, the variables included in the cluster analyses pertained to the information content that can be communicated to the subgroups to decrease vaccine hesitancy and the subgroups’ receptivity to communication from experts and authorities. Based on the previous research findings described above [20–22], we included five variables relating to the information content, namely perceived vaccine safety, perceived vaccine efficacy, perceived disease threat, perceived vaccination responsibility, and perceived vaccination convenience. Additionally, we included three variables relating to the receptivity to communication from experts and authorities. These were trust in health authorities, conspiracy mentality, and reliance on anecdotal testimonies.

Finally, we included one variable representing intention to get vaccinated to be able to identify the subgroups that are the most and the least likely to take COVID-19 and influenza vaccines, as it is important from the perspective of communication resource allocation.

In sum, our aims were to:

1. Identify COVID-19 and influenza vaccine-hesitancy subgroups relevant to vaccine communication.
2. Describe the qualities of the identified subgroups to facilitate the development of tailored vaccine-hesitancy communication.

## Methods

### COVID-19 and influenza vaccinations in Finland

In Finland, where the present study was conducted, influenza vaccines are only recommended to people belonging to specific risk groups and are compulsory for healthcare professionals working with patients specifically vulnerable to influenza [64]. In contrast, COVID-19 vaccines are recommended to all Finnish adults, as well as to children aged 12 and above who belong to certain risk groups [65]. While the Finnish Institute for Health and Welfare has recommended three COVID-19 vaccine doses for all adults [65], only 64.9% of Finnish adults have received all three doses as of 1.1.2024 [66].

### Participants and procedure

Ethical permission for this study was given by the Research Ethics Committee in Psychology and Logopedics of Åbo Akademi University. Data were collected between 8.10.-1.11.2021 with an electronic survey that was advertised to Finnish adults on Facebook, Messenger, and Instagram. When entering the survey, all participants first gave their written informed consent by ticking a checkbox. Participants were then asked to fill out a survey measuring perceived safety of vaccines in general, trust in health authorities, conspiracy mentality, reliance on anecdotal testimonies, as well as COVID-19 and influenza vaccination status. After this, participants randomly received a question either about how likely they were to take a seasonal COVID-19 vaccine or the next seasonal influenza vaccine. Those who responded 80% or more were given the corresponding question about the other vaccine. As our goal was to identify subgroups of people that are vaccine hesitant, we excluded participants that reported being 80% or more likely to take both vaccines. Those who reported being less than 80% likely to take one of the vaccines continued to answer questions regarding the same vaccine. These questions concerned vaccine safety, vaccine efficacy, disease threat, vaccination responsibility, vaccination convenience, and vaccination intention (see the Materials section for more information on the measures). Lastly, the participants took part in an online intervention and filled out postintervention surveys. The results from this experiment have been reported elsewhere and are not in the focus of this study [67]. In the current study, we analyzed the preintervention measures from the dataset.

The total sample consisted of 1,942 participants, of whom 843 were excluded based on them responding 80% or more on the vaccination intention questions. Additionally, six participants were excluded due to missing values on the items used for clustering. The final sample thus consisted of 1,093 vaccine-hesitant participants, of whom 554 answered COVID-19 vaccination questions and 539 answered influenza vaccination questions.

### Materials

#### Vaccination intention

Vaccination intention questions were answered on a visual slider with the anchors “Very unlikely” and “Very likely”. Participants’ answers on the slider were automatically coded on a scale from 0 (Very unlikely) to 100 (Very likely). In the COVID-19 sample, participants’ *intention to take a seasonal COVID-19 vaccine* was measured with the question “How likely would you be to take a seasonal COVID-19 vaccine, should one become available?”. Their *intention to take a third COVID-19 vaccine dose* was similarly measured with the question “How likely would you be to take a third COVID-19 vaccine, should one become available?”. In the influenza sample, participants’ *intention to take the next influenza vaccine* was measured with the question “How likely are you to take the next seasonal influenza vaccine?”.

#### Vaccine and disease attitudes

Vaccine and disease attitudes were measured with a single item per construct. All questions except one (described below) were answered on a visual slider. Participants’ answers were automatically coded on a scale ranging from 0 to 100. *Perceived vaccine safety* was measured for COVID-19 vaccines, influenza vaccines, and for vaccinations in general. Perceived COVID-19 and influenza vaccine safety were measured with the question “How safe do you think that the [vaccine] is/are?” (anchors: Not safe at all–Very safe). We measured the perceived safety of vaccines in general with the vaccine-confidence item from the 5C scale “I am completely confident that vaccines are safe” [17], which was answered on a seven-point Likert scale (anchors: Strongly disagree–Strongly agree). *Perceived vaccine efficacy* was measured with the question “How efficient do you think that the [vaccine] is/are?” (anchors: Not efficient at all–Very efficient). *Perceived disease threat* was measured with the question “How big of a threat is [disease] to your health?” (anchors: Not at all threatening–Very threatening). *Perceived vaccination responsibility* was measured with the statement “It is important to take the [vaccine] as it also protects others.” (anchors: Strongly disagree–Strongly agree). *Perceived vaccination convenience* was measured with the question “How easy is it to get the [vaccine] in Finland.” (anchors: Not at all easy–Very easy).

#### Trust in health authorities

*Trust in health authorities* was measured with four statements (e.g., “I trust the information I receive from health authorities about vaccines.”) on a Likert scale ranging from 1 (Strongly disagree) to 7 (Strongly agree). Two of the statements were originally created by Karlsson et al. [68]. We created the remaining two statements for the present study to be able to analyze the fit of trust in health authorities as a latent factor.

#### Conspiracy mentality

*Conspiracy mentality* was measured with the five-item Conspiracy Mentality Questionnaire [69]. The questionnaire consisted of statements, such as “I think that many very important things happen in the world, which the public is never informed about.”, that were answered on an eleven-point fully labeled scale from 0% (Certainly not) to 100% (Certain).

#### Reliance on anecdotal testimonies

*Reliance on anecdotal testimonies* was measured with the six-item format preference scale [67]. The scale consisted of statements, such as “When faced with statistical data that contradicts people’s experiences, I prefer to trust people’s reported experiences.”, that were answered on a seven-point Likert scale ranging from 1 (Strongly disagree) to 7 (Strongly agree). Lower scores indicate more reliance on statistical information and higher scores indicate more reliance on anecdotal testimonies.

### Statistical analyses

The COVID-19 and the influenza samples were analyzed separately, but the same statistical procedures were used in both instances. We conducted all analyses in *R* version 4.3.0 [70].

#### Confirmatory factor analysis of scales

Confirmatory factor analysis (CFA) is a statistical method used for estimating latent factors that cannot be directly measured [71]. CFA takes measurement error into account, which generally results in cleaner estimates of the construct of interest in the form of factor scores [71]. We conducted separate CFAs for constructs with multiple items to investigate the fit of the factors and to extract their respective factor scores for use in later analyses. These constructs were trust in health authorities (four indicators), conspiracy mentality (five indicators; predefined error correlation between the first two items), and reliance on anecdotal testimonies (six indicators; predefined error correlation between reverse-scored items). For more details about the CFA analyses, see Mäki and colleagues [67]. We used the lavaan package [72] for the CFAs with robust WLS estimation.

#### Cluster analysis

We used AHCA to identify COVID-19 and influenza vaccine-hesitancy subgroups. Compared to variable-centered methods such as linear regression and factor analysis, AHCA is an explorative observation-centered analysis that groups observations based on a similarity metric and a linkage method [73]. In AHCA, each observation (in this case, an individual) is treated as its own cluster. The AHCA iteratively merges the two most similar clusters based on a chosen similarity metric and linkage method until all observations form a single cluster. This also results in a visual representation of the subgroups in the form of a dendrogram (for an example, see the S1 Fig in S1 File). Lastly, the optimal number of clusters is commonly chosen based on a stopping rule [73]. In this study, we chose to use the AHCA as it does not make assumptions regarding the distribution of the data nor about the number of clusters, and because it produces the forementioned visual representation of the subgroups that is easy to understand.

First, we derived the factor scores for trust in health authorities, conspiracy mentality, and reliance on anecdotal testimonies from the CFAs described above. Second, we z-transformed the measures vaccination intention, perceived vaccine safety (vaccine-specific and vaccine-general items separately), perceived vaccine efficacy, perceived disease threat, perceived vaccination responsibility, perceived vaccination convenience, trust in health authorities, conspiracy mentality, and reliance on anecdotal testimonies, so that all variables were on the same scale for clustering. Third, we calculated the distance matrix between participants with Euclidean distances and conducted the AHCAs using Ward’s method [74]. Unlike other linkage methods, Ward’s method considers the increase in within-cluster variance when merging clusters, with the aim of creating compact clusters. Lastly, we determined the optimal number of clusters using the Duda-Hart stopping rule [73], which considers the ratio of between- and within-cluster variances.

## Results

### Measurement models and internal consistency of scales

S1 Table in S1 File shows the fit indices for the CFA models of both samples, and S2 and S3 Tables in S1 File show the respective models’ factor loadings. In both samples, the conspiracy mentality and the reliance on anecdotal testimonies scales’ fit indices showed excellent fit, except for the RMSEA. All fit indices for the trust in health authorities scale showed excellent fit, except for the RMSEA in the influenza sample. In both samples, all standardized factor loadings were above 0.60, except for the one item on the reliance on anecdotal testimonies scale, which was over 0.40 in both samples. As the majority of the indices suggested excellent fit for all factor models, we chose to retain the specified factors and use factor scores in subsequent analyses.

### COVID-19 vaccine-hesitancy subgroups

**Fig 2:**
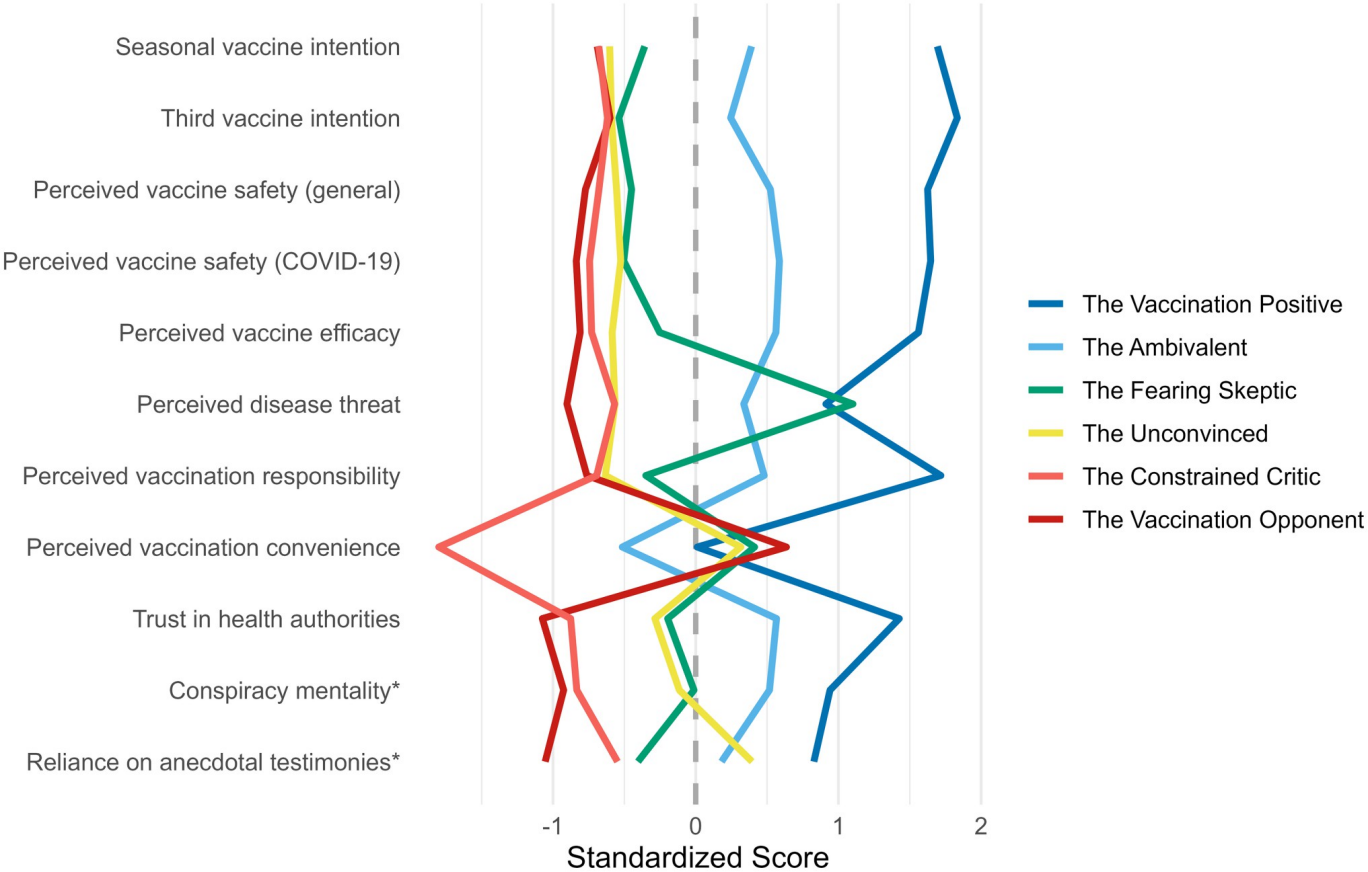
Parallel coordinate plot with mean scores for the COVID-19 vaccine-hesitancy subgroups. Higher values indicate more pro-vaccination attitudes, and lower values indicate more anti-vaccination attitudes. The * sign indicates that the variable has been reverse coded so that higher conspiracy mentality scores equal less conspiracy mentality and higher reliance on anecdotal testimonies scores equal less reliance on anecdotal testimonies.

We identified six COVID-19 vaccine-hesitancy subgroups based on the Duda-Hart stopping rule (see S4 Table in S1 File). For a visual overview of z-transformed mean scores of the hesitancy subgroups in relation to each other, see Fig 2.

We named the first COVID-19 vaccine-hesitancy subgroup *the Vaccination Positive*^*Cov*^ (98 participants, 17.7% of the COVID-19 sample), as the participants of this subgroup held mostly positive vaccine attitudes and had, by a large margin, the highest overall intention to take a third and a seasonal COVID-19 vaccine (see Tables 1 and 2 for z-transformed variable means and demographic variable counts). They scored the highest on the perceived vaccination responsibility, perceived vaccine safety (general & COVID-19), perceived vaccine efficacy, and trust in health authorities measures, and among the highest on the measure of perceived vaccination convenience. Their lowest vaccine attitude score, albeit higher than in most other subgroups, was for perceived threat of COVID-19. Furthermore, participants of this subgroup most strongly preferred statistical information over anecdotal information and scored the lowest on conspiracy mentality. It is worth to restate that we excluded participants from the analyses who responded that they were 80–100% likely to take a seasonal COVID-19 vaccine, and that despite this, 98 participants were still characterized by positive attitudes toward the COVID-19 vaccines.

**Table 1:** Means and standard deviations for z-transformed variables for the COVID-19 vaccine-hesitancy subgroups.

Variable	The Vaccination Positive		The Ambivalent		The Fearing Skeptic		The Unconvinced		The Constrained Critic		The Vaccination Opponent	
	*M*_*z*_	*SD*	*M*_*z*_	*SD*	*M*_*z*_	*SD*	*M*_*z*_	*SD*	*M*_*z*_	*SD*	*M*_*z*_	*SD*
Seasonal vaccine intention	1.69	0.51	0.39	0.81	-0.36	0.62	-0.60	0.24	-0.68	0.07	-0.69	0.09
Third vaccine intention	**1.83**	0.53	0.24	0.76	-0.54	0.17	-0.59	0.12	-0.62	0.02	-0.60	0.21
Perceived vaccine safety (general)	1.62	0.56	0.52	0.75	-0.45	0.53	-0.55	0.36	-0.68	0.23	-0.77	0.00
Perceived vaccine safety (COVID-19)	1.65	0.40	**0.59**	0.59	-0.50	0.40	-0.52	0.43	-0.74	0.48	-0.84	0.17
Perceived vaccine efficacy	1.56	0.42	0.56	0.67	-0.25	0.74	-0.59	0.42	-0.73	0.51	-0.81	0.19
Perceived disease threat	0.91	0.90	0.34	0.82	**1.10**	0.85	-0.57	0.41	-0.57	0.65	-0.90	0.19
Perceived vaccination responsibility	1.72	0.28	0.48	0.72	-0.35	0.58	**-0.63**	0.26	-0.69	0.24	-0.76	0.08
Perceived vaccination convenience	0.01	0.73	-0.51	1.38	0.41	0.45	0.32	0.52	**-1.80**	0.44	0.64	0.10
Trust in health authorities	1.43	0.57	0.57	0.50	-0.20	0.63	-0.29	0.52	-0.88	0.53	**-1.07**	0.47
Conspiracy mentality	-0.94	0.72	-0.52	0.75	0.01	0.98	0.12	0.64	0.83	0.70	0.93	0.84
Reliance on anecdotal testimonies	-0.83	0.72	-0.18	0.79	0.41	0.79	-0.40	0.70	0.55	0.93	1.05	0.80

Bolded values indicate the defining variable for each subgroup. For the resulting dendrogram (S1 Fig in S1 File), as well as unstandardized variable means and standard deviations for the COVID-19 vaccine-hesitancy subgroups (S5 Table), please refer to the S1 File. For analyses and a visual overview of demographic differences between the COVID-19 vaccine-hesitancy subgroups (S2 Fig), see the S1 File.

**Table 2:** Demographic information for the COVID-19 vaccine-hesitancy subgroups.

Variable	The Vaccination Positive		The Ambivalent		The Fearing Skeptic		The Unconvinced		The Constrained Critic		The Vaccination Opponent		Sample	
	*n*	%	*n*	%	*n*	%	*n*	%	*n*	%	*n*	%	*n*	%
Age														
18–29	13	13.3%	10	9.4%	4	6.2%	13	9.2%	5	11.1%	4	4.0%	49	8.8%
30–39	13	13.3%	20	18.9%	8	12.5%	32	22.7%	10	22.2%	20	20.0%	103	18.6%
40–49	29	29.6%	19	17.9%	13	20.3%	34	24.1%	8	17.8%	31	31.0%	134	24.2%
50–59	22	22.4%	22	20.8%	14	21.9%	20	14.2%	7	15.6%	21	21.0%	106	19.1%
60–69	11	11.2%	12	11.3%	8	12.5%	8	5.7%	4	8.9%	4	4.0%	47	8.5%
70–79	3	3.1%	4	3.8%	5	7.8%	5	3.5%	1	2.2%	1	1.0%	19	3.4%
80–89	-	-	1	0.9%	-	-	-	-	-	-	-	-	1	0.2%
Missing	7	7.1%	18	17.0%	12	18.8%	29	20.6%	10	22.2%	19	19.0%	95	17.1%
Gender														
Man	13	13.3%	22	20.8%	3	4.7%	26	18.4%	13	28.9%	22	22.0%	99	17.9%
Woman	82	83.7%	80	75.5%	55	85.9%	105	74.5%	23	51.1%	74	74.0%	419	75.6%
Other	2	2.0%	-	-	2	3.1%	-	-	1	2.2%	1	1.0%	6	1.1%
Do not wish to say	1	1.0%	4	3.8%	3	4.7%	9	6.4%	8	17.8%	2	2.0%	27	4.9%
Missing	-	-	-	-	1	1.6%	1	0.7%	-	-	1	1.0%	3	0.5%
Education														
Higher	59	60.2%	56	52.8%	33	51.6%	94	66.7%	18	40.0%	53	53.0%	313	56.5%
Lower	37	37.8%	46	43.4%	30	46.9%	43	30.5%	25	55.6%	41	41.0%	222	40.1%
Other	2	2.0%	4	3.8%	1	1.6%	4	2.8%	2	4.4%	4	4.0%	17	3.1%
Missing	-	-	-	-	-	-	-	-	-	-	2	2.0%	2	0.4%
Live														
Uusimaa	41	41.8%	28	26.4%	18	28.1%	51	36.2%	11	24.4%	31	31.0%	180	32.5%
Southwest Finland	13	13.3%	22	20.8%	11	17.2%	13	9.2%	4	8.9%	8	8.0%	71	12.8%
Other	44	44.9%	55	51.9%	35	54.7%	77	54.6%	29	64.4%	58	58.0%	298	53.8%
Missing	-	-	1	0.9%	-	-	-	-	1	2.2%	3	3.0%	5	0.9%
Healthcare experience														
Yes	35	35.7%	37	34.9%	21	32.8%	59	41.8%	21	46.7%	39	39.0%	212	38.3%
No	63	64.3%	67	63.2%	43	67.2%	82	58.2%	23	51.1%	61	61.0%	339	61.2%
Missing	-	-	2	1.9%	-	-	-	-	1	2.2%	-	-	3	0.5%
Had COVID-19														
Yes	4	4.1%	12	11.3%	2	3.1%	16	11.3%	10	22.2%	10	10.0%	54	9.7%
No	94	95.9%	94	88.7%	62	96.9%	125	88.7%	32	71.1%	89	89.0%	496	89.5%
Missing	-	-	-	-	-	-	-	-	3	6.7%	1	1.0%	4	0.7%
Have taken a COVID-19 vaccine														
Yes	96	98.0%	48	45.3%	9	14.1%	9	6.4%	1	2.2%	4	4.0%	167	30.1%
No	2	2.0%	58	54.7%	55	85.9%	132	93.6%	44	97.8%	96	96.0%	387	69.9%
Missing	-	-	-	-	-	-	-	-	-	-	-	-	-	-
N	98	100%	106	100%	64	100%	141	100%	45	100%	100	100%	554	100%

We named the second COVID-19 vaccine-hesitancy subgroup *the Ambivalent*^*Cov*^ (106 participants, 19.1% of the COVID-19 sample) as they were neither strongly for nor against vaccines. This subgroup was the second most vaccine-positive group after *the Vaccination Positive*^*Cov*^, with scores just below the middle of the unstandardized scales on all other measures except for perceived vaccination convenience, where the score was in the upper part of the unstandardized scale, indicating that also this subgroup considered vaccines easily accessible in Finland. The participants of *the Ambivalent*^*Cov*^ hesitancy subgroup showed only a slight preference for statistical information and scored low on conspiracy mentality.

We named the third hesitancy subgroup *the Fearing Skeptic*^*Cov*^ (64 participants, 11.6% of the COVID-19 sample), since these participants perceived the threat of COVID-19 to be the highest of all subgroups, but at the same time they had almost no intentions to take a third or a seasonal COVID-19 vaccine and had low unstandardized scores on perceived vaccine safety, perceived vaccination responsibility, perceived vaccine efficacy, and trust in health authorities measures. They did, however, score in the upper part of the unstandardized scale when it comes to the perceived vaccination convenience measures. Furthermore, the participants of this hesitancy subgroup relied just slightly more on anecdotal testimonies than on statistics and scored slightly above the middle on the unstandardized conspiracy mentality scale.

We named the fourth hesitancy subgroup *the Unconvinced*^*Cov*^, as these participants held largely negative vaccine attitudes without having a high distrust in health authorities, a high conspiracy mentality, nor a high reliance on anecdotal testimonies. This was the most common COVID-19 hesitancy subgroup (141 participants, 25.5% of the COVID-19 sample). In this subgroup, the likelihood of getting vaccinated was close to zero and the attitudes to vaccines were in general very negative. The participants in this subgroup also considered the threat of COVID-19 to be very low, which clearly separates *the Unconvinced*^*Cov*^ from *the Fearing Skeptic*^*Cov*^. Like most other subgroups, *the Unconvinced*^*Cov*^ perceived the convenience of vaccinations to be very high. They also scored slightly above the middle on the unstandardized conspiracy mentality scale and showed a slight preference for statistics over anecdotes.

We named the fifth hesitancy subgroup *the Constrained Critic*^*Cov*^, since their vaccine attitudes were very negative, and they considered the COVID-19 vaccinations to be far more inconvenient than other subgroups. This was the rarest hesitancy subgroup (47 participants, 8.5% of the COVID-19 sample). Participants of this subgroup had no intention to get vaccinated and scored very low on perceived vaccination responsibility, perceived vaccine safety, perceived vaccine efficacy, perceived threat of COVID-19, and trust in health authorities, and very high on conspiracy mentality. Participants of this hesitancy subgroup also relied more on anecdotal testimonies than on statistics.

We named the sixth and last hesitancy subgroup *the Vaccination Opponent*^*Cov*^ (100 participants, 18.1% of the COVID-19 sample), as participants of this subgroup held very negative vaccine attitudes, most heavily relied on anecdotal testimonies, scored the highest on conspiracy mentality, and reported very low trust in health authorities. Compared to *the Constrained Critic*^*Cov*^, *the Vaccination Opponent*^*Cov*^ subgroup had similarly low intentions to get vaccinated and had even lower scores on perceived vaccination responsibility, perceived threat of COVID-19, perceived vaccine efficacy, and perceived vaccine safety. Lastly, this hesitancy subgroup rated the convenience of COVID-19 vaccinations to be the highest.

### Influenza vaccine-hesitancy subgroups

**Fig 3:**
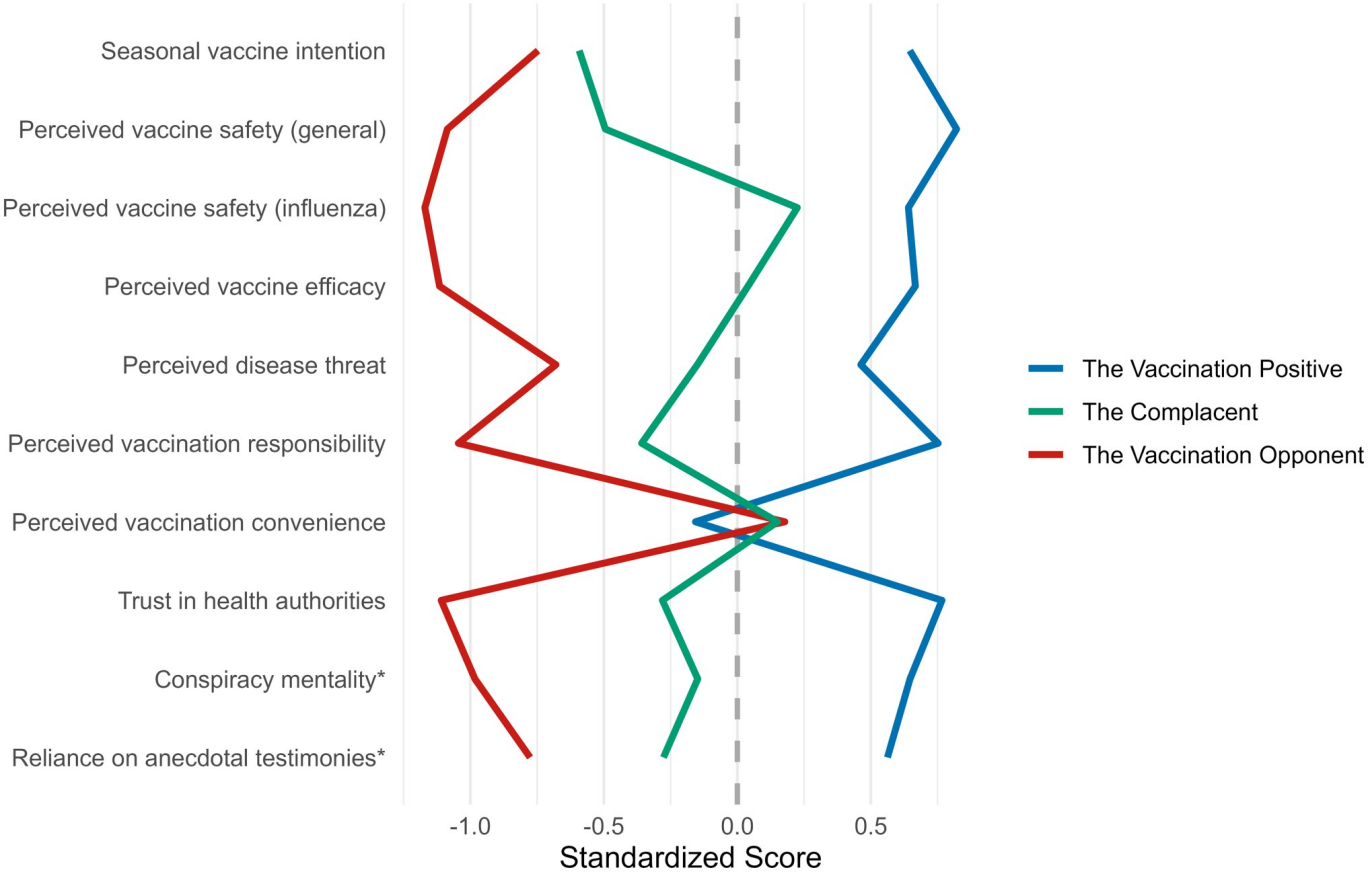
Parallel coordinate plot with mean scores for the influenza vaccine-hesitancy subgroups. Higher values indicate more pro-vaccination attitudes, and lower values indicate more anti-vaccination attitudes. The * sign indicates that the variable has been reverse coded so that higher conspiracy mentality scores equal less conspiracy mentality and higher reliance on anecdotal testimonies scores equal less reliance on anecdotal testimonies.

We identified three influenza vaccine-hesitancy subgroups based on the Duda-Hart stopping rule (see S6 Table in S1 File). For a visual overview of the mean scores for the hesitancy subgroups in relation to each other, see Fig 3.

We named the first influenza hesitancy subgroup *the Vaccination Positive*^*Inf*^ despite them having low intentions to get the next influenza vaccine, because participants in this subgroup held otherwise positive vaccine attitudes. It was the most common influenza vaccine-hesitancy subgroup by a large margin (279 participants, 51.8% of the influenza sample). Participants of this hesitancy subgroup scored the highest on the intention to take the next influenza vaccine, perceived vaccination responsibility, perceived threat of influenza, perceived vaccine safety (general & influenza), perceived vaccine efficacy, and trust in health authorities (see Tables 3 and 4 for z-transformed variable means and demographic variable counts). Moreover, they most strongly preferred statistical information and scored the lowest on conspiracy mentality. They did, however, also score the lowest on perceived vaccination convenience.

**Table 3:** Means and standard deviations for z-transformed variables for the influenza vaccine-hesitancy subgroups.

Variable	The Vaccination Positive		The Complacent		The Vaccination Opponent	
	*M*_*z*_	*SD*	*M*_*z*_	*SD*	*M*_*z*_	*SD*
Seasonal vaccine intention	0.65	1.01	**-0.59**	0.32	-0.75	0.10
Perceived vaccine safety (general)	**0.82**	0.53	-0.49	0.64	-1.09	0.28
Perceived vaccine safety (influenza)	0.64	0.49	0.22	0.58	**-1.17**	0.72
Perceived vaccine efficacy	0.67	0.64	0.04	0.74	-1.11	0.48
Perceived disease threat	0.46	0.96	-0.15	0.90	-0.68	0.63
Perceived vaccination responsibility	0.75	0.70	-0.36	0.64	-1.04	0.25
Perceived vaccination convenience	-0.16	1.02	0.15	0.82	0.18	1.01
Trust in health authorities	0.77	0.58	-0.28	0.48	-1.11	0.50
Conspiracy mentality	-0.65	0.71	0.15	0.50	0.98	0.75
Reliance on anecdotal testimonies	-0.56	0.80	0.28	0.77	0.78	0.81

Bolded values indicate the defining variable for each subgroup. For the resulting dendrogram (S3 Fig in S1 File), and unstandardized variable means and standard deviations for the influenza vaccine-hesitancy subgroups (S7 Table), please refer to the S1 File. For analyses and a visual overview of demographic differences between the influenza vaccine-hesitancy subgroups (S4 Fig), see the S1 File.

**Table 4:** Demographic information for the influenza vaccine-hesitancy subgroups.

Variable	The Vaccination Positive		The Complacent		The Vaccination Opponent		Sample	
	*n*	%	*n*	%	*n*	%	*n*	%
Age								
18–29	33	11.8%	17	18.9%	19	11.2%	69	12.8%
30–39	61	21.9%	25	27.8%	40	23.5%	126	23.4%
40–49	59	21.1%	18	20.0%	38	22.4%	115	21.3%
50–59	39	14.0%	9	10.0%	25	14.7%	73	13.5%
60–69	48	17.2%	8	8.9%	17	10.0%	73	13.5%
70–79	12	4.3%	2	2.2%	2	1.2%	16	3.0%
80–89	1	0.4%	-	-	-	-	1	0.2%
Missing	26	9.3%	11	12.2%	29	17.1%	66	12.2%
Gender								
Man	55	19.7%	12	13.3%	51	30.0%	118	21.9%
Woman	214	76.7%	72	80.0%	105	61.8%	391	72.5%
Other	5	1.8%	1	1.1%	1	0.6%	7	1.3%
Do not wish to say	3	1.1%	5	5.6%	11	6.5%	19	3.5%
Missing	2	0.7%	-	-	2	1.2%	4	0.7%
Education								
Higher	183	65.6%	51	56.7%	86	50.6%	320	59.4%
Lower	92	33.0%	36	40.0%	76	44.7%	204	37.8%
Other	4	1.4%	3	3.3%	7	4.1%	14	2.6%
Missing	-	-	-	-	1	0.6%	1	0.2%
Live								
Uusimaa	104	37.3%	29	32.2%	51	30.0%	184	34.1%
Southwest Finland	44	15.8%	20	22.2%	14	8.2%	78	14.5%
Other	131	47.0%	41	45.6%	101	59.4%	273	50.6%
Missing	-	-	-	-	4	2.4%	4	0.7%
Healthcare experience								
Yes	49	17.6%	19	21.1%	45	26.5%	113	21.0%
No	229	82.1%	71	78.9%	124	72.9%	424	78.7%
Missing	1	0.4%	-	-	1	0.6%	2	0.4%
Have taken the last season’s influenza vaccine								
Yes	43	15.4%	4	4.4%	3	1.8%	50	9.3%
No	235	84.2%	86	95.6%	166	97.6%	487	90.4%
Missing	1	0.4%	-	-	1	0.6%	2	0.4%
N	279	100%	90	100%	170	100%	539	100%

We named the second hesitancy subgroup *the Complacent*^*Inf*^, since their vaccination intentions were very low while their attitudes to vaccines were mixed. This was the rarest hesitancy subgroup (90 participants, 16.7% of the influenza sample). Participants in this subgroup reported a very low intention to take the next influenza vaccine. They scored quite low on perceived vaccination responsibility and considered the threat of influenza to be small. At the same time, they considered the vaccines moderately safe (general & influenza) and moderately efficient, and they reported moderate trust in health authorities. Like *the Vaccination Positive*^*Inf*^, they demonstrated somewhat high levels of perceived vaccination convenience. *The Complacent*^*Inf*^ subgroup scored low on conspiracy mentality and showed a slight preference for statistics over anecdotes.

We named the third and last hesitancy subgroup *the Vaccination Opponent*^*Inf*^ (170 participants, 31.5% of the influenza sample), as participants of this subgroup had no intentions to take the influenza vaccine, scored very low on most vaccine attitude measures, reported very low trust in health authorities, scored high on conspiracy mentality, and reported a slight preference for anecdotal information. However, participants of this hesitancy subgroup scored the highest on the perceived vaccination convenience measure.

## Discussion

The aims of this study were to identify and describe COVID-19 and influenza vaccine-hesitancy subgroups to guide the development of tailored vaccine-hesitancy communication. We based our cluster analyses on nine known vaccine-hesitancy determinants that give information on the information content that can be communicated to the subgroups to decrease vaccine hesitancy, and information on how receptive the subgroups are to authority/expert-based health communication.

Using hierarchical cluster analysis, we identified three influenza and six COVID-19 vaccine-hesitancy subgroups. The number of influenza vaccine-hesitancy subgroups was in line with previous research investigating influenza vaccine-hesitancy subgroups in the general population [62]. When it comes to COVID-19 vaccine-hesitancy subgroups, there is more variation in previous studies (see S4 File).

Comparing the present results to those of previous research is not straightforward due to substantial variation between studies in the methods employed. Nevertheless, the common pattern that individuals tend to cluster according to their level of vaccine hesitancy (e.g., [6, 62]), was visible also in the present study, as the main difference between the subgroups laid in the degree to which the individuals held negative attitudes to vaccines, such as worries about the safety and efficacy of the vaccine, and the degree to which they demonstrated other anti-vaccination characteristics, such as distrust in health authorities, reliance on anecdotal testimonies or conspiracy mentality. This was particularly clear when it came to the three influenza vaccine-hesitancy subgroups. Among the COVID-19 vaccine-hesitancy subgroups, the two most vaccine positive subgroups (t*he Vaccination Positive* ^*Cov*^ and *the Ambivalent* ^*Cov*^) followed this same pattern. However, the remaining four subgroups (*the Fearing Sceptic* ^*Cov*^, *the Unconvinced* ^*Cov*^, *the Constrained Critic* ^*Cov*^, *the Vaccination Opponent* ^*Cov*^) were very similar to each other when it came to their level of hesitancy, as all four had low/no intention to get vaccinated, considered the COVID-19 vaccine as unsafe and ineffective, and felt that it was not important to get vaccinated to protect others. The difference between these subgroups instead showed up in the degree to which the individuals distrusted health authorities, believed in conspiracy theories and relied on anecdotal information. Although these qualities were present in all four subgroups, they were particularly strong in two of them (*the Constrained Critic* ^*Cov*^ *and the Vaccination Opponent* ^*Cov*^). This was a clear difference between the COVID-19 and the influenza vaccine-hesitancy subgroups, as there was only one influenza vaccine-hesitancy subgroup that strongly opposed vaccination (*the Vaccination Opponent* ^*Inf*^). The individuals in *the Vaccination Opponent* ^*Inf*^ subgroup corresponded to *the Constrained Critic* ^*Cov*^ and *the Vaccination Opponent* ^*Cov*^, in that they too had no trust in health authorities, very high tendencies to believe in conspiracy theories and to rely on anecdotal information. The reason for why the analyses identified four subgroups that were strongly against COVID-19 vaccination, but only one that was strongly against influenza vaccination, may be that people have formed more detailed opinions on the COVID-19 vaccines due to the enormous amount of information–not to mention misinformation–that has been available about the pandemic and the vaccine. Additionally, people were essentially forced to form an opinion on COVID-19 vaccinations due to the wide-scale societal impact of COVID-19 and the extensive and prolonged public discourse surrounding the topic. In contrast, influenza vaccinations are not universally recommended for laypeople, have not gained similar media attention, and thus, people who do not belong to known risk groups might not even have considered influenza vaccinations prior to this study.

Even though people who strongly oppose vaccination generally tend to reject expert/authority-based communication, it is possible that the *Constrained Critic* ^*Cov*^ *and Vaccination Opponent* ^*Cov*, *Inf*^ do it even more, as distrust, conspiracy mentality, and reliance of anecdotal testimonies are aspects that are known to increase the risk of science rejection [18, 75, 76]. For all five subgroups with strong anti-vaccination attitudes, it may be necessary to use communication techniques, such as Motivational Interviewing [77], to create a common ground and try to increase the individual’s motivation to change their attitudes before any information is provided. Communicating with people who believe in conspiracy theories, in addition, needs to be done with caution, in order to prevent entrenchment of beliefs and to avoid escalation of mistrust [76, 78].

Even if the remaining subgroups (*the Vaccination Positive* ^*Cov*, *Inf*^, *the Complacent*^*Inf*^, and *the Ambivalent*^*Cov*^) vary in their degree of vaccine hesitancy, they all doubt vaccination at least to some degree and their level of distrust, conspiracy mentality, and reliance on anecdotal testimonies are neither very low nor very high. These so-called fence-sitters are important from a health communication perspective, as they are believed to be more likely to change their views on vaccines than individuals who have strongly negative attitudes towards vaccines [79, 80]. For all these subgroups–but in particular for *the Complacent*^*Inf*^, and *the Ambivalent*^*Cov*^–it may be important to give information on the safety and efficacy of vaccines, the threat of the disease, and to correct misinformation [10, 81]. These subgroups could also benefit from prosocial vaccination messages that emphasize the importance of getting vaccinated to protect others. Previous studies have found that people are more willing to take vaccines when they think that doing so protects others [20, 22], and that vaccine messaging that promotes vaccinating to protect others can in fact increase vaccine uptake [82]. Finally, it is possible that they would benefit from making vaccinations more convenient, as removing vaccination barriers is known to be an effective method for increasing vaccination rates for people that already hold positive vaccine attitudes [4].

Interestingly, perceived vaccination convenience did not seem to follow the same gradual pattern as the other variables, as most of the five strongly vaccine negative groups considered vaccination to be more convenient than the four subgroups with less negative attitudes. This stands in contrast to previous studies that have found that less vaccine-hesitant subgroups tend to perceive the convenience of vaccines higher than more vaccine-hesitant subgroups [41, 54, 55]. It is possible that this discrepancy is explained by differences in how convenience has been operationalized. The previous studies have measured vaccination convenience in terms of vaccination barriers whereas we used a subjective estimate of how easy it would be for participants to get vaccinated.

A strength in the present study is that we employed the same set of variables in both cluster analyses to enable comparisons between influenza and COVID-19 vaccine-hesitancy subgroups. Next to the discrepancy in number of subgroups, the most obvious difference between the influenza and the COVID-19 vaccine-hesitancy subgroups was that the influenza vaccine-hesitancy subgroups were clearly less willing to get vaccinated. For example, the influenza vaccine-hesitancy subgroup with the most positive attitudes to influenza vaccines on average indicated that their intention to get vaccinated was M = 38.60 (SD = 27.55), while the corresponding likelihood for the most vaccine-positive COVID-19 vaccine-hesitancy subgroup was M = 67.40 (SD = 14.36). This difference is surprising as the two subgroups were very similar when it comes to the individuals’ perceptions of vaccination convenience, vaccine safety and efficacy, level of trust in health authorities, conspiracy mentality and reliance on anecdotal testimonies. However, the perceived threat of the disease and the perceived responsibility to get vaccinated to protect others was clearly lower in the influenza vaccine-hesitancy subgroup than in the COVID-19 vaccine-hesitancy subgroup, offering a possible explanation for the low willingness to get vaccinated against influenza.

To the best of our knowledge, no prior study has included reliance on anecdotal testimonies as a variable in a vaccine hesitancy-related cluster analysis. Our results suggest that the reliance on anecdotal testimonies might be related to vaccine hesitancy, as the more vaccine-hesitant subgroups in both samples showed a stronger reliance on anecdotal testimonies than the less vaccine-hesitant subgroups. A similar pattern also emerged between the reliance on anecdotal testimonies and trust in health authorities and conspiracy mentality, as subgroups with a stronger reliance on anecdotal testimonies were in general more distrustful of health authorities and had a higher conspiracy mentality.

### Limitations

First, it is worth noting that the collected samples were convenience samples collected from social media platforms. While social media platforms such as Facebook are widely used in Finland [83], the samples were slightly biased in terms of gender and level of education, with women and people with a higher education being overrepresented [84, 85]. Second, we only focused on a few vaccine-related variables despite the plethora of factors that have been shown to be associated with vaccine hesitancy. We did, however, base our selection on theoretical models (5C, HBM, and TPB), and on recent research [18, 20–22], which have found these variables to be highly relevant for both COVID-19 and influenza vaccine hesitancy specifically. Lastly, all measures used were based on self-reports, leaving room for the possibility of response bias. We chose to measure participants’ vaccine attitudes and vaccination intentions, as obtaining data on participants’ vaccination behavior was not feasible with our current study design and with the resources at our disposal. Also, both theoretical models such as the TPB [12], as well as previous research [4] suggest that vaccine attitudes and vaccination intent can be used as proxies for actual vaccination behavior.

## Conclusions

In this study, we identified six COVID-19 and three influenza vaccine-hesitancy subgroups with unique characteristics based on variables relating to the information content that can be communicated to the subgroups to decrease vaccine hesitancy as well as the subgroups’ receptivity to communication from experts and authorities. Our results suggest that COVID-19 and influenza vaccine-hesitancy subgroups share similar vaccine attitude and belief patterns, but also that there is a greater number of COVID-19 vaccine-hesitancy subgroups than influenza vaccine-hesitancy-subgroups. These results can be used in future research to design and test tailored vaccination messages. Our study calls for bespoke vaccine messaging by demonstrating that people who hesitate in their COVID-19 and influenza vaccination decisions are not a homogenous group.

## Supporting information

S1 File Supplementary information for COVID-19 and influenza vaccine-hesitancy subgroups. (DOC)

S2 File Analysis scripts for the COVID-19 sample. (PDF)

S3 File Analysis scripts for the influenza sample. (PDF)

S4 File List of previous vaccine-hesitancy subgroup articles. (XLSX)
