# Supplementary material for: COVID-19 and influenza vaccine-hesitancy subgroups
Source: PLoS One. 2024 Jul 30;19(7):e0308159. doi: 10.1371/journal.pone.0308159 (PMC11288446; doi:10.1371/journal.pone.0308159)
Supplement: S1 File — (DOC) [file pone.0308159.s001.doc]

# Supplementary information for *COVID-19 and Influenza Vaccine Hesitancy Subgroups*

**S1 Table. Goodness-of-fit indicators: COVID-19 and influenza.**

| **Sample/Model** | **χ^2^** | ***df*** | **CFI** | **TLI** | **SRMR** | **RMSEA** |
| --- | --- | --- | --- | --- | --- | --- |
| **COVID-19** | | | | | | |
| Reliance on Anecdotal Testimonies | 96.63 | 8 | 0.97 | 0.97 | 0.04 | 0.14 |
| Trust in Health Authorities | 4.17 | 2 | 1.00 | 1.00 | 0.01 | 0.04 |
| Conspiracy Mentality | 28.74 | 4 | 1.00 | 0.99 | 0.02 | 0.11 |
| **Influenza** | | | | | | |
| Reliance on Anecdotal Testimonies | 108.78 | 8 | 0.99 | 0.98 | 0.03 | 0.15 |
| Trust in Health Authorities | 30.85 | 2 | 1.00 | 1.00 | 0.01 | 0.16 |
| Conspiracy Mentality | 52.35 | 4 | 0.99 | 0.98 | 0.03 | 0.15 |

CFI > .90 = good fit; TLI > .90 = good fit; SRMR < .08 = good fit; RMSEA < .08 = good fit (Brown, 2015).

**S2 Table. Factor loadings, variances, and error correlations: COVID-19.**

| **Factor/item** | **Unstandardized** | | **Standardized** | | |
| --- | --- | --- | --- | --- | --- |
|  | **Estimate** | ***SE*** | **Estimate** | ***SE*** | ***R^2^*** |
| **Factor loadings** | | | | | |
| Reliance on Anecdotal Testimonies | | | | | |
| FPref1* | 1.00 | - | 0.42 | 0.03 | 0.18 |
| FPref2 | 2.03 | 0.15 | 0.86 | 0.01 | 0.74 |
| FPref3 | 2.09 | 0.16 | 0.88 | 0.01 | 0.78 |
| FPref4 | 1.67 | 0.13 | 0.71 | 0.02 | 0.50 |
| FPref5 | 2.04 | 0.15 | 0.86 | 0.01 | 0.74 |
| FPref6* | 1.67 | 0.11 | 0.70 | 0.02 | 0.50 |
| Trust in Health Authorities | | | | | |
| Trust information | 1.00 | - | 0.94 | 0.01 | 0.88 |
| Trust recommendation | 0.99 | 0.01 | 0.93 | 0.01 | 0.87 |
| Trust other* | 0.79 | 0.02 | 0.74 | 0.02 | 0.55 |
| Trust intent | 0.98 | 0.01 | 0.92 | 0.01 | 0.85 |
| Conspiracy Mentality | | | | | |
| CMQ1 | 1.00 | - | 0.72 | 0.02 | 0.51 |
| CMQ2 | 0.96 | 0.03 | 0.69 | 0.02 | 0.48 |
| CMQ3 | 0.96 | 0.04 | 0.69 | 0.02 | 0.48 |
| CMQ4 | 1.28 | 0.04 | 0.92 | 0.01 | 0.85 |
| CMQ5 | 1.27 | 0.04 | 0.91 | 0.01 | 0.83 |
| **Error correlations** | | | | | |
| FPref1* ~ FPref6* | 0.32 | 0.02 | 0.50 | 0.031 | - |
| CMQ1 ~ CMQ2 | 0.27 | 0.02 | 0.53 | 0.027 | - |
| **Factor variances** | | | | | |
| Reliance on Anecdotal Testimonies | 0.18 | 0.03 | 1.00 | - | - |
| Trust in Health Authorities | 0.88 | 0.01 | 1.00 | - | - |
| Conspiracy Mentality | 0.51 | 0.04 | 1.00 | - | - |

*Reverse coded item.

**S3 Table. Factor loadings, variances, and error correlations: influenza.**

| **Factor/item** | **Unstandardized** | | **Standardized** | | |
| --- | --- | --- | --- | --- | --- |
|  | **Estimate** | ***SE*** | **Estimate** | ***SE*** | ***R^2^*** |
| **Factor loadings** | | | | | |
| Reliance on Anecdotal Testimonies | | | | | |
| FPref1* | 1.00 | - | 0.49 | 0.03 | 0.24 |
| FPref2 | 1.77 | 0.10 | 0.88 | 0.01 | 0.77 |
| FPref3 | 1.84 | 0.11 | 0.91 | 0.01 | 0.83 |
| FPref4 | 1.66 | 0.10 | 0.82 | 0.02 | 0.67 |
| FPref5 | 1.67 | 0.10 | 0.82 | 0.02 | 0.68 |
| FPref6* | 1.57 | 0.08 | 0.78 | 0.02 | 0.60 |
| Trust in Health Authorities | | | | | |
| Trust information | 1.00 | - | 0.94 | 0.01 | 0.89 |
| Trust recommendation | 1.02 | 0.01 | 0.96 | 0.01 | 0.92 |
| Trust other* | 0.89 | 0.02 | 0.83 | 0.02 | 0.69 |
| Trust intent | 1.01 | 0.01 | 0.95 | 0.01 | 0.91 |
| Conspiracy Mentality | | | | | |
| CMQ1 | 1.00 | - | 0.73 | 0.02 | 0.53 |
| CMQ2 | 1.04 | 0.03 | 0.75 | 0.02 | 0.57 |
| CMQ3 | 0.95 | 0.03 | 0.69 | 0.02 | 0.47 |
| CMQ4 | 1.26 | 0.04 | 0.91 | 0.01 | 0.83 |
| CMQ5 | 1.27 | 0.04 | 0.92 | 0.01 | 0.85 |
| **Error correlations** | | | | | |
| FPref1* ~ FPref6* | 0.27 | 0.02 | 0.49 | 0.03 | - |
| CMQ1 ~ CMQ2 | 0.22 | 0.02 | 0.48 | 0.04 | - |
| **Factor variances** | | | | | |
| Reliance on Anecdotal Testimonies | 0.24 | 0.03 | 1.00 | - | - |
| Trust in Health Authorities | 0.89 | 0.01 | 1.00 | - | - |
| Conspiracy Mentality | 0.53 | 0.03 | 1.00 | - | - |

*Reverse coded item.

**S4 Table. Duda-Hart and pseudo-*T*^2^ indexes: COVID-19.**

| **Index** | **Number of Clusters** | | | | | | | | |
| --- | --- | --- | --- | --- | --- | --- | --- | --- | --- |
|  | 2 | 3 | 4 | 5 | **6** | 7 | 8 | 9 | 10 |
| DH | 0.67 | 0.81 | 0.60 | 0.79 | **0.83** | 0.72 | 0.77 | 0.81 | 0.82 |
| Pseudo-*T*^2^ | 100.10 | 79.02 | 94.92 | 54.38 | **21.03** | 22.07 | 18.31 | 31.99 | 10.16 |

DH = Duda-Hart index, Pseudo-*T*^2^ = pseudo t squared index. Bolded = optimal cluster solution.

**S1 Fig.** **Dendrogram for the COVID-19 sample.**


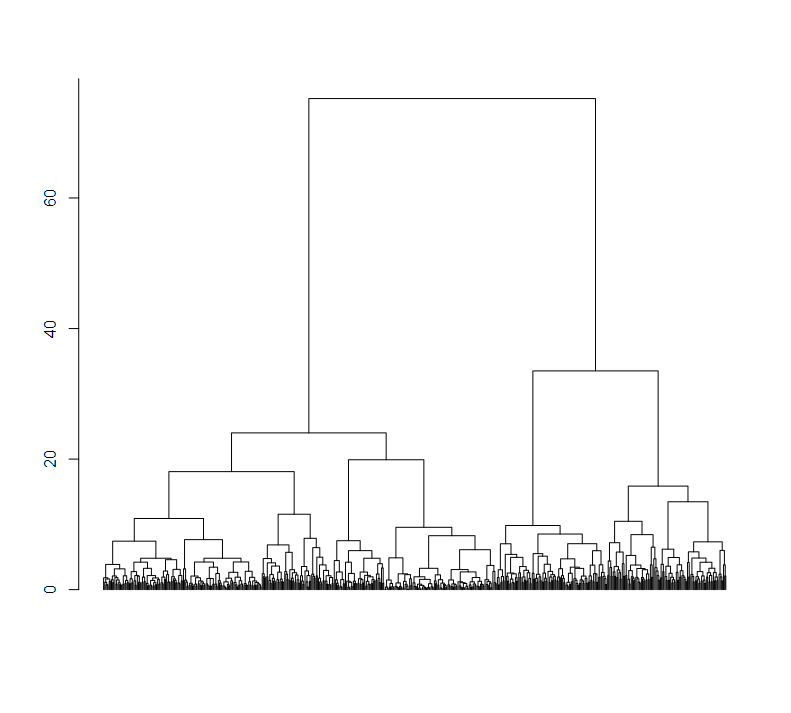


Dendrogram from the hierarchical agglomerative cluster analysis on the COVID-19 sample, based on the nine vaccine-hesitancy related factors: 1) vaccination intention, 2) perceived vaccine safety (specific and general), 3) perceived vaccine efficacy, 4) perceived disease threat, 5) perceived vaccination responsibility, 6) perceived vaccination convenience, 7) trust in health authorities, 8) conspiracy mentality, and 9) reliance on anecdotal testimonies.

**S5 Table. Means and standard deviations for unstandardized variables for the COVID-19 vaccine hesitancy subgroups.**

| **Variable** | **The Vaccination Positive** | | **The Ambivalent** | | **The Fearing Skeptic** | | **The Unconvinced** | | **The Constrained Critic** | | **The Vaccination Opponent** | |
| --- | --- | --- | --- | --- | --- | --- | --- | --- | --- | --- | --- | --- |
|  | ***M*** | ***SD*** | ***M*** | ***SD*** | ***M*** | ***SD*** | ***M*** | ***SD*** | ***M*** | ***SD*** | ***M*** | ***SD*** |
| Seasonal Vaccine Intention | 67.40 | 14.36 | 30.93 | 22.71 | 9.97 | 17.41 | 3.10 | 6.77 | 0.96 | 2.01 | 0.66 | 2.46 |
| Third Vaccine Intention | 78.58 | 17.06 | 27.88 | 24.24 | 2.88 | 5.38 | 1.19 | 3.78 | 0.31 | 0.76 | 0.89 | 6.80 |
| Perceived Vaccine Safety (General) | 5.52 | 1.05 | 3.44 | 1.42 | 1.61 | 1.00 | 1.41 | 0.69 | 1.18 | 0.44 | 1.00 | 0.00 |
| Perceived Vaccine Safety (COVID-19) | 81.47 | 12.60 | 47.65 | 18.69 | 12.84 | 12.90 | 12.17 | 13.81 | 5.20 | 15.24 | 2.23 | 5.57 |
| Perceived Vaccine Efficacy | 75.97 | 13.04 | 45.28 | 20.46 | 20.25 | 22.87 | 10.01 | 12.92 | 5.60 | 15.67 | 3.13 | 5.97 |
| Perceived Disease Threat | 55.88 | 26.27 | 39.00 | 24.16 | 61.44 | 24.96 | 12.53 | 12.14 | 12.47 | 18.99 | 2.66 | 5.50 |
| Perceived Vaccination Responsibility | 91.00 | 10.32 | 46.05 | 25.97 | 15.92 | 20.89 | 5.62 | 9.55 | 3.42 | 8.66 | 0.92 | 2.98 |
| Perceived Vaccination Convenience | 87.09 | 14.18 | 76.95 | 26.72 | 94.86 | 8.62 | 93.00 | 10.12 | 52.11 | 8.58 | 99.17 | 1.94 |
| Trust in Health Authorities | 1.22 | 0.47 | 0.50 | 0.42 | -0.14 | 0.53 | -0.21 | 0.44 | -0.70 | 0.44 | -0.87 | 0.39 |
| Conspiracy Mentality | -0.65 | 0.49 | -0.36 | 0.51 | 0.01 | 0.67 | 0.08 | 0.44 | 0.57 | 0.48 | 0.63 | 0.58 |
| Reliance on Anecdotal Testimonies | -0.33 | 0.29 | -0.07 | 0.32 | 0.17 | 0.32 | -0.16 | 0.29 | 0.22 | 0.38 | 0.43 | 0.32 |

**ANOVA and chi-square test of independence for demographic variables**

To investigate demographic differences between clusters, we conducted an analysis of variance (ANOVA) with age as the dependent variable and the hesitancy subgroups obtained in the cluster analyses as the independent variable. Statistically significant ANOVA results were followed up with pairwise t-tests. Moreover, we used Chi-square test of independence to analyze differences between hesitancy subgroups in the demographic variables gender, education level, home region, and healthcare experience in both samples, as well as history of COVID-19 infection in the COVID-19 sample, and COVID-19 or influenza vaccination status, depending on which sample the respondents belonged to. Statistically significant Chi-square test results were followed up with a closer inspection of the largest adjusted standardized residuals (Sharpe, 2015).

**Demographic differences between COVID-19 vaccine hesitancy subgroups**

For a visual overview of the demographic differences between the COVID-19 hesitancy subgroups, see Figure S1. The analysis of variance and the chi-square test of independence tests showed that there were statistically significant associations between hesitancy subgroup and gender (*p* = .004), level of education (*p* = .026), home region (*p* = .047), having had COVID-19 (*p* = .005), and COVID-19 vaccination status (*p* < .001). There was no statistically significant association between hesitancy subgroup and age (*p* = .074), nor hesitancy subgroup and healthcare experience (*p* = .574). A closer inspection of the Chi-square test residuals for gender revealed that men were underrepresented in *the Fearing Skeptic^(Cov)^* hesitancy subgroup (adjusted standardized residual = -2.87) and overrepresented in *the Constrained Critic^(Cov)^* subgroup (adjusted standardized residual = 2.69). For level of education, the Chi-square test residuals showed that respondents with lower education were underrepresented in *the Unconvinced^(Cov)^* subgroup (adjusted standardized residual = -2.78) and overrepresented in *the Constrained Critic^(Cov)^* subgroup (adjusted standardized residual = 2.31). Concerning home region, the residuals revealed that respondents living in Southwest Finland were overrepresented in *the Ambivalent^(ovC)^* subgroup (adjusted standardized residual = 2.72), and that respondents from Uusimaa overrepresented in *the Vaccination Positive^(Cov)^* subgroup (adjusted standardized residual = 2.11). Respondents that had had COVID-19 were overrepresented in *the Constrained Critic^(Cov)^* subgroup (adjusted standardized residual = 3.17) and underrepresented in *the Vaccination Positive^(Cov)^* subgroup (adjusted standardized residual = -2.11). Lastly, the respondents who were vaccinated against COVID-19 were overrepresented in *the Vaccination Positive^(Cov)^* subgroup (adjusted standardized residual = 16.13) and underrepresented in *the Unconvinced^(Cov)^* subgroup (adjusted standardized residual = -7.12).

**S2 Fig. Box plots and bar graphs over demographic variable distributions for the COVID-19 vaccine hesitancy subgroups.**


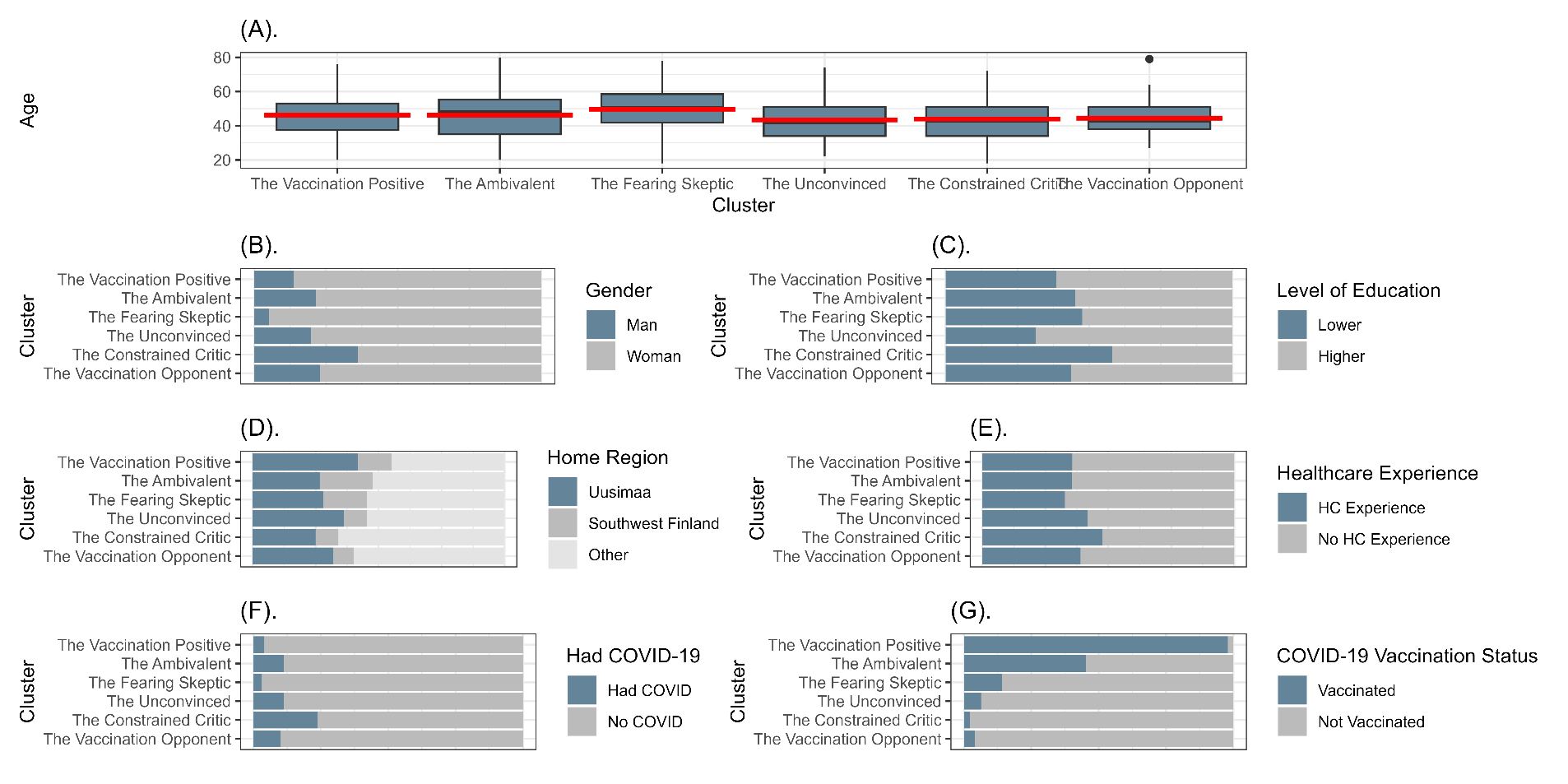
Boxplots with means for age by hesitancy subgroup (A); Bar chart for genders by hesitancy subgroup (B); Bar chart for level of education by hesitancy subgroup (C); Bar chart for home region by hesitancy subgroup (D); Bar chart for healthcare experience by hesitancy subgroup (E); Bar chart for having had a COVID-19 infection by hesitancy subgroup (F); Bar chart for COVID-19 vaccination status by hesitancy subgroup (G).

**S6 Table. Duda-Hart and pseudo-T^2^ indexes: influenza.**

| **Index** | **Number of Clusters** | | | | | | | | |
| --- | --- | --- | --- | --- | --- | --- | --- | --- | --- |
|  | 2 | **3** | 4 | 5 | 6 | 7 | 8 | 9 | 10 |
| DH | 0.74 | **0.86** | 0.82 | 0.80 | 0.74 | 0.83 | 0.81 | 0.83 | 0.75 |
| Pseudo-*T*^2^ | 89.10 | **46.48** | 37.82 | 42.33 | 45.72 | 22.82 | 25.25 | 17.75 | 18.03 |

DH = Duda-Hart index, Pseudo-*T*^2^ = pseudo t squared index. Bolded = optimal cluster solution.

**S3 Fig.** **Dendrogram for the influenza sample.**


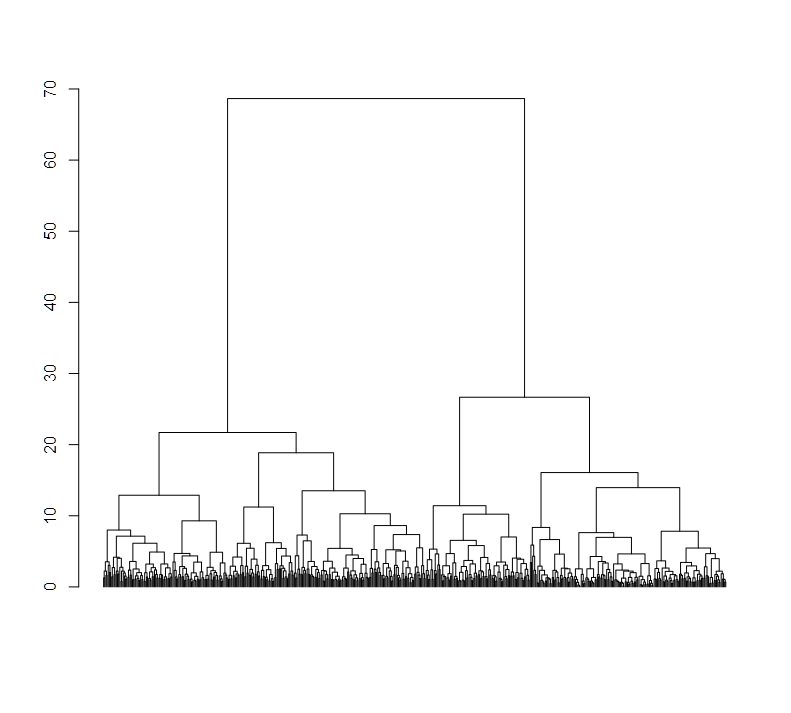


Dendrogram from the hierarchical agglomerative cluster analysis on the influenza sample, based on the nine vaccine-hesitancy related factors: 1) vaccination intention, 2) perceived vaccine safety (specific and general), 3) perceived vaccine efficacy, 4) perceived disease threat, 5) perceived vaccination responsibility, 6) perceived vaccination convenience, 7) trust in health authorities, 8) conspiracy mentality, and 9) reliance on anecdotal testimonies.

**S7 Table. Means and standard deviations for unstandardized variables for the influenza vaccine hesitancy subgroups.**

| **Variable** | **The Vaccination Positive** | | **The Complacent** | | **The Vaccination Opponent** | |
| --- | --- | --- | --- | --- | --- | --- |
|  | ***M*** | ***SD*** | ***M*** | ***SD*** | ***M*** | ***SD*** |
| Seasonal Vaccine Intention | 38.60 | 27.55 | 4.89 | 8.57 | 0.68 | 2.77 |
| Perceived Vaccine Safety (General) | 5.62 | 1.22 | 2.59 | 1.48 | 1.22 | 0.64 |
| Perceived Vaccine Safety (influenza) | 85.51 | 16.43 | 71.67 | 19.21 | 25.25 | 24.08 |
| Perceived Vaccine Efficacy | 70.25 | 20.09 | 50.67 | 23.10 | 14.58 | 14.95 |
| Perceived Disease Threat | 34.14 | 21.95 | 20.20 | 20.51 | 8.15 | 14.33 |
| Perceived Vaccination Responsibility | 62.48 | 22.83 | 26.30 | 20.68 | 3.94 | 8.08 |
| Perceived Vaccination Convenience | 78.82 | 20.77 | 85.09 | 16.77 | 85.64 | 20.67 |
| Trust in Health Authorities | 0.61 | 0.48 | -0.24 | 0.39 | -0.91 | 0.41 |
| Conspiracy Mentality | -0.43 | 0.48 | 0.11 | 0.34 | 0.68 | 0.51 |
| Reliance on Anecdotal Testimonies | -0.26 | 0.37 | 0.13 | 0.36 | 0.37 | 0.38 |

**Demographic differences between influenza vaccine hesitancy subgroups**

For a visual overview of the demographic differences between the influenza vaccine hesitancy subgroups, see Figure 2. The analysis of variance and the chi-square tests of independence showed that there were statistically significant associations between hesitancy subgroup and age (*p* = .005), gender (*p* = .002), level of education (*p* = .018), home region (*p* = .007), and influenza vaccination status (*p* < .001). There was no statistically significant association between hesitancy subgroup and healthcare experience (*p* = .077). Pair-wise comparisons revealed that there was a statistically significant difference in age between *the Vaccination Positive^(Inf)^* and *the Complacent^(Inf)^* (*p* = .006), with respondents of *the Vaccination Positive^(Inf)^* subgroup being slightly older. Inspecting the Chi-square test residuals for gender showed that men were overrepresented in *the Vaccination Opponent^(Inf)^* subgroup (adjusted standardized residual = 3.38) and underrepresented in *the Complacent^(I)^* subgroup (adjusted standardized residual = -2.11). Concerning the level of education, the Chi-square test residuals showed that respondents with lower education overrepresented in *the Vaccination Opponent^(Inf)^* subgroup (adjusted standardized residual = 2.51) and underrepresented in *the Vaccination Positive^(Inf)^* subgroup (adjusted standardized residual = -2.70). Regarding home region, the residuals revealed that respondents living in Southwest Finland were underrepresented in *the Vaccination Opponent^(Inf)^* subgroup (adjusted standardized residual = -2.70) and overrepresented in *the Complacent^(Inf)^* subgroup (adjusted standardized residual = 2.25). Finally, respondents who had taken the latest influenza vaccine were overrepresented in *the Vaccination Positive^(Inf)^* subgroup (adjusted standardized residual = 5.09) and underrepresented in the Vaccination Opponent^(Inf)^ subgroup (adjusted standardized residual = -4.07).

**S4 Fig. Box plots and bar graphs over demographic variable distributions for the influenza vaccine hesitancy subgroups.**


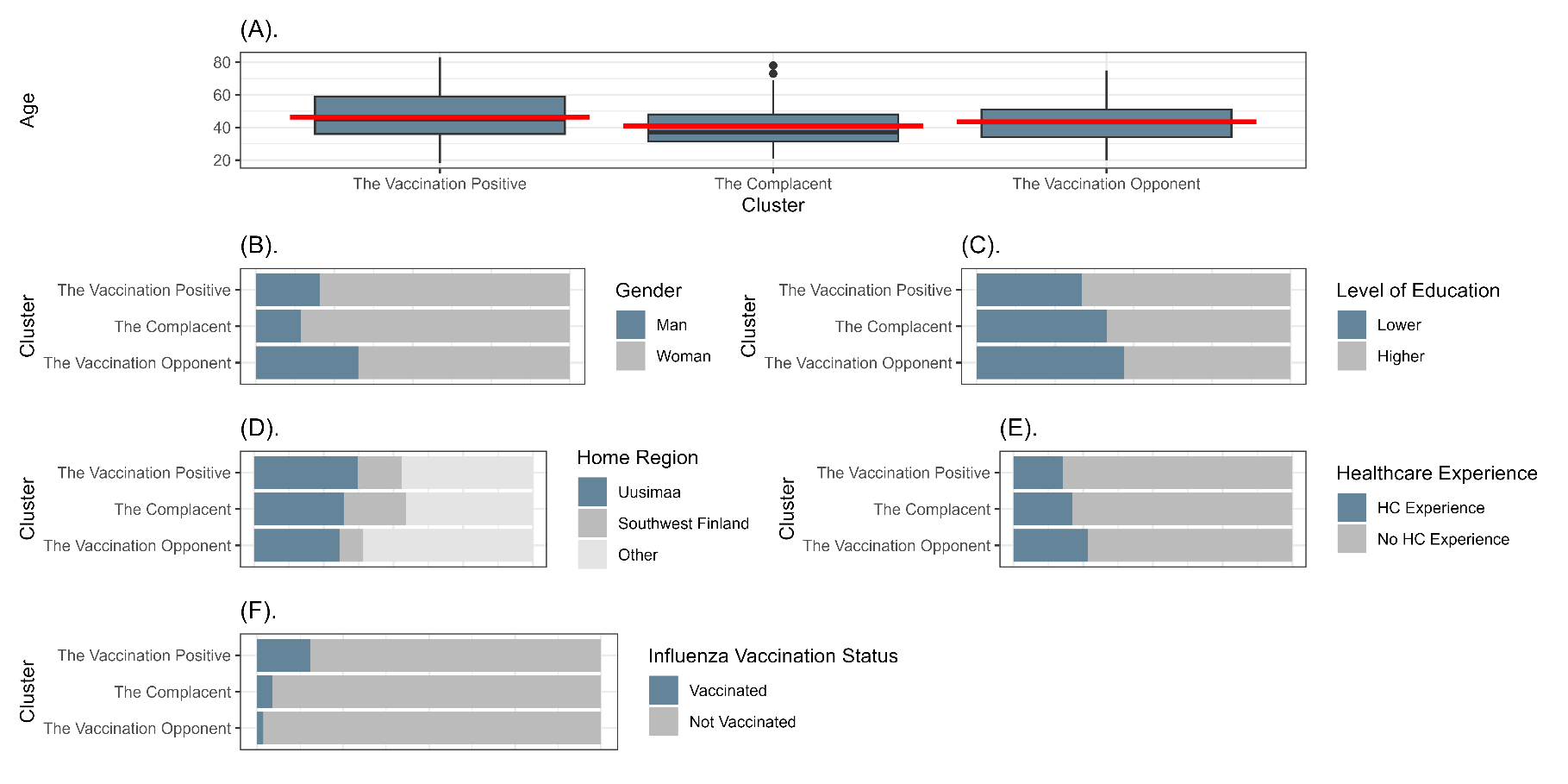


Box plots with means for age by hesitancy subgroup (A); Bar chart for genders by hesitancy subgroup (B); Bar chart for level of education by hesitancy subgroup (C); Bar chart for home region by hesitancy subgroup (D); Bar chart for healthcare experience by hesitancy subgroup (E); Bar chart for influenza vaccination status by hesitancy subgroup (F).

**References**

1. Brown T. Confirmatory for Analysis for Applied Research. Kenny DA, Little ToddD, editors. 2015; 462.

2. Sharpe D. Chi-Square Test is Statistically Significant: Now What? Practical Assessment, Research, and Evaluation Practical Assessment. 2015;20. doi:10.7275/tbfa-x148
