# Supplementary material for: COVID-19 and influenza vaccine-hesitancy subgroups
Source: PLoS One. 2024 Jul 30;19(7):e0308159. doi: 10.1371/journal.pone.0308159 (PMC11288446; doi:10.1371/journal.pone.0308159)
Supplement: S2 File — (PDF) [file pone.0308159.s002.pdf]

COVID2021 Sample Analyses

Preparations

Load packages

```r
library(tidyverse)
```

```
## ── Attaching core tidyverse packages ──────────────────────── tidyverse 2.0.0 ──
## ✔ dplyr     1.1.2     ✔ readr     2.1.4
## ✔ forcats   1.0.0     ✔ stringr   1.5.0
## ✔ ggplot2   3.4.2     ✔ tibble    3.2.1
## ✔ lubridate 1.9.2     ✔ tidyr     1.3.0
## ✔ purrr     1.0.1
## ── Conflicts ────────────────────────────────────────── tidyverse_conflicts() ──
## ✖ dplyr::filter() masks stats::filter()
## ✖ dplyr::lag()    masks stats::lag()
## ℹ Use the conflicted package (<http://conflicted.r-lib.org/>) to force all conflicts to become errors
```

```r
library(lavaan)
```

```
## This is lavaan 0.6-15
## lavaan is FREE software! Please report any bugs.
```

```r
library(NbClust)
library(RcmdrMisc)
```

```
## Loading required package: car
## Loading required package: carData
##
## Attaching package: 'car'
##
## The following object is masked from 'package:dplyr':
##
##     recode
##
## The following object is masked from 'package:purrr':
##
##     some
##
## Loading required package: sandwich
```

```r
library(GGally)
```

```
## Registered S3 method overwritten by 'GGally':
##   method from
##   +.gg   ggplot2
```

```r
library(broom)
library(questionr)
library(patchwork)
library(rstatix)
```

```
##
## Attaching package: 'rstatix'
##
## The following object is masked from 'package:stats':
##
##     filter
```

```r
library(kableExtra)
```

```
##
## Attaching package: 'kableExtra'
##
## The following object is masked from 'package:dplyr':
##
##     group_rows
```

Import data

```r
# Import Data File Using Relative Path
tib <- read.csv2("./data/processed_data/COVID2021_HCA.csv") %>%
  tibble()
```

Factor nominal variables

```r
tib <- tib %>%
  mutate(across(c(sex,
                  education,
                  live,
                  hcw,
                  subject,
                  c_vaccine_taken,
                  c_vaccination_experience,
                  had_covid),
                factor))
```

Confirmatory Factor Analyses

Format Preference

```r
# Create the model
format_preference <- "format_preference =~ abq1_rev + abq2 + abq3 + abq4 + abq5 + abq6_rev
                      abq1_rev ~~ abq6_rev"

# Conduct the CFA
m_format_preference <- cfa(format_preference,
                           estimator = "WLSMV",
                           missing = "pairwise",
                           ordered = TRUE,
                           data = tib)

# Show the results
summary(m_format_preference,
        fit.measures = TRUE,
        standardized = TRUE,
        rsquare = TRUE)
```

```
## lavaan 0.6.15 ended normally after 32 iterations
##
##   Estimator                                       DWLS
##   Optimization method                           NLMINB
##   Number of model parameters                        43
##
##   Number of observations                           559
##   Number of missing patterns                         4
##
## Model Test User Model:
##                                               Standard      Scaled
##   Test Statistic                                32.182      96.626
##   Degrees of freedom                                 8           8
##   P-value (Chi-square)                           0.000       0.000
##   Scaling correction factor                                  0.337
##   Shift parameter                                            1.002
##     simple second-order correction
##
## Model Test Baseline Model:
##
##   Test statistic                             12066.279    6190.980
##   Degrees of freedom                                15          15
##   P-value                                        0.000       0.000
##   Scaling correction factor                                  1.951
##
## User Model versus Baseline Model:
##
##   Comparative Fit Index (CFI)                    0.998       0.986
##   Tucker-Lewis Index (TLI)                       0.996       0.973
##
##   Robust Comparative Fit Index (CFI)                         0.972
##   Robust Tucker-Lewis Index (TLI)                            0.947
##
## Root Mean Square Error of Approximation:
##
##   RMSEA                                          0.074       0.141
##   90 Percent confidence interval - lower         0.048       0.117
##   90 Percent confidence interval - upper         0.101       0.167
##   P-value H_0: RMSEA <= 0.050                    0.062       0.000
##   P-value H_0: RMSEA >= 0.080                    0.378       1.000
##
##   Robust RMSEA                                               0.113
##   90 Percent confidence interval - lower                     0.092
##   90 Percent confidence interval - upper                     0.135
##   P-value H_0: Robust RMSEA <= 0.050                         0.000
##   P-value H_0: Robust RMSEA >= 0.080                         0.994
##
## Standardized Root Mean Square Residual:
##
##   SRMR                                           0.035       0.035
##
## Parameter Estimates:
##
##   Standard errors                           Robust.sem
##   Information                                 Expected
##   Information saturated (h1) model        Unstructured
##
## Latent Variables:
##                        Estimate  Std.Err  z-value  P(>|z|)   Std.lv  Std.all
##   format_preference =~
##     abq1_rev              1.000                               0.423    0.423
##     abq2                  2.029    0.152   13.388    0.000    0.858    0.858
##     abq3                  2.090    0.155   13.445    0.000    0.883    0.883
##     abq4                  1.671    0.130   12.873    0.000    0.706    0.706
##     abq5                  2.039    0.149   13.710    0.000    0.862    0.862
##     abq6_rev              1.666    0.113   14.777    0.000    0.704    0.704
##
## Covariances:
##                    Estimate  Std.Err  z-value  P(>|z|)   Std.lv  Std.all
##  .abq1_rev ~~
##    .abq6_rev          0.321    0.024   13.364    0.000    0.321    0.498
##
## Intercepts:
##                    Estimate  Std.Err  z-value  P(>|z|)   Std.lv  Std.all
##    .abq1_rev          0.000                               0.000    0.000
##    .abq2              0.000                               0.000    0.000
##    .abq3              0.000                               0.000    0.000
##    .abq4              0.000                               0.000    0.000
##    .abq5              0.000                               0.000    0.000
##    .abq6_rev          0.000                               0.000    0.000
##     format_prefrnc    0.000                               0.000    0.000
##
## Thresholds:
##                    Estimate  Std.Err  z-value  P(>|z|)   Std.lv  Std.all
##     abq1_rev|t1      -1.259    0.072  -17.580    0.000   -1.259   -1.259
##     abq1_rev|t2      -0.558    0.056   -9.920    0.000   -0.558   -0.558
##     abq1_rev|t3       0.004    0.053    0.085    0.933    0.004    0.004
##     abq1_rev|t4       0.532    0.056    9.506    0.000    0.532    0.532
##     abq1_rev|t5       0.918    0.062   14.788    0.000    0.918    0.918
##     abq1_rev|t6       1.269    0.072   17.632    0.000    1.269    1.269
##     abq2|t1          -1.531    0.083  -18.377    0.000   -1.531   -1.531
##     abq2|t2          -0.831    0.060  -13.765    0.000   -0.831   -0.831
##     abq2|t3          -0.337    0.054   -6.211    0.000   -0.337   -0.337
##     abq2|t4           0.257    0.054    4.779    0.000    0.257    0.257
##     abq2|t5           0.687    0.058   11.854    0.000    0.687    0.687
##     abq2|t6           1.229    0.071   17.396    0.000    1.229    1.229
##     abq3|t1          -1.367    0.076  -18.067    0.000   -1.367   -1.367
##     abq3|t2          -0.586    0.056  -10.365    0.000   -0.586   -0.586
##     abq3|t3          -0.065    0.053   -1.225    0.220   -0.065   -0.065
##     abq3|t4           0.570    0.056   10.118    0.000    0.570    0.570
##     abq3|t5           0.983    0.064   15.478    0.000    0.983    0.983
##     abq3|t6           1.491    0.081   18.368    0.000    1.491    1.491
##     abq4|t1          -1.028    0.065  -15.906    0.000   -1.028   -1.028
##     abq4|t2          -0.247    0.054   -4.602    0.000   -0.247   -0.247
##     abq4|t3           0.183    0.053    3.421    0.001    0.183    0.183
##     abq4|t4           0.784    0.059   13.195    0.000    0.784    0.784
##     abq4|t5           1.291    0.073   17.754    0.000    1.291    1.291
##     abq4|t6           1.849    0.104   17.854    0.000    1.849    1.849
##     abq5|t1          -1.451    0.079  -18.300    0.000   -1.451   -1.451
##     abq5|t2          -0.736    0.059  -12.559    0.000   -0.736   -0.736
##     abq5|t3          -0.350    0.054   -6.452    0.000   -0.350   -0.350
##     abq5|t4           0.369    0.054    6.788    0.000    0.369    0.369
##     abq5|t5           0.828    0.060   13.744    0.000    0.828    0.828
##     abq5|t6           1.260    0.072   17.601    0.000    1.260    1.260
##     abq6_rev|t1      -1.609    0.087  -18.384    0.000   -1.609   -1.609
##     abq6_rev|t2      -0.788    0.060  -13.216    0.000   -0.788   -0.788
##     abq6_rev|t3      -0.252    0.054   -4.694    0.000   -0.252   -0.252
##     abq6_rev|t4       0.444    0.055    8.057    0.000    0.444    0.444
##     abq6_rev|t5       0.844    0.061   13.921    0.000    0.844    0.844
##     abq6_rev|t6       1.310    0.074   17.810    0.000    1.310    1.310
##
## Variances:
##                    Estimate  Std.Err  z-value  P(>|z|)   Std.lv  Std.all
##    .abq1_rev          0.821                               0.821    0.821
##    .abq2              0.265                               0.265    0.265
##    .abq3              0.219                               0.219    0.219
##    .abq4              0.501                               0.501    0.501
##    .abq5              0.257                               0.257    0.257
##    .abq6_rev          0.504                               0.504    0.504
##     format_prefrnc    0.179    0.027    6.589    0.000    1.000    1.000
##
## Scales y*:
##                    Estimate  Std.Err  z-value  P(>|z|)   Std.lv  Std.all
##     abq1_rev          1.000                               1.000    1.000
##     abq2              1.000                               1.000    1.000
##     abq3              1.000                               1.000    1.000
##     abq4              1.000                               1.000    1.000
##     abq5              1.000                               1.000    1.000
##     abq6_rev          1.000                               1.000    1.000
##
## R-Square:
##                    Estimate
##     abq1_rev          0.179
##     abq2              0.735
##     abq3              0.781
##     abq4              0.499
##     abq5              0.743
##     abq6_rev          0.496
```

```r
# Show the standardized results
format_preference_loadings <- standardizedsolution(m_format_preference)

format_preference_loadings
```

```
##                  lhs  op               rhs est.std    se       z pvalue
## 1  format_preference  =~          abq1_rev   0.423 0.032  13.179  0.000
## 2  format_preference  =~              abq2   0.858 0.012  71.855  0.000
## 3  format_preference  =~              abq3   0.883 0.012  76.775  0.000
## 4  format_preference  =~              abq4   0.706 0.020  35.409  0.000
## 5  format_preference  =~              abq5   0.862 0.012  72.767  0.000
## 6  format_preference  =~          abq6_rev   0.704 0.020  35.479  0.000
## 7           abq1_rev  ~~          abq6_rev   0.498 0.031  16.222  0.000
## 8           abq1_rev   |                t1  -1.259 0.072 -17.580  0.000
## 9           abq1_rev   |                t2  -0.558 0.056  -9.920  0.000
## 10          abq1_rev   |                t3   0.004 0.053   0.085  0.933
## 11          abq1_rev   |                t4   0.532 0.056   9.506  0.000
## 12          abq1_rev   |                t5   0.918 0.062  14.788  0.000
## 13          abq1_rev   |                t6   1.269 0.072  17.632  0.000
## 14              abq2   |                t1  -1.531 0.083 -18.377  0.000
## 15              abq2   |                t2  -0.831 0.060 -13.765  0.000
## 16              abq2   |                t3  -0.337 0.054  -6.211  0.000
## 17              abq2   |                t4   0.257 0.054   4.779  0.000
## 18              abq2   |                t5   0.687 0.058  11.854  0.000
## 19              abq2   |                t6   1.229 0.071  17.396  0.000
## 20              abq3   |                t1  -1.367 0.076 -18.067  0.000
## 21              abq3   |                t2  -0.586 0.056 -10.365  0.000
## 22              abq3   |                t3  -0.065 0.053  -1.225  0.220
## 23              abq3   |                t4   0.570 0.056  10.118  0.000
## 24              abq3   |                t5   0.983 0.064  15.478  0.000
## 25              abq3   |                t6   1.491 0.081  18.368  0.000
## 26              abq4   |                t1  -1.028 0.065 -15.906  0.000
## 27              abq4   |                t2  -0.247 0.054  -4.602  0.000
## 28              abq4   |                t3   0.183 0.053   3.421  0.001
## 29              abq4   |                t4   0.784 0.059  13.195  0.000
## 30              abq4   |                t5   1.291 0.073  17.754  0.000
## 31              abq4   |                t6   1.849 0.104  17.854  0.000
## 32              abq5   |                t1  -1.451 0.079 -18.300  0.000
## 33              abq5   |                t2  -0.736 0.059 -12.559  0.000
## 34              abq5   |                t3  -0.350 0.054  -6.452  0.000
## 35              abq5   |                t4   0.369 0.054   6.788  0.000
## 36              abq5   |                t5   0.828 0.060  13.744  0.000
## 37              abq5   |                t6   1.260 0.072  17.601  0.000
## 38          abq6_rev   |                t1  -1.609 0.087 -18.384  0.000
## 39          abq6_rev   |                t2  -0.788 0.060 -13.216  0.000
## 40          abq6_rev   |                t3  -0.252 0.054  -4.694  0.000
## 41          abq6_rev   |                t4   0.444 0.055   8.057  0.000
## 42          abq6_rev   |                t5   0.844 0.061  13.921  0.000
## 43          abq6_rev   |                t6   1.310 0.074  17.810  0.000
## 44          abq1_rev  ~~          abq1_rev   0.821 0.027  30.277  0.000
## 45              abq2  ~~              abq2   0.265 0.020  12.921  0.000
## 46              abq3  ~~              abq3   0.219 0.020  10.793  0.000
## 47              abq4  ~~              abq4   0.501 0.028  17.777  0.000
## 48              abq5  ~~              abq5   0.257 0.020  12.561  0.000
## 49          abq6_rev  ~~          abq6_rev   0.504 0.028  18.037  0.000
## 50 format_preference  ~~ format_preference   1.000 0.000      NA     NA
## 51          abq1_rev ~*~          abq1_rev   1.000 0.000      NA     NA
## 52              abq2 ~*~              abq2   1.000 0.000      NA     NA
## 53              abq3 ~*~              abq3   1.000 0.000      NA     NA
## 54              abq4 ~*~              abq4   1.000 0.000      NA     NA
## 55              abq5 ~*~              abq5   1.000 0.000      NA     NA
## 56          abq6_rev ~*~          abq6_rev   1.000 0.000      NA     NA
## 57          abq1_rev  ~1                     0.000 0.000      NA     NA
## 58              abq2  ~1                     0.000 0.000      NA     NA
## 59              abq3  ~1                     0.000 0.000      NA     NA
## 60              abq4  ~1                     0.000 0.000      NA     NA
## 61              abq5  ~1                     0.000 0.000      NA     NA
## 62          abq6_rev  ~1                     0.000 0.000      NA     NA
## 63 format_preference  ~1                     0.000 0.000      NA     NA
##    ci.lower ci.upper
## 1     0.360    0.486
## 2     0.834    0.881
## 3     0.861    0.906
## 4     0.667    0.745
## 5     0.839    0.885
## 6     0.665    0.743
## 7     0.438    0.559
## 8    -1.400   -1.119
## 9    -0.668   -0.447
## 10   -0.100    0.109
## 11    0.422    0.641
## 12    0.797    1.040
## 13    1.128    1.410
## 14   -1.695   -1.368
## 15   -0.950   -0.713
## 16   -0.443   -0.231
## 17    0.152    0.363
## 18    0.574    0.801
## 19    1.091    1.368
## 20   -1.515   -1.218
## 21   -0.696   -0.475
## 22   -0.169    0.039
## 23    0.459    0.680
## 24    0.859    1.108
## 25    1.332    1.650
## 26   -1.154   -0.901
## 27   -0.352   -0.142
## 28    0.078    0.287
## 29    0.668    0.901
## 30    1.148    1.433
## 31    1.646    2.052
## 32   -1.607   -1.296
## 33   -0.851   -0.621
## 34   -0.456   -0.244
## 35    0.263    0.476
## 36    0.710    0.946
## 37    1.120    1.401
## 38   -1.780   -1.437
## 39   -0.905   -0.671
## 40   -0.358   -0.147
## 41    0.336    0.552
## 42    0.725    0.963
## 43    1.166    1.454
## 44    0.768    0.874
## 45    0.224    0.305
## 46    0.180    0.259
## 47    0.446    0.556
## 48    0.217    0.297
## 49    0.449    0.559
## 50    1.000    1.000
## 51    1.000    1.000
## 52    1.000    1.000
## 53    1.000    1.000
## 54    1.000    1.000
## 55    1.000    1.000
## 56    1.000    1.000
## 57    0.000    0.000
## 58    0.000    0.000
## 59    0.000    0.000
## 60    0.000    0.000
## 61    0.000    0.000
## 62    0.000    0.000
## 63    0.000    0.000
```

```r
# Calculate factor scores
format_preference_score <- predict(m_format_preference)

# Add the calculated factor scores to the data file
tib <- cbind(tib, format_preference_score)
```

Trust in Health Authorities

```r
# Create the model
trust_hca <- "trust_hca =~ trust_info + trust_recommend + trust_other_rev + trust_intent"

# Conduct the CFA
m_trust_hca <- cfa(trust_hca,
                   estimator = "WLSMV",
                   missing = "pairwise",
                   ordered = TRUE,
                   data = tib)
```

```
## Warning in lav_data_full(data = data, group = group, cluster = cluster, : lavaan WARNING: some cases are empty
and will be ignored:
##   436
```

```r
# Show the results
summary(m_trust_hca,
        fit.measures = TRUE,
        standardized = TRUE,
        rsquare = TRUE)
```

```
## lavaan 0.6.15 ended normally after 15 iterations
##
##   Estimator                                       DWLS
##   Optimization method                           NLMINB
##   Number of model parameters                        28
##
##                                                   Used       Total
##   Number of observations                           558         559
##   Number of missing patterns                         4
##
## Model Test User Model:
##                                               Standard      Scaled
##   Test Statistic                                 0.643       4.172
##   Degrees of freedom                                 2           2
##   P-value (Chi-square)                           0.725       0.124
##   Scaling correction factor                                  0.155
##   Shift parameter                                            0.021
##     simple second-order correction
##
## Model Test Baseline Model:
##
##   Test statistic                             21777.448   13233.947
##   Degrees of freedom                                 6           6
##   P-value                                        0.000       0.000
##   Scaling correction factor                                  1.646
##
## User Model versus Baseline Model:
##
##   Comparative Fit Index (CFI)                    1.000       1.000
##   Tucker-Lewis Index (TLI)                       1.000       1.000
##
##   Robust Comparative Fit Index (CFI)                         0.999
##   Robust Tucker-Lewis Index (TLI)                            0.998
##
## Root Mean Square Error of Approximation:
##
##   RMSEA                                          0.000       0.044
##   90 Percent confidence interval - lower         0.000       0.000
##   90 Percent confidence interval - upper         0.060       0.105
##   P-value H_0: RMSEA <= 0.050                    0.916       0.465
##   P-value H_0: RMSEA >= 0.080                    0.013       0.198
##
##   Robust RMSEA                                               0.037
##   90 Percent confidence interval - lower                     0.000
##   90 Percent confidence interval - upper                     0.085
##   P-value H_0: Robust RMSEA <= 0.050                         0.597
##   P-value H_0: Robust RMSEA >= 0.080                         0.075
##
## Standardized Root Mean Square Residual:
##
##   SRMR                                           0.005       0.005
##
## Parameter Estimates:
##
##   Standard errors                           Robust.sem
##   Information                                 Expected
##   Information saturated (h1) model        Unstructured
##
## Latent Variables:
##                    Estimate  Std.Err  z-value  P(>|z|)   Std.lv  Std.all
##   trust_hca =~
##     trust_info        1.000                               0.938    0.938
##     trust_recommnd    0.993    0.011   88.340    0.000    0.931    0.931
##     trust_other_rv    0.790    0.019   40.546    0.000    0.740    0.740
##     trust_intent      0.982    0.009  113.988    0.000    0.921    0.921
##
## Intercepts:
##                    Estimate  Std.Err  z-value  P(>|z|)   Std.lv  Std.all
##    .trust_info        0.000                               0.000    0.000
##    .trust_recommnd    0.000                               0.000    0.000
##    .trust_other_rv    0.000                               0.000    0.000
##    .trust_intent      0.000                               0.000    0.000
##     trust_hca         0.000                               0.000    0.000
##
## Thresholds:
##                    Estimate  Std.Err  z-value  P(>|z|)   Std.lv  Std.all
##     trust_info|t1    -0.347    0.054   -6.379    0.000   -0.347   -0.347
##     trust_info|t2     0.020    0.053    0.381    0.703    0.020    0.020
##     trust_info|t3     0.361    0.054    6.632    0.000    0.361    0.361
##     trust_info|t4     0.687    0.058   11.854    0.000    0.687    0.687
##     trust_info|t5     0.931    0.062   14.911    0.000    0.931    0.931
##     trust_info|t6     1.503    0.082   18.350    0.000    1.503    1.503
##     trst_rcmmnd|t1   -0.332    0.054   -6.127    0.000   -0.332   -0.332
##     trst_rcmmnd|t2    0.056    0.053    1.058    0.290    0.056    0.056
##     trst_rcmmnd|t3    0.434    0.055    7.890    0.000    0.434    0.434
##     trst_rcmmnd|t4    0.687    0.058   11.854    0.000    0.687    0.687
##     trst_rcmmnd|t5    1.064    0.066   16.203    0.000    1.064    1.064
##     trst_rcmmnd|t6    1.609    0.087   18.383    0.000    1.609    1.609
##     trst_thr_rv|t1   -0.898    0.062  -14.562    0.000   -0.898   -0.898
##     trst_thr_rv|t2   -0.558    0.056   -9.920    0.000   -0.558   -0.558
##     trst_thr_rv|t3   -0.199    0.053   -3.720    0.000   -0.199   -0.199
##     trst_thr_rv|t4    0.392    0.055    7.171    0.000    0.392    0.392
##     trst_thr_rv|t5    0.717    0.058   12.288    0.000    0.717    0.717
##     trst_thr_rv|t6    1.230    0.071   17.417    0.000    1.230    1.230
##     trust_intnt|t1   -0.593    0.057  -10.465    0.000   -0.593   -0.593
##     trust_intnt|t2   -0.192    0.054   -3.596    0.000   -0.192   -0.192
##     trust_intnt|t3    0.147    0.053    2.751    0.006    0.147    0.147
##     trust_intnt|t4    0.572    0.056   10.135    0.000    0.572    0.572
##     trust_intnt|t5    0.904    0.062   14.611    0.000    0.904    0.904
##     trust_intnt|t6    1.424    0.078   18.206    0.000    1.424    1.424
##
## Variances:
##                    Estimate  Std.Err  z-value  P(>|z|)   Std.lv  Std.all
##    .trust_info        0.121                               0.121    0.121
##    .trust_recommnd    0.132                               0.132    0.132
##    .trust_other_rv    0.452                               0.452    0.452
##    .trust_intent      0.153                               0.153    0.153
##     trust_hca         0.879    0.014   64.982    0.000    1.000    1.000
##
## Scales y*:
##                    Estimate  Std.Err  z-value  P(>|z|)   Std.lv  Std.all
##     trust_info        1.000                               1.000    1.000
##     trust_recommnd    1.000                               1.000    1.000
##     trust_other_rv    1.000                               1.000    1.000
##     trust_intent      1.000                               1.000    1.000
##
## R-Square:
##                    Estimate
##     trust_info        0.879
##     trust_recommnd    0.868
##     trust_other_rv    0.548
##     trust_intent      0.847
```

```r
# Show the standardized results
trust_loadings <- standardizedsolution(m_trust_hca)

trust_loadings
```

```
##                lhs  op             rhs est.std    se       z pvalue ci.lower
## 1        trust_hca  =~      trust_info   0.938 0.007 129.963  0.000    0.924
## 2        trust_hca  =~ trust_recommend   0.931 0.008 114.338  0.000    0.915
## 3        trust_hca  =~ trust_other_rev   0.740 0.019  38.589  0.000    0.703
## 4        trust_hca  =~    trust_intent   0.921 0.008 120.632  0.000    0.906
## 5       trust_info   |              t1  -0.347 0.054  -6.379  0.000   -0.453
## 6       trust_info   |              t2   0.020 0.053   0.381  0.703   -0.084
## 7       trust_info   |              t3   0.361 0.054   6.632  0.000    0.254
## 8       trust_info   |              t4   0.687 0.058  11.854  0.000    0.574
## 9       trust_info   |              t5   0.931 0.062  14.911  0.000    0.809
## 10      trust_info   |              t6   1.503 0.082  18.350  0.000    1.342
## 11 trust_recommend   |              t1  -0.332 0.054  -6.127  0.000   -0.439
## 12 trust_recommend   |              t2   0.056 0.053   1.058  0.290   -0.048
## 13 trust_recommend   |              t3   0.434 0.055   7.890  0.000    0.326
## 14 trust_recommend   |              t4   0.687 0.058  11.854  0.000    0.574
## 15 trust_recommend   |              t5   1.064 0.066  16.203  0.000    0.935
## 16 trust_recommend   |              t6   1.609 0.087  18.383  0.000    1.437
## 17 trust_other_rev   |              t1  -0.898 0.062 -14.562  0.000   -1.019
## 18 trust_other_rev   |              t2  -0.558 0.056  -9.920  0.000   -0.668
## 19 trust_other_rev   |              t3  -0.199 0.053  -3.720  0.000   -0.304
## 20 trust_other_rev   |              t4   0.392 0.055   7.171  0.000    0.285
## 21 trust_other_rev   |              t5   0.717 0.058  12.288  0.000    0.603
## 22 trust_other_rev   |              t6   1.230 0.071  17.417  0.000    1.092
## 23    trust_intent   |              t1  -0.593 0.057 -10.465  0.000   -0.704
## 24    trust_intent   |              t2  -0.192 0.054  -3.596  0.000   -0.297
## 25    trust_intent   |              t3   0.147 0.053   2.751  0.006    0.042
## 26    trust_intent   |              t4   0.572 0.056  10.135  0.000    0.461
## 27    trust_intent   |              t5   0.904 0.062  14.611  0.000    0.782
## 28    trust_intent   |              t6   1.424 0.078  18.206  0.000    1.271
## 29      trust_info  ~~      trust_info   0.121 0.014   8.903  0.000    0.094
## 30 trust_recommend  ~~ trust_recommend   0.132 0.015   8.723  0.000    0.103
## 31 trust_other_rev  ~~ trust_other_rev   0.452 0.028  15.901  0.000    0.396
## 32    trust_intent  ~~    trust_intent   0.153 0.014  10.859  0.000    0.125
## 33       trust_hca  ~~       trust_hca   1.000 0.000      NA     NA    1.000
## 34      trust_info ~*~      trust_info   1.000 0.000      NA     NA    1.000
## 35 trust_recommend ~*~ trust_recommend   1.000 0.000      NA     NA    1.000
## 36 trust_other_rev ~*~ trust_other_rev   1.000 0.000      NA     NA    1.000
## 37    trust_intent ~*~    trust_intent   1.000 0.000      NA     NA    1.000
## 38      trust_info  ~1                   0.000 0.000      NA     NA    0.000
## 39 trust_recommend  ~1                   0.000 0.000      NA     NA    0.000
## 40 trust_other_rev  ~1                   0.000 0.000      NA     NA    0.000
## 41    trust_intent  ~1                   0.000 0.000      NA     NA    0.000
## 42       trust_hca  ~1                   0.000 0.000      NA     NA    0.000
##    ci.upper
## 1     0.952
## 2     0.947
## 3     0.778
## 4     0.936
## 5    -0.240
## 6     0.124
## 7     0.468
## 8     0.801
## 9     1.053
## 10    1.663
## 11   -0.226
## 12    0.161
## 13    0.542
## 14    0.801
## 15    1.193
## 16    1.780
## 17   -0.777
## 18   -0.447
## 19   -0.094
## 20    0.499
## 21    0.832
## 22    1.369
## 23   -0.482
## 24   -0.088
## 25    0.251
## 26    0.683
## 27    1.025
## 28    1.577
## 29    0.147
## 30    0.162
## 31    0.507
## 32    0.180
## 33    1.000
## 34    1.000
## 35    1.000
## 36    1.000
## 37    1.000
## 38    0.000
## 39    0.000
## 40    0.000
## 41    0.000
## 42    0.000
```

```r
# Calculate factor scores
trust_hca_score <- predict(m_trust_hca)

# Add the calculated factor scores to the data file
tib <- cbind(tib, trust_hca_score)
```

Conspiracy Mentality

```r
# Create the model
conspiracy_mentality <- "conspiracy_mentality =~ cmq1 + cmq2 + cmq3 + cmq4 + cmq5
                         cmq1 ~~ cmq2"

# Conduct the CFA
m_conspiracy_mentality <- cfa(conspiracy_mentality,
                              estimator = "WLSMV",
                              missing = "pairwise",
                              ordered = TRUE,
                              data = tib)
```

```
## Warning in lav_data_full(data = data, group = group, cluster = cluster, : lavaan WARNING: some cases are empty
and will be ignored:
##   436
```

```r
# Show the results
summary(m_conspiracy_mentality,
        fit.measures = TRUE,
        standardized = TRUE,
        rsquare = TRUE)
```

```
## lavaan 0.6.15 ended normally after 21 iterations
##
##   Estimator                                       DWLS
##   Optimization method                           NLMINB
##   Number of model parameters                        55
##
##                                                   Used       Total
##   Number of observations                           558         559
##   Number of missing patterns                         3
##
## Model Test User Model:
##                                               Standard      Scaled
##   Test Statistic                                 9.255      28.742
##   Degrees of freedom                                 4           4
##   P-value (Chi-square)                           0.055       0.000
##   Scaling correction factor                                  0.329
##   Shift parameter                                            0.620
##     simple second-order correction
##
## Model Test Baseline Model:
##
##   Test statistic                             11984.617    6753.968
##   Degrees of freedom                                10          10
##   P-value                                        0.000       0.000
##   Scaling correction factor                                  1.776
##
## User Model versus Baseline Model:
##
##   Comparative Fit Index (CFI)                    1.000       0.996
##   Tucker-Lewis Index (TLI)                       0.999       0.991
##
##   Robust Comparative Fit Index (CFI)                         0.982
##   Robust Tucker-Lewis Index (TLI)                            0.956
##
## Root Mean Square Error of Approximation:
##
##   RMSEA                                          0.049       0.105
##   90 Percent confidence interval - lower         0.000       0.071
##   90 Percent confidence interval - upper         0.090       0.143
##   P-value H_0: RMSEA <= 0.050                    0.455       0.005
##   P-value H_0: RMSEA >= 0.080                    0.118       0.895
##
##   Robust RMSEA                                               0.122
##   90 Percent confidence interval - lower                     0.091
##   90 Percent confidence interval - upper                     0.157
##   P-value H_0: Robust RMSEA <= 0.050                         0.000
##   P-value H_0: Robust RMSEA >= 0.080                         0.985
##
## Standardized Root Mean Square Residual:
##
##   SRMR                                           0.020       0.020
##
## Parameter Estimates:
##
##   Standard errors                           Robust.sem
##   Information                                 Expected
##   Information saturated (h1) model        Unstructured
##
## Latent Variables:
##                           Estimate  Std.Err  z-value  P(>|z|)   Std.lv  Std.all
##   conspiracy_mentality =~
##     cmq1                     1.000                               0.717    0.717
##     cmq2                     0.963    0.033   29.104    0.000    0.690    0.690
##     cmq3                     0.962    0.035   27.625    0.000    0.690    0.690
##     cmq4                     1.283    0.042   30.556    0.000    0.920    0.920
##     cmq5                     1.272    0.042   30.127    0.000    0.912    0.912
##
## Covariances:
##                    Estimate  Std.Err  z-value  P(>|z|)   Std.lv  Std.all
##  .cmq1 ~~
##    .cmq2              0.266    0.021   12.679    0.000    0.266    0.528
##
## Intercepts:
##                    Estimate  Std.Err  z-value  P(>|z|)   Std.lv  Std.all
##    .cmq1              0.000                               0.000    0.000
##    .cmq2              0.000                               0.000    0.000
##    .cmq3              0.000                               0.000    0.000
##    .cmq4              0.000                               0.000    0.000
##    .cmq5              0.000                               0.000    0.000
##     cnsprcy_mntlty    0.000                               0.000    0.000
##
## Thresholds:
##                    Estimate  Std.Err  z-value  P(>|z|)   Std.lv  Std.all
##     cmq1|t1          -2.298    0.154  -14.929    0.000   -2.298   -2.298
##     cmq1|t2          -1.958    0.113  -17.313    0.000   -1.958   -1.958
##     cmq1|t3          -1.778    0.098  -18.075    0.000   -1.778   -1.778
##     cmq1|t4          -1.546    0.084  -18.386    0.000   -1.546   -1.546
##     cmq1|t5          -1.342    0.075  -17.945    0.000   -1.342   -1.342
##     cmq1|t6          -0.917    0.062  -14.761    0.000   -0.917   -0.917
##     cmq1|t7          -0.588    0.057  -10.383    0.000   -0.588   -0.588
##     cmq1|t8          -0.165    0.053   -3.089    0.002   -0.165   -0.165
##     cmq1|t9           0.229    0.054    4.272    0.000    0.229    0.229
##     cmq2|t1          -2.913    0.312   -9.326    0.000   -2.913   -2.913
[illegible]
```

```
##     cmq2|t2          -2.240    0.145  -15.418    0.000   -2.240   -2.240
##     cmq2|t3          -1.990    0.116  -17.150    0.000   -1.990   -1.990
##     cmq2|t4          -1.824    0.102  -17.935    0.000   -1.824   -1.824
##     cmq2|t5          -1.401    0.077  -18.160    0.000   -1.401   -1.401
##     cmq2|t6          -1.166    0.069  -17.007    0.000   -1.166   -1.166
##     cmq2|t7          -0.717    0.058  -12.288    0.000   -0.717   -0.717
##     cmq2|t8          -0.306    0.054   -5.659    0.000   -0.306   -0.306
##     cmq2|t9           0.122    0.053    2.283    0.022    0.122    0.122
##     cmq2|t10          0.579    0.056   10.250    0.000    0.579    0.579
##     cmq3|t1          -1.757    0.097  -18.151    0.000   -1.757   -1.757
##     cmq3|t2          -1.123    0.067  -16.692    0.000   -1.123   -1.123
##     cmq3|t3          -0.777    0.059  -13.087    0.000   -0.777   -0.777
##     cmq3|t4          -0.406    0.055   -7.422    0.000   -0.406   -0.406
##     cmq3|t5          -0.027    0.053   -0.508    0.612   -0.027   -0.027
##     cmq3|t6           0.282    0.054    5.238    0.000    0.282    0.282
##     cmq3|t7           0.589    0.057   10.415    0.000    0.589    0.589
##     cmq3|t8           0.871    0.061   14.257    0.000    0.871    0.871
##     cmq3|t9           1.148    0.068   16.883    0.000    1.148    1.148
##     cmq3|t10          1.661    0.090   18.351    0.000    1.661    1.661
##     cmq4|t1          -1.626    0.088  -18.388    0.000   -1.626   -1.626
##     cmq4|t2          -0.960    0.063  -15.234    0.000   -0.960   -0.960
##     cmq4|t3          -0.729    0.059  -12.449    0.000   -0.729   -0.729
##     cmq4|t4          -0.406    0.055   -7.422    0.000   -0.406   -0.406
##     cmq4|t5          -0.199    0.053   -3.720    0.000   -0.199   -0.199
##     cmq4|t6           0.363    0.054    6.668    0.000    0.363    0.363
##     cmq4|t7           0.706    0.058   12.127    0.000    0.706    0.706
##     cmq4|t8           0.912    0.062   14.713    0.000    0.912    0.912
##     cmq4|t9           1.311    0.074   17.830    0.000    1.311    1.311
##     cmq4|t10          1.626    0.088   18.388    0.000    1.626    1.626
##     cmq5|t1          -1.697    0.093  -18.277    0.000   -1.697   -1.697
##     cmq5|t2          -1.156    0.068  -16.923    0.000   -1.156   -1.156
##     cmq5|t3          -0.877    0.061  -14.306    0.000   -0.877   -0.877
##     cmq5|t4          -0.615    0.057  -10.794    0.000   -0.615   -0.615
##     cmq5|t5          -0.375    0.055   -6.884    0.000   -0.375   -0.375
##     cmq5|t6          -0.038    0.053   -0.720    0.472   -0.038   -0.038
##     cmq5|t7           0.280    0.054    5.200    0.000    0.280    0.280
##     cmq5|t8           0.535    0.056    9.556    0.000    0.535    0.535
##     cmq5|t9           0.800    0.060   13.374    0.000    0.800    0.800
##     cmq5|t10          1.138    0.068   16.797    0.000    1.138    1.138
##
## Variances:
##                    Estimate  Std.Err  z-value  P(>|z|)   Std.lv  Std.all
##    .cmq1              0.486                               0.486    0.486
##    .cmq2              0.524                               0.524    0.524
##    .cmq3              0.524                               0.524    0.524
##    .cmq4              0.153                               0.153    0.153
##    .cmq5              0.169                               0.169    0.169
##     cnsprcy_mntlty    0.514    0.035   14.892    0.000    1.000    1.000
##
## Scales y*:
##                    Estimate  Std.Err  z-value  P(>|z|)   Std.lv  Std.all
##     cmq1              1.000                               1.000    1.000
##     cmq2              1.000                               1.000    1.000
##     cmq3              1.000                               1.000    1.000
##     cmq4              1.000                               1.000    1.000
##     cmq5              1.000                               1.000    1.000
##
## R-Square:
##                    Estimate
##     cmq1              0.514
##     cmq2              0.476
##     cmq3              0.476
##     cmq4              0.847
##     cmq5              0.831
```

```
# Show the standardized results
cm_loadings <- standardizedsolution(m_conspiracy_mentality)

cm_loadings
```

```
##                   lhs op                rhs est.std    se       z pvalue
## 1  conspiracy_mentality =~               cmq1   0.717 0.024  29.783  0.000
## 2  conspiracy_mentality =~               cmq2   0.690 0.022  31.887  0.000
## 3  conspiracy_mentality =~               cmq3   0.690 0.021  33.318  0.000
## 4  conspiracy_mentality =~               cmq4   0.920 0.010  90.041  0.000
## 5  conspiracy_mentality =~               cmq5   0.912 0.010  93.693  0.000
## 6                  cmq1 ~~               cmq2   0.528 0.027  19.615  0.000
## 7                  cmq1  |                 t1  -2.298 0.154 -14.929  0.000
## 8                  cmq1  |                 t2  -1.958 0.113 -17.313  0.000
## 9                  cmq1  |                 t3  -1.778 0.098 -18.075  0.000
## 10                 cmq1  |                 t4  -1.546 0.084 -18.386  0.000
## 11                 cmq1  |                 t5  -1.342 0.075 -17.945  0.000
## 12                 cmq1  |                 t6  -0.917 0.062 -14.761  0.000
## 13                 cmq1  |                 t7  -0.588 0.057 -10.383  0.000
## 14                 cmq1  |                 t8  -0.165 0.053  -3.089  0.002
## 15                 cmq1  |                 t9   0.229 0.054   4.272  0.000
## 16                 cmq2  |                 t1  -2.913 0.312  -9.326  0.000
## 17                 cmq2  |                 t2  -2.240 0.145 -15.418  0.000
## 18                 cmq2  |                 t3  -1.990 0.116 -17.150  0.000
## 19                 cmq2  |                 t4  -1.824 0.102 -17.935  0.000
## 20                 cmq2  |                 t5  -1.401 0.077 -18.160  0.000
## 21                 cmq2  |                 t6  -1.166 0.069 -17.007  0.000
## 22                 cmq2  |                 t7  -0.717 0.058 -12.288  0.000
## 23                 cmq2  |                 t8  -0.306 0.054  -5.659  0.000
## 24                 cmq2  |                 t9   0.122 0.053   2.283  0.022
## 25                 cmq2  |                t10   0.579 0.056  10.250  0.000
## 26                 cmq3  |                 t1  -1.757 0.097 -18.151  0.000
## 27                 cmq3  |                 t2  -1.123 0.067 -16.692  0.000
## 28                 cmq3  |                 t3  -0.777 0.059 -13.087  0.000
## 29                 cmq3  |                 t4  -0.406 0.055  -7.422  0.000
## 30                 cmq3  |                 t5  -0.027 0.053  -0.508  0.612
## 31                 cmq3  |                 t6   0.282 0.054   5.238  0.000
## 32                 cmq3  |                 t7   0.589 0.057  10.415  0.000
## 33                 cmq3  |                 t8   0.871 0.061  14.257  0.000
## 34                 cmq3  |                 t9   1.148 0.068  16.883  0.000
## 35                 cmq3  |                t10   1.661 0.090  18.351  0.000
## 36                 cmq4  |                 t1  -1.626 0.088 -18.388  0.000
## 37                 cmq4  |                 t2  -0.960 0.063 -15.234  0.000
## 38                 cmq4  |                 t3  -0.729 0.059 -12.449  0.000
## 39                 cmq4  |                 t4  -0.406 0.055  -7.422  0.000
## 40                 cmq4  |                 t5  -0.199 0.053  -3.720  0.000
## 41                 cmq4  |                 t6   0.363 0.054   6.668  0.000
## 42                 cmq4  |                 t7   0.706 0.058  12.127  0.000
## 43                 cmq4  |                 t8   0.912 0.062  14.713  0.000
## 44                 cmq4  |                 t9   1.311 0.074  17.830  0.000
## 45                 cmq4  |                t10   1.626 0.088  18.388  0.000
## 46                 cmq5  |                 t1  -1.697 0.093 -18.277  0.000
## 47                 cmq5  |                 t2  -1.156 0.068 -16.923  0.000
## 48                 cmq5  |                 t3  -0.877 0.061 -14.306  0.000
## 49                 cmq5  |                 t4  -0.615 0.057 -10.794  0.000
## 50                 cmq5  |                 t5  -0.375 0.055  -6.884  0.000
## 51                 cmq5  |                 t6  -0.038 0.053  -0.720  0.472
## 52                 cmq5  |                 t7   0.280 0.054   5.200  0.000
## 53                 cmq5  |                 t8   0.535 0.056   9.556  0.000
## 54                 cmq5  |                 t9   0.800 0.060  13.374  0.000
## 55                 cmq5  |                t10   1.138 0.068  16.797  0.000
## 56                 cmq1 ~~               cmq1   0.486 0.035  14.075  0.000
## 57                 cmq2 ~~               cmq2   0.524 0.030  17.524  0.000
## 58                 cmq3 ~~               cmq3   0.524 0.029  18.352  0.000
## 59                 cmq4 ~~               cmq4   0.153 0.019   8.149  0.000
## 60                 cmq5 ~~               cmq5   0.169 0.018   9.510  0.000
## 61 conspiracy_mentality ~~ conspiracy_mentality   1.000 0.000      NA     NA
## 62                 cmq1 ~*~              cmq1   1.000 0.000      NA     NA
## 63                 cmq2 ~*~              cmq2   1.000 0.000      NA     NA
## 64                 cmq3 ~*~              cmq3   1.000 0.000      NA     NA
## 65                 cmq4 ~*~              cmq4   1.000 0.000      NA     NA
## 66                 cmq5 ~*~              cmq5   1.000 0.000      NA     NA
## 67                 cmq1 ~1                      0.000 0.000      NA     NA
## 68                 cmq2 ~1                      0.000 0.000      NA     NA
## 69                 cmq3 ~1                      0.000 0.000      NA     NA
## 70                 cmq4 ~1                      0.000 0.000      NA     NA
## 71                 cmq5 ~1                      0.000 0.000      NA     NA
## 72 conspiracy_mentality ~1                      0.000 0.000      NA     NA
##    ci.lower ci.upper
## 1     0.670    0.764
## 2     0.648    0.733
## 3     0.649    0.730
## 4     0.900    0.940
## 5     0.893    0.931
## 6     0.475    0.580
## 7    -2.600   -1.997
## 8    -2.179   -1.736
## 9    -1.971   -1.585
## 10   -1.711   -1.381
## 11   -1.489   -1.196
## 12   -1.039   -0.795
## 13   -0.699   -0.477
## 14   -0.270   -0.060
## 15    0.124    0.334
## 16   -3.525   -2.301
## 17   -2.525   -1.955
## 18   -2.217   -1.763
## 19   -2.024   -1.625
## 20   -1.552   -1.250
## 21   -1.300   -1.031
## 22   -0.832   -0.603
## 23   -0.412   -0.200
## 24    0.017    0.226
## 25    0.468    0.689
## 26   -1.947   -1.568
## 27   -1.254   -0.991
## 28   -0.893   -0.660
## 29   -0.513   -0.299
## 30   -0.131    0.077
## 31    0.177    0.388
## 32    0.479    0.700
## 33    0.752    0.991
## 34    1.015    1.281
## 35    1.483    1.838
## 36   -1.799   -1.453
## 37   -1.084   -0.837
## 38   -0.844   -0.614
## 39   -0.513   -0.299
## 40   -0.304   -0.094
## 41    0.256    0.469
## 42    0.592    0.820
## 43    0.790    1.033
## 44    1.167    1.455
## 45    1.453    1.799
## 46   -1.879   -1.515
## 47   -1.290   -1.022
## 48   -0.997   -0.757
## 49   -0.727   -0.503
## 50   -0.482   -0.269
## 51   -0.142    0.066
## 52    0.175    0.386
## 53    0.425    0.645
## 54    0.683    0.917
## 55    1.006    1.271
## 56    0.418    0.554
## 57    0.465    0.582
## 58    0.468    0.580
## 59    0.116    0.190
## 60    0.134    0.204
## 61    1.000    1.000
## 62    1.000    1.000
## 63    1.000    1.000
## 64    1.000    1.000
## 65    1.000    1.000
## 66    1.000    1.000
## 67    0.000    0.000
## 68    0.000    0.000
## 69    0.000    0.000
## 70    0.000    0.000
## 71    0.000    0.000
## 72    0.000    0.000
```

```
# Calculate factor scores
conspiracy_mentality_score <- predict(m_conspiracy_mentality)

# Add the calculated factor scores to the data file
tib <- cbind(tib, conspiracy_mentality_score)
```

Hierarchical Cluster Analysis

Data preparations

```
# Drop observations with na in the variables used for clustering
tib_hca <- tib %>%
  drop_na(trust_vaccines,
          trust_hca,
          format_preference,
          conspiracy_mentality,
          c_vaccine_easy_pre,
          c_vaccine_safe_pre,
          c_vaccine_efficacy_pre,
          c_threat_pre,
          c_altruism_pre,
          seasonal_c_vaccine_probability_pre,
          third_c_vaccine_probability_pre)

# Create z-transformed scores for the variables used for clustering
tib_hca <- tib_hca %>%
  mutate(trust_vaccines_z = as.numeric(scale(trust_vaccines)),
         trust_hca_z = as.numeric(scale(trust_hca)),
         format_preference_z = as.numeric(scale(format_preference)),
         conspiracy_mentality_z = as.numeric(scale(conspiracy_mentality)),
         c_vaccine_easy_pre_z = as.numeric(scale(c_vaccine_easy_pre)),
         c_vaccine_safe_pre_z = as.numeric(scale(c_vaccine_safe_pre)),
         c_vaccine_efficacy_pre_z = as.numeric(scale(c_vaccine_efficacy_pre)),
         c_threat_pre_z = as.numeric(scale(c_threat_pre)),
         c_altruism_pre_z = as.numeric(scale(c_altruism_pre)),
         seasonal_c_vaccine_probability_pre_z = as.numeric(scale(seasonal_c_vaccine_probability_pre)),
         third_c_vaccine_probability_pre_z = as.numeric(scale(third_c_vaccine_probability_pre)))

# Create a new data frame with only the relevant variables for clustering
tib_hca_variables <- tib_hca %>%
  select(trust_vaccines_z,
         trust_hca_z,
         format_preference_z,
         conspiracy_mentality_z,
         c_vaccine_easy_pre_z,
         c_vaccine_safe_pre_z,
         c_vaccine_efficacy_pre_z,
         c_threat_pre_z,
         c_altruism_pre_z,
         seasonal_c_vaccine_probability_pre_z,
         third_c_vaccine_probability_pre_z)
```

Create a distance matrix and conduct the hierarchical cluster analysis

```
# Create a distance matrix with euclidean distances
d <- dist(tib_hca_variables, method = "euclidean")

# Conduct hierarchical clustering using Ward's method
hc_ward <- hclust(d, method = "ward.D2")
```

Plot the dendrogram

```
# Ward dendrogram
plot(hc_ward,
     cex = 0.5,
     hang = -1,
     labels = FALSE,
     xlab = "",
     ylab = "",
     main = "",
     sub = "")
```

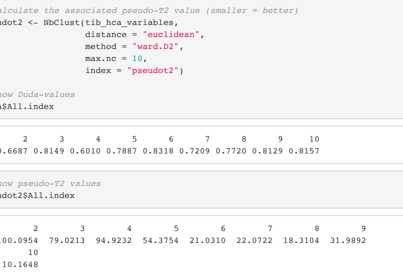

Determine the optimal number of clusters

```
# Calculate the Duda-Hart Je(2)/Je(1) value (bigger = better)
duda <- NbClust(tib_hca_variables,
                distance = "euclidean",
                method = "ward.D2",
                max.nc = 10,
                index = "duda")

# Calculate the associated pseudo-T2 value (smaller = better)
pseudot2 <- NbClust(tib_hca_variables,
                    distance = "euclidean",
                    method = "ward.D2",
                    max.nc = 10,
                    index = "pseudot2")

# Show Duda-values
duda$All.index
```

```
##      2      3      4      5      6      7      8      9     10
## 0.6687 0.8149 0.6010 0.7887 0.8318 0.7209 0.7720 0.8129 0.8157
```

```
# Show pseudo-T2 values
pseudot2$All.index
```

```
##        2        3        4        5        6        7        8        9
## 100.0954  79.0213  94.9232  54.3754  21.0310  22.0722  18.3104  31.9892
##       10
##  10.1648
```

```
# Show best cluster solution
duda$Best.nc
```

```
## Number_clusters     Value_Index
##          6.0000          0.8318
```

Visualize the groups

```
# Ward dendrogram
plot(hc_ward,
     cex = 0.6,
     hang = -1,
     labels = FALSE)

# Show the Ward groups on the dendrogram
rect.hclust(hc_ward,
            k = 6,
            border = 8:14)
```

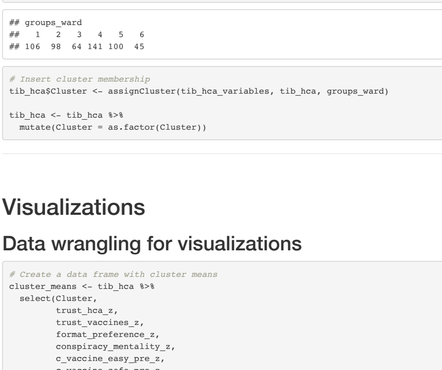

Insert cluster membership into the original data file

```
# Create 6 groups based on the Ward clustering results
groups_ward <- cutree(hc_ward, k = 6)

# Check the group sizes
table(groups_ward)
```

```
## groups_ward
##   1   2   3   4   5   6
## 106  98  64 141 100  45
```

```
# Insert cluster membership
tib_hca$Cluster <- assignCluster(tib_hca_variables, tib_hca, groups_ward)

tib_hca <- tib_hca %>%
  mutate(Cluster = as.factor(Cluster))
```

Visualizations

Data wrangling for visualizations

```
# Create a data frame with cluster means
cluster_means <- tib_hca %>%
  select(Cluster,
         trust_hca_z,
         trust_vaccines_z,
         format_preference_z,
         conspiracy_mentality_z,
         c_vaccine_easy_pre_z,
         c_vaccine_safe_pre_z,
         c_vaccine_efficacy_pre_z,
         c_threat_pre_z,
         c_altruism_pre_z,
         seasonal_c_vaccine_probability_pre_z,
         third_c_vaccine_probability_pre_z) %>%
  group_by(Cluster) %>%
  summarise(trust_hca_z_mean = mean(trust_hca_z),
            trust_vaccines_z_mean = mean(trust_vaccines_z),
            format_preference_z_mean = mean(format_preference_z),
            conspiracy_mentality_z_mean = mean(conspiracy_mentality_z),
            c_vaccine_easy_pre_z_mean = mean(c_vaccine_easy_pre_z),
            c_vaccine_safe_pre_z_mean = mean(c_vaccine_safe_pre_z),
            c_vaccine_efficacy_pre_z_mean = mean(c_vaccine_efficacy_pre_z),
            c_threat_pre_z_mean = mean(c_threat_pre_z),
            c_altruism_pre_z_mean = mean(c_altruism_pre_z),
            seasonal_c_vaccine_probability_pre_z_mean = mean(seasonal_c_vaccine_probability_pre_z),
            third_c_vaccine_probability_pre_z_mean = mean(third_c_vaccine_probability_pre_z))

# Rename the clusters
cluster_means <- cluster_means %>%
  mutate(Cluster = dplyr::recode(Cluster,
                                 "1" = "The Ambivalent",
                                 "2" = "The Vaccination Inclined",
                                 "3" = "The Fearing Skeptic",
                                 "4" = "The Unconvinced",
                                 "5" = "The Vaccination Opponent",
                                 "6" = "The Constrained Critic"),
         Cluster = factor(Cluster, c("The Vaccination Inclined",
                                     "The Ambivalent",
                                     "The Fearing Skeptic",
                                     "The Unconvinced",
                                     "The Constrained Critic",
                                     "The Vaccination Opponent")))
```

Visualize the results with parallel coordinate plots

```
# Import? Times New Roman
windowsFonts(Times = windowsFont("Times New Roman"))

# Create a custom color palette (colorblind friendly)
cbbPalette <- c("#0072B2",
                "#56B4E9",
                "#009E73",
                "#F0E442",
                "#F5635B",
                "#C81E17")

# Plot with cluster means
cluster_means_pcp <- cluster_means %>%
  select(Cluster,
         format_preference_z_mean,
         conspiracy_mentality_z_mean,
         trust_hca_z_mean,
         c_vaccine_easy_pre_z_mean,
         c_altruism_pre_z_mean,
         c_threat_pre_z_mean,
         c_vaccine_efficacy_pre_z_mean,
         c_vaccine_safe_pre_z_mean,
         trust_vaccines_z_mean,
         third_c_vaccine_probability_pre_z_mean,
         seasonal_c_vaccine_probability_pre_z_mean)

cluster_means_pcp <- cluster_means_pcp %>%
  mutate(format_preference_z_mean = format_preference_z_mean * (-1),
         conspiracy_mentality_z_mean = conspiracy_mentality_z_mean * (-1))

ggparcoord(cluster_means_pcp,
           columns = 2:12,
           groupColumn = 1,
           showPoints = FALSE,
           scale = "globalminmax",) +
       theme_minimal() +
       theme(text = element_text(size = 12, family = "Times"),
             panel.grid.major.y = element_blank(),
             legend.title = element_blank()) +
       coord_flip() +
       geom_hline(yintercept = 0,
                  linewidth = 1,
                  color = "darkgrey",
                  linetype = "dashed") +
       geom_line(size = 1.3) +
       scale_colour_manual(values = cbbPalette) +
       scale_y_continuous("Standardized Score") +
       scale_x_discrete("", labels = c("format_preference_z_mean" = "Format Preference*",
                                       "conspiracy_mentality_z_mean" = "Conspiracy Mentality*",
                                       "c_altruism_pre_z_mean" = "Vaccination Altruism",
                                       "c_threat_pre_z_mean" = "Disease Threat",
                                       "c_vaccine_efficacy_pre_z_mean" = "Vaccine Efficacy",
                                       "c_vaccine_safe_pre_z_mean" = "Specific Vaccine Safety",
                                       "c_vaccine_easy_pre_z_mean" = "Vaccination Convenience",
                                       "trust_vaccines_z_mean" = "General Vaccine Safety",
                                       "trust_hca_z_mean" = "Trust in Health Authorities",
                                       "seasonal_c_vaccine_probability_pre_z_mean" = "Seasonal Vaccine",
                                       "third_c_vaccine_probability_pre_z_mean" = "Third Vaccine"))
```

```
## Warning: Using `size` aesthetic for lines was deprecated in ggplot2 3.4.0.
## ℹ Please use `linewidth` instead.
## This warning is displayed once every 8 hours.
## Call `lifecycle::last_lifecycle_warnings()` to see where this warning was
## generated.
```

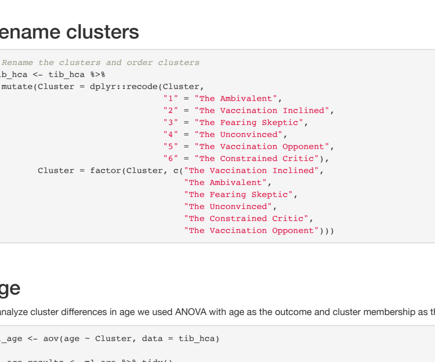

Analysing Group Differences in Background Variables

Rename clusters

```
# Rename the clusters and order clusters
tib_hca <- tib_hca %>%
  mutate(Cluster = dplyr::recode(Cluster,
                                 "1" = "The Ambivalent",
                                 "2" = "The Vaccination Inclined",
                                 "3" = "The Fearing Skeptic",
                                 "4" = "The Unconvinced",
                                 "5" = "The Vaccination Opponent",
                                 "6" = "The Constrained Critic"),
         Cluster = factor(Cluster, c("The Vaccination Inclined",
                                     "The Ambivalent",
                                     "The Fearing Skeptic",
                                     "The Unconvinced",
                                     "The Constrained Critic",
                                     "The Vaccination Opponent")))
```

Age

To analyze cluster differences in age we used ANOVA with age as the outcome and cluster membership as the predictor.

```
m1_age <- aov(age ~ Cluster, data = tib_hca)

m1_age_results <- m1_age %>% tidy()

m1_age_results
```

```
## # A tibble: 2 × 6
##   term         df  sumsq meansq statistic p.value
##   <chr>     <dbl>  <dbl>  <dbl>     <dbl>   <dbl>
## 1 Cluster       5  1627.   325.      2.03  0.0738
## 2 Residuals   453 72755.   161.     NA     NA
```

```
ggplot(m1_age, aes(x = Cluster, y = age, color = Cluster)) +
  geom_boxplot() +
  theme_bw() +
  stat_summary(geom = "crossbar", fun = "mean", color = "red")
```

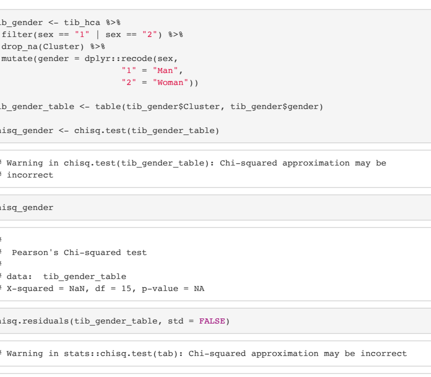

Gender

```
tib_gender <- tib_hca %>%
  filter(sex == "1" | sex == "2") %>%
  drop_na(Cluster) %>%
  mutate(gender = dplyr::recode(sex,
                         "1" = "Man",
                         "2" = "Woman"))

tib_gender_table <- table(tib_gender$Cluster, tib_gender$gender)

chisq_gender <- chisq.test(tib_gender_table)
```

```
## Warning in chisq.test(tib_gender_table): Chi-squared approximation may be
## incorrect
```

```
chisq_gender
```

```
##
##  Pearson's Chi-squared test
##
## data:  tib_gender_table
## X-squared = NaN, df = 15, p-value = NA
```

```
chisq.residuals(tib_gender_table, std = FALSE)
```

```
## Warning in stats::chisq.test(tab): Chi-squared approximation may be incorrect
```

```
##
##                             Man Woman 3 4
##   The Vaccination Inclined -1.21  0.59
##   The Ambivalent            0.57 -0.28
##   The Fearing Skeptic      -2.43  1.18
##   The Unconvinced           0.19 -0.09
##   The Constrained Critic    2.33 -1.13
##   The Vaccination Opponent  0.85 -0.41
```

```
chisq.residuals(tib_gender_table, std = TRUE)
```

```
## Warning in stats::chisq.test(tab): Chi-squared approximation may be incorrect
```

```
##
##                             Man Woman 3 4
##   The Vaccination Inclined -1.49  1.49
##   The Ambivalent            0.70 -0.70
##   The Fearing Skeptic      -2.87  2.87
##   The Unconvinced           0.25 -0.25
##   The Constrained Critic    2.69 -2.69
##   The Vaccination Opponent  1.05 -1.05
```

```
gender_contrib <- round(100*chisq_gender$residuals^2/chisq_gender$statistic, 3)

gender_contrib
```

```
##
##                           Man Woman 3 4
##   The Vaccination Inclined
##   The Ambivalent
##   The Fearing Skeptic
##   The Unconvinced
##   The Constrained Critic
##   The Vaccination Opponent
```

```
ggplot(tib_gender, aes(x = Cluster, fill = gender)) +
  geom_bar(position = "fill") +
  theme_bw() +
  labs(y = "Fraction", fill = "Gender") +
  coord_flip()
```

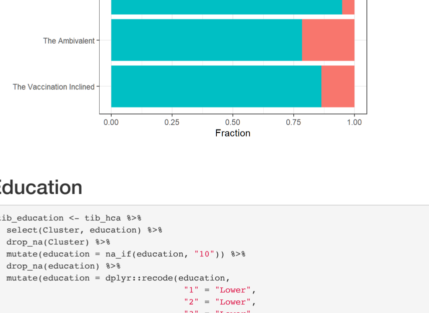

Education

```
tib_education <- tib_hca %>%
  select(Cluster, education) %>%
  drop_na(Cluster) %>%
  mutate(education = na_if(education, "10")) %>%
  drop_na(education) %>%
  mutate(education = dplyr::recode(education,
                                   "1" = "Lower",
                                   "2" = "Lower",
                                   "3" = "Lower",
                                   "4" = "Lower",
                                   "5" = "Lower",
```

```
                                   "6" = "Lower",
                                   "7" = "Higher",
                                   "8" = "Higher",
                                   "9" = "Higher"))


tib_education_table <- table(tib_education$Cluster, tib_education$education)

chisq_education <- chisq.test(tib_education_table)
```

```
## Warning in chisq.test(tib_education_table): Chi-squared approximation may be
## incorrect
```

```
chisq_education
```

```
##
##  Pearson's Chi-squared test
##
## data:  tib_education_table
## X-squared = NaN, df = 10, p-value = NA
```

```
chisq.residuals(tib_education_table, std = FALSE)
```

```
## Warning in stats::chisq.test(tab): Chi-squared approximation may be incorrect
```

```
##
##                           Lower Higher 10
##   The Vaccination Inclined -0.45   0.38
##   The Ambivalent            0.56  -0.48
##   The Fearing Skeptic       0.75  -0.64
##   The Unconvinced          -1.84   1.55
##   The Constrained Critic    1.69  -1.43
##   The Vaccination Opponent  0.32  -0.27
```

```
chisq.residuals(tib_education_table, std = TRUE)
```

```
## Warning in stats::chisq.test(tab): Chi-squared approximation may be incorrect
```

```
##
##                           Lower Higher 10
##   The Vaccination Inclined -0.65   0.65
##   The Ambivalent            0.82  -0.82
##   The Fearing Skeptic       1.05  -1.05
##   The Unconvinced          -2.78   2.78
##   The Constrained Critic    2.31  -2.31
##   The Vaccination Opponent  0.46  -0.46
```

```
education_contrib <- round(100*chisq_education$residuals^2/chisq_education$statistic, 3)

education_contrib
```

```
##
##                           Lower Higher 10
##   The Vaccination Inclined
##   The Ambivalent
##   The Fearing Skeptic
##   The Unconvinced
##   The Constrained Critic
##   The Vaccination Opponent
```

```
ggplot(tib_education, aes(x = Cluster, fill = education)) +
  geom_bar(position = "fill") +
  theme_bw() +
  labs(y = "Fraction", fill = "Level of Education") +
  coord_flip()
```

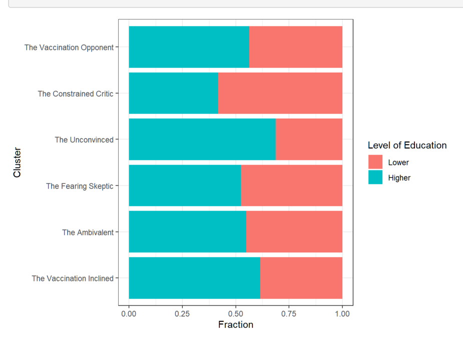

Home Region

```
tib_live <- tib_hca %>%
  select(Cluster, live) %>%
  drop_na(Cluster) %>%
  drop_na(live) %>%
  mutate(live = dplyr::recode(live,
                              "1" = "Uusimaa",
                              "2" = "Southwest Finland",
                              "3" = "Other",
                              "4" = "Other",
                              "5" = "Other",
                              "6" = "Other",
                              "7" = "Other",
                              "8" = "Other",
                              "9" = "Other",
                              "10" = "Other",
                              "11" = "Other",
                              "12" = "Other",
                              "13" = "Other",
                              "14" = "Other",
                              "15" = "Other",
                              "16" = "Other",
                              "17" = "Other",
                              "18" = "Other",
                              "19" = "Other",
                              "20" = "Other",))

tib_live_table <- table(tib_live$Cluster, tib_live$live)

chisq_live <- chisq.test(tib_live_table)

chisq_live
```

```
##
##  Pearson's Chi-squared test
##
## data:  tib_live_table
## X-squared = 18.479, df = 10, p-value = 0.0474
```

```
chisq.residuals(tib_live_table, std = FALSE)
```

```
##
##                           Uusimaa Southwest Finland Other
##   The Vaccination Inclined    1.56              0.09 -1.26
##   The Ambivalent             -1.10              2.29 -0.26
##   The Fearing Skeptic        -0.65              0.95  0.04
##   The Unconvinced             0.70             -1.23  0.05
##   The Constrained Critic     -0.90             -0.71  1.05
##   The Vaccination Opponent   -0.14             -1.28  0.74
```

```
chisq.residuals(tib_live_table, std = TRUE)
```

```
##
##                           Uusimaa Southwest Finland Other
##   The Vaccination Inclined    2.11              0.11 -2.06
##   The Ambivalent             -1.49              2.72 -0.43
##   The Fearing Skeptic        -0.85              1.08  0.07
##   The Unconvinced             0.99             -1.52  0.09
##   The Constrained Critic     -1.15             -0.79  1.61
##   The Vaccination Opponent   -0.19             -1.52  1.20
```

```
live_contrib <- round(100*chisq_live$residuals^2/chisq_live$statistic, 3)

live_contrib
```

```
##
##                           Uusimaa Southwest Finland  Other
##   The Vaccination Inclined  13.247             0.045  8.601
##   The Ambivalent             6.492            28.259  0.378
##   The Fearing Skeptic        2.296             4.848  0.011
##   The Unconvinced            2.664             8.133  0.015
##   The Constrained Critic     4.404             2.717  5.932
##   The Vaccination Opponent   0.110             8.910  2.940
```

```
ggplot(tib_live, aes(x = Cluster, fill = live)) +
  geom_bar(position = "fill") +
  theme_bw() +
  labs(y = "Fraction", fill = "Home Region") +
  coord_flip()
```

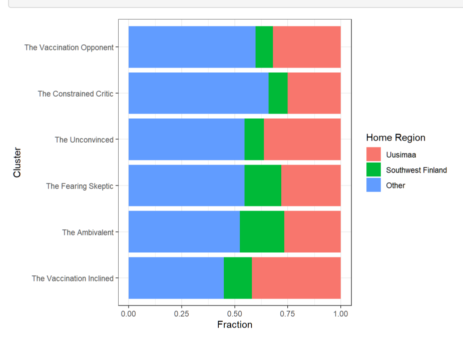

Healthcare Experience

```
tib_hcw <- tib_hca %>%
  select(Cluster, hcw) %>%
  drop_na(Cluster) %>%
  drop_na(hcw) %>%
  mutate(hcw = dplyr::recode(hcw,
                             "1" = "HC Experience",
                             "2" = "No HC Experience"))

tib_hcw_table <- table(tib_hcw$Cluster, tib_hcw$hcw)

chisq_hcw <- chisq.test(tib_hcw_table)

chisq_hcw
```

```
##
##  Pearson's Chi-squared test
##
## data:  tib_hcw_table
## X-squared = 3.8303, df = 5, p-value = 0.5741
```

```
ggplot(tib_hcw, aes(x = Cluster, fill = hcw)) +
  geom_bar(position = "fill") +
  theme_bw() +
  labs(y = "Fraction", fill = "Healthcare Experience") +
  coord_flip()
```

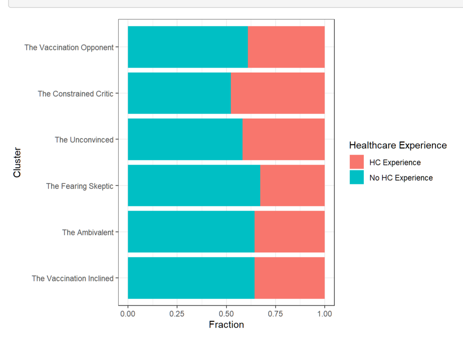

Had COVID-19

```
tib_covid <- tib_hca %>%
  select(Cluster, had_covid) %>%
  drop_na(Cluster) %>%
  drop_na(had_covid) %>%
  mutate(had_covid = dplyr::recode(had_covid,
                                   "1" = "Had COVID",
                                   "2" = "No COVID"))



tib_covid_table <- table(tib_covid$Cluster, tib_covid$had_covid)

chisq_covid <- chisq.test(tib_covid_table, simulate.p.value = TRUE)

chisq_covid
```

```
##
##  Pearson's Chi-squared test with simulated p-value (based on 2000
##  replicates)
##
## data:  tib_covid_table
## X-squared = 16.818, df = NA, p-value = 0.004998
```

```
chisq.residuals(tib_covid_table, std = FALSE)
```

```
## Warning in stats::chisq.test(tab): Chi-squared approximation may be incorrect
```

```
##
##                           Had COVID No COVID
##   The Vaccination Inclined     -1.81     0.60
##   The Ambivalent                0.49    -0.16
##   The Fearing Skeptic          -1.71     0.56
##   The Unconvinced               0.58    -0.19
##   The Constrained Critic        2.89    -0.95
##   The Vaccination Opponent      0.09    -0.03
```

```
chisq.residuals(tib_covid_table, std = TRUE)
```

```
## Warning in stats::chisq.test(tab): Chi-squared approximation may be incorrect
```

```
##
##                           Had COVID No COVID
##   The Vaccination Inclined     -2.11     2.11
##   The Ambivalent                0.58    -0.58
##   The Fearing Skeptic          -1.91     1.91
##   The Unconvinced               0.71    -0.71
##   The Constrained Critic        3.17    -3.17
##   The Vaccination Opponent      0.10    -0.10
```

```
covid_contrib <- round(100*chisq_covid$residuals^2/chisq_covid$statistic, 3)

covid_contrib
```

```
##
##                           Had COVID No COVID
##   The Vaccination Inclined    19.531    2.126
##   The Ambivalent               1.449    0.158
##   The Fearing Skeptic         17.364    1.890
##   The Unconvinced              1.997    0.217
##   The Constrained Critic      49.793    5.421
##   The Vaccination Opponent     0.048    0.005
```

```
ggplot(tib_covid, aes(x = Cluster, fill = had_covid)) +
  geom_bar(position = "fill") +
  theme_bw() +
  labs(y = "Fraction", fill = "Had COVID-19") +
  coord_flip()
```

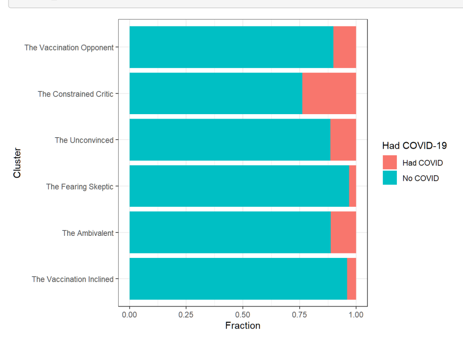

COVID-19 Vaccination Status

```
tib_cvax_status <- tib_hca %>%
  select(Cluster, c_vaccine_taken) %>%
  drop_na(Cluster) %>%
  mutate(cvax_status = dplyr::recode(c_vaccine_taken,
                                     "1" = "Vaccinated",
                                     "2" = "Not Vaccinated"))

tib_cvax_status_table <- table(tib_cvax_status$Cluster, tib_cvax_status$cvax_status)

chisq_cvax_status <- chisq.test(tib_cvax_status_table)

chisq_cvax_status
```

```
##
##  Pearson's Chi-squared test
##
## data:  tib_cvax_status_table
## X-squared = 320.35, df = 5, p-value < 2.2e-16
```

```
chisq.residuals(tib_cvax_status_table, std = FALSE)
```

```
##
##                           Vaccinated Not Vaccinated
##   The Vaccination Inclined      12.23          -8.03
##   The Ambivalent                 2.84          -1.86
##   The Fearing Skeptic           -2.34           1.54
##   The Unconvinced               -5.14           3.38
##   The Constrained Critic        -3.41           2.24
##   The Vaccination Opponent      -4.76           3.13
```

```
chisq.residuals(tib_cvax_status_table, std = TRUE)
```

```
##
##                           Vaccinated Not Vaccinated
##   The Vaccination Inclined      16.13         -16.13
##   The Ambivalent                 3.78          -3.78
##   The Fearing Skeptic           -2.98           2.98
##   The Unconvinced               -7.12           7.12
##   The Constrained Critic        -4.26           4.26
##   The Vaccination Opponent      -6.29           6.29
```

```
cvax_status_contrib <- round(100*chisq_cvax_status$residuals^2/chisq_cvax_status$statistic, 3)

cvax_status_contrib
```

```
##
##                           Vaccinated Not Vaccinated
##   The Vaccination Inclined     46.671         20.140
##   The Ambivalent                2.516          1.086
##   The Fearing Skeptic           1.714          0.740
##   The Unconvinced               8.244          3.557
##   The Constrained Critic        3.633          1.568
##   The Vaccination Opponent      7.078          3.054
```

```
ggplot(tib_cvax_status, aes(x = Cluster, fill = cvax_status)) +
  geom_bar(position = "fill") +
  theme_bw() +
  labs(y = "Fraction", fill = "COVID-19 Vaccination Status") +
  coord_flip()
```

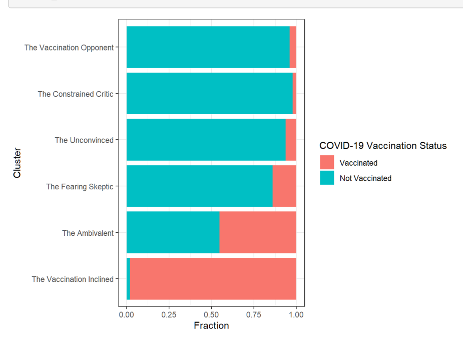

Create a plot for demographics by cluster membership

```
age1 <- ggplot(tib_hca, aes(x = Cluster, y = age, group = Cluster)) +
  geom_boxplot(fill = "#648499") +
  theme_bw() +
  stat_summary(geom = "crossbar", fun = "mean", color = "red") +
  ylab("Age") +
  theme(legend.position = "none") +
  ggtitle("(A).")

tib_gender <- tib_gender %>%
  mutate(Cluster = factor(Cluster, c("The Vaccination Opponent",
                                     "The Constrained Critic",
                                     "The Unconvinced",
                                     "The Fearing Skeptic",
                                     "The Ambivalent",
                                     "The Vaccination Inclined")))

gender1 <- ggplot(tib_gender, aes(x = Cluster, fill = gender)) +
  geom_bar(position = "fill") +
  theme_bw() +
  labs(y = element_blank(),
       fill = "Gender") +
  scale_y_reverse() +
  coord_flip() +
  scale_fill_manual(values = c("#648499", "#BBBBBB"), breaks = c("Man", "Woman")) +
  theme(legend.position = "right", legend.justification = "left", axis.text.x = element_blank(), axis.ticks.x = e
lement_blank()) +
  ggtitle("(B).")

tib_education <- tib_education %>%
  mutate(Cluster = factor(Cluster, c("The Vaccination Opponent",
                                     "The Constrained Critic",
                                     "The Unconvinced",
                                     "The Fearing Skeptic",
                                     "The Ambivalent",
                                     "The Vaccination Inclined")))

education1 <- ggplot(tib_education, aes(x = Cluster, fill = education)) +
  geom_bar(position = "fill") +
  theme_bw() +
  labs(y = element_blank(), fill = "Level of Education") +
  scale_y_reverse() +
  coord_flip() +
  scale_fill_manual(values = c("#648499", "#BBBBBB")) +
  theme(legend.position = "right", legend.justification = "left", axis.text.x = element_blank(), axis.ticks.x = e
lement_blank()) +
  ggtitle("(C).")

tib_live <- tib_live %>%
  mutate(Cluster = factor(Cluster, c("The Vaccination Opponent",
                                     "The Constrained Critic",
                                     "The Unconvinced",
                                     "The Fearing Skeptic",
                                     "The Ambivalent",
                                     "The Vaccination Inclined")))

home1 <- ggplot(tib_live, aes(x = Cluster, fill = live)) +
  geom_bar(position = "fill") +
  theme_bw() +
  labs(y = element_blank(), fill = "Home Region") +
  scale_y_reverse() +
  coord_flip() +
  scale_fill_manual(values = c("#648499", "#BBBBBB", "#E4E4E4")) +
  theme(legend.position = "right", legend.justification = "left", axis.text.x = element_blank(), axis.ticks.x = e
lement_blank()) +
  ggtitle("(D).")

tib_hcw <- tib_hcw %>%
  mutate(Cluster = factor(Cluster, c("The Vaccination Opponent",
                                     "The Constrained Critic",
                                     "The Unconvinced",
                                     "The Fearing Skeptic",
                                     "The Ambivalent",
                                     "The Vaccination Inclined")))

hcw1 <- ggplot(tib_hcw, aes(x = Cluster, fill = hcw)) +
  geom_bar(position = "fill") +
  theme_bw() +
  labs(y = element_blank(), fill = "Healthcare Experience") +
  scale_y_reverse() +
  coord_flip() +
  scale_fill_manual(values = c("#648499", "#BBBBBB")) +
  theme(legend.position = "right", legend.justification = "left", axis.text.x = element_blank(), axis.ticks.x = e
lement_blank()) +
  ggtitle("(E).")

tib_covid <- tib_covid %>%
  mutate(Cluster = factor(Cluster, c("The Vaccination Opponent",
                                     "The Constrained Critic",
                                     "The Unconvinced",
                                     "The Fearing Skeptic",
                                     "The Ambivalent",
                                     "The Vaccination Inclined")))

covid1 <- ggplot(tib_covid, aes(x = Cluster, fill = had_covid)) +
  geom_bar(position = "fill") +
  theme_bw() +
  labs(y = element_blank(), fill = "Had COVID-19") +
  scale_y_reverse() +
  coord_flip() +
  scale_fill_manual(values = c("#648499", "#BBBBBB")) +
  theme(legend.position = "right", legend.justification = "left", axis.text.x = element_blank(), axis.ticks.x = e
lement_blank()) +
  ggtitle("(F).")

tib_cvax_status <- tib_cvax_status %>%
  mutate(Cluster = factor(Cluster, c("The Vaccination Opponent",
                                     "The Constrained Critic",
                                     "The Unconvinced",
                                     "The Fearing Skeptic",
                                     "The Ambivalent",
                                     "The Vaccination Inclined")))

cvax1 <- ggplot(tib_cvax_status, aes(x = Cluster, fill = cvax_status)) +
  geom_bar(position = "fill") +
  theme_bw() +
  labs(y = element_blank(), fill = "COVID-19 Vaccination Status") +
  scale_y_reverse() +
  coord_flip() +
  scale_fill_manual(values = c("#648499", "#BBBBBB")) +
  theme(legend.position = "right", legend.justification = "left", axis.text.x = element_blank(), axis.ticks.x = e
lement_blank()) +
  ggtitle("(G).")

age1 / (gender1 | education1) / (home1 | hcw1) / (covid1 | cvax1)
```

```
## Warning: Removed 95 rows containing non-finite values (`stat_boxplot()`).
```

```
## Warning: Removed 95 rows containing non-finite values (`stat_summary()`).
```

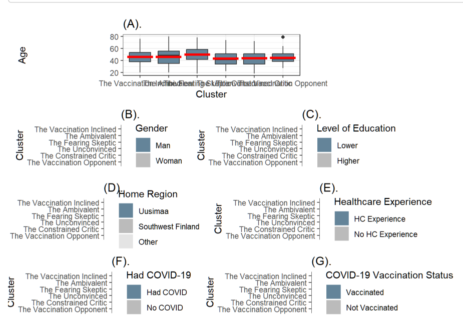

Descriptives

Means and SDs for unstandardized scores

```
tib_means <- tib_hca %>%
  group_by(Cluster) %>%
  get_summary_stats(c(seasonal_c_vaccine_probability_pre,
                      third_c_vaccine_probability_pre,
                      format_preference,
                      conspiracy_mentality,
                      c_altruism_pre,
                      c_threat_pre,
                      c_vaccine_efficacy_pre,
                      c_vaccine_safe_pre,
                      c_vaccine_easy_pre,
                      trust_vaccines,
                      trust_hca),
                    type = "mean_sd") %>%
  ungroup() %>%
  mutate(mean = round(mean, digits = 2),
         sd = round(sd, digits = 2))

kable(tib_means, "simple")
```

Cluster	variable	n	mean	sd
The Vaccination Inclined	seasonal_c_vaccine_probability_pre	98	67.40	14.36
The Vaccination Inclined	third_c_vaccine_probability_pre	98	78.58	17.06
The Vaccination Inclined	format_preference	98	-0.33	0.29
The Vaccination Inclined	conspiracy_mentality	98	-0.65	0.49
The Vaccination Inclined	c_altruism_pre	98	91.00	10.32
The Vaccination Inclined	c_threat_pre	98	55.88	26.27
The Vaccination Inclined	c_vaccine_efficacy_pre	98	75.97	13.04
The Vaccination Inclined	c_vaccine_safe_pre	98	81.47	12.60
The Vaccination Inclined	c_vaccine_easy_pre	98	87.09	14.18
The Vaccination Inclined	trust_vaccines	98	5.52	1.05
The Vaccination Inclined	trust_hca	98	1.22	0.47
The Ambivalent	seasonal_c_vaccine_probability_pre	106	30.93	22.71
The Ambivalent	third_c_vaccine_probability_pre	106	27.88	24.24
The Ambivalent	format_preference	106	-0.07	0.32
The Ambivalent	conspiracy_mentality	106	-0.36	0.51
The Ambivalent	c_altruism_pre	106	46.05	25.97
The Ambivalent	c_threat_pre	106	39.00	24.16
The Ambivalent	c_vaccine_efficacy_pre	106	45.28	20.46
The Ambivalent	c_vaccine_safe_pre	106	47.65	18.69
The Ambivalent	c_vaccine_easy_pre	106	76.95	26.72
The Ambivalent	trust_vaccines	106	3.44	1.42
The Ambivalent	trust_hca	106	0.50	0.42
The Fearing Skeptic	seasonal_c_vaccine_probability_pre	64	9.97	17.41
The Fearing Skeptic	third_c_vaccine_probability_pre	64	2.88	5.38
The Fearing Skeptic	format_preference	64	0.17	0.32
The Fearing Skeptic	conspiracy_mentality	64	0.01	0.67
The Fearing Skeptic	c_altruism_pre	64	15.92	20.89
The Fearing Skeptic	c_threat_pre	64	61.44	24.96
The Fearing Skeptic	c_vaccine_efficacy_pre	64	20.25	22.87
The Fearing Skeptic	c_vaccine_safe_pre	64	12.84	12.89
The Fearing Skeptic	c_vaccine_easy_pre	64	94.86	8.62
The Fearing Skeptic	trust_vaccines	64	1.61	1.00
The Fearing Skeptic	trust_hca	64	-0.14	0.53
The Unconvinced	seasonal_c_vaccine_probability_pre	141	3.10	6.77
The Unconvinced	third_c_vaccine_probability_pre	141	1.19	3.78
The Unconvinced	format_preference	141	-0.16	0.29
The Unconvinced	conspiracy_mentality	141	0.08	0.44
The Unconvinced	c_altruism_pre	141	5.62	9.55
The Unconvinced	c_threat_pre	141	12.53	12.14
The Unconvinced	c_vaccine_efficacy_pre	141	10.01	12.92
The Unconvinced	c_vaccine_safe_pre	141	12.17	13.81
The Unconvinced	c_vaccine_easy_pre	141	93.00	10.12
The Unconvinced	trust_vaccines	141	1.41	0.69
The Unconvinced	trust_hca	141	-0.21	0.44
The Constrained Critic	seasonal_c_vaccine_probability_pre	45	0.96	2.01
The Constrained Critic	third_c_vaccine_probability_pre	45	0.31	0.76
The Constrained Critic	format_preference	45	0.22	0.38
The Constrained Critic	conspiracy_mentality	45	0.57	0.48
The Constrained Critic	c_altruism_pre	45	3.42	8.66
The Constrained Critic	c_threat_pre	45	12.47	18.99
The Constrained Critic	c_vaccine_efficacy_pre	45	5.60	15.67
The Constrained Critic	c_vaccine_safe_pre	45	5.20	15.24
The Constrained Critic	c_vaccine_easy_pre	45	52.11	8.58
The Constrained Critic	trust_vaccines	45	1.18	0.44
The Constrained Critic	trust_hca	45	-0.70	0.44
The Vaccination Opponent	seasonal_c_vaccine_probability_pre	100	0.66	2.46
The Vaccination Opponent	third_c_vaccine_probability_pre	100	0.89	6.80
The Vaccination Opponent	format_preference	100	0.43	0.32
The Vaccination Opponent	conspiracy_mentality	100	0.63	0.58
The Vaccination Opponent	c_altruism_pre	100	0.92	2.98
The Vaccination Opponent	c_threat_pre	100	2.66	5.50
The Vaccination Opponent	c_vaccine_efficacy_pre	100	3.13	5.97
The Vaccination Opponent	c_vaccine_safe_pre	100	2.23	5.57
The Vaccination Opponent	c_vaccine_easy_pre	100	99.17	1.94
The Vaccination Opponent	trust_vaccines	100	1.00	0.00
The Vaccination Opponent	trust_hca	100	-0.87	0.39

Means and SDs for standardized scores

```r
tib_z_means <- tib_hca %>%
  group_by(Cluster) %>%
  get_summary_stats(c(seasonal_c_vaccine_probability_pre_z,
                      third_c_vaccine_probability_pre_z,
                      format_preference_z,
                      conspiracy_mentality_z,
                      c_altruism_pre_z,
                      c_threat_pre_z,
                      c_vaccine_efficacy_pre_z,
                      c_vaccine_safe_pre_z,
                      c_vaccine_easy_pre_z,
                      trust_vaccines_z,
                      trust_hca_z),
                    type = "mean_sd") %>%
  ungroup() %>%
  mutate(mean = round(mean, digits = 2),
         sd = round(sd, digits = 2))

kable(tib_z_means, "simple")
```

Cluster	variable	n	mean	sd
The Vaccination Inclined	seasonal_c_vaccine_probability_pre_z	98	1.69	0.51
The Vaccination Inclined	third_c_vaccine_probability_pre_z	98	1.83	0.53
The Vaccination Inclined	format_preference_z	98	-0.83	0.72
The Vaccination Inclined	conspiracy_mentality_z	98	-0.94	0.72
The Vaccination Inclined	c_altruism_pre_z	98	1.72	0.28
The Vaccination Inclined	c_threat_pre_z	98	0.91	0.90
The Vaccination Inclined	c_vaccine_efficacy_pre_z	98	1.56	0.42
The Vaccination Inclined	c_vaccine_safe_pre_z	98	1.65	0.40
The Vaccination Inclined	c_vaccine_easy_pre_z	98	0.01	0.73
The Vaccination Inclined	trust_vaccines_z	98	1.62	0.56
The Vaccination Inclined	trust_hca_z	98	1.43	0.57
The Ambivalent	seasonal_c_vaccine_probability_pre_z	106	0.39	0.81
The Ambivalent	third_c_vaccine_probability_pre_z	106	0.24	0.76
The Ambivalent	format_preference_z	106	-0.18	0.79
The Ambivalent	conspiracy_mentality_z	106	-0.52	0.75
The Ambivalent	c_altruism_pre_z	106	0.48	0.72
The Ambivalent	c_threat_pre_z	106	0.34	0.82
The Ambivalent	c_vaccine_efficacy_pre_z	106	0.56	0.67
The Ambivalent	c_vaccine_safe_pre_z	106	0.59	0.59
The Ambivalent	c_vaccine_easy_pre_z	106	-0.51	1.38
The Ambivalent	trust_vaccines_z	106	0.52	0.75
The Ambivalent	trust_hca_z	106	0.57	0.50
The Fearing Skeptic	seasonal_c_vaccine_probability_pre_z	64	-0.36	0.62
The Fearing Skeptic	third_c_vaccine_probability_pre_z	64	-0.54	0.17
The Fearing Skeptic	format_preference_z	64	0.41	0.79
The Fearing Skeptic	conspiracy_mentality_z	64	0.01	0.98
The Fearing Skeptic	c_altruism_pre_z	64	-0.35	0.58
The Fearing Skeptic	c_threat_pre_z	64	1.10	0.85
The Fearing Skeptic	c_vaccine_efficacy_pre_z	64	-0.25	0.74
The Fearing Skeptic	c_vaccine_safe_pre_z	64	-0.50	0.40
The Fearing Skeptic	c_vaccine_easy_pre_z	64	0.41	0.45
The Fearing Skeptic	trust_vaccines_z	64	-0.45	0.53
The Fearing Skeptic	trust_hca_z	64	-0.20	0.63
The Unconvinced	seasonal_c_vaccine_probability_pre_z	141	-0.60	0.24
The Unconvinced	third_c_vaccine_probability_pre_z	141	-0.59	0.12
The Unconvinced	format_preference_z	141	-0.40	0.70
The Unconvinced	conspiracy_mentality_z	141	0.12	0.64
The Unconvinced	c_altruism_pre_z	141	-0.63	0.26
The Unconvinced	c_threat_pre_z	141	-0.57	0.41
The Unconvinced	c_vaccine_efficacy_pre_z	141	-0.59	0.42
The Unconvinced	c_vaccine_safe_pre_z	141	-0.52	0.43
The Unconvinced	c_vaccine_easy_pre_z	141	0.32	0.52
The Unconvinced	trust_vaccines_z	141	-0.55	0.36
The Unconvinced	trust_hca_z	141	-0.29	0.52
The Constrained Critic	seasonal_c_vaccine_probability_pre_z	45	-0.68	0.07
The Constrained Critic	third_c_vaccine_probability_pre_z	45	-0.62	0.02
The Constrained Critic	format_preference_z	45	0.55	0.93
The Constrained Critic	conspiracy_mentality_z	45	0.83	0.70
The Constrained Critic	c_altruism_pre_z	45	-0.69	0.24
The Constrained Critic	c_threat_pre_z	45	-0.57	0.65
The Constrained Critic	c_vaccine_efficacy_pre_z	45	-0.73	0.51
The Constrained Critic	c_vaccine_safe_pre_z	45	-0.74	0.48
The Constrained Critic	c_vaccine_easy_pre_z	45	-1.80	0.44
The Constrained Critic	trust_vaccines_z	45	-0.68	0.23
The Constrained Critic	trust_hca_z	45	-0.88	0.53
The Vaccination Opponent	seasonal_c_vaccine_probability_pre_z	100	-0.69	0.09
The Vaccination Opponent	third_c_vaccine_probability_pre_z	100	-0.60	0.21
The Vaccination Opponent	format_preference_z	100	1.05	0.80
The Vaccination Opponent	conspiracy_mentality_z	100	0.93	0.84
The Vaccination Opponent	c_altruism_pre_z	100	-0.76	0.08
The Vaccination Opponent	c_threat_pre_z	100	-0.90	0.19
The Vaccination Opponent	c_vaccine_efficacy_pre_z	100	-0.81	0.19
The Vaccination Opponent	c_vaccine_safe_pre_z	100	-0.84	0.17
The Vaccination Opponent	c_vaccine_easy_pre_z	100	0.64	0.10
The Vaccination Opponent	trust_vaccines_z	100	-0.77	0.00
The Vaccination Opponent	trust_hca_z	100	-1.07	0.47

Demographic variable counts and frequencies by cluster

```r
tib_n <- tib_hca %>%
  select(Cluster,
         age,
         sex,
         education,
         live,
         hcw,
         had_covid,
         c_vaccine_taken,
         c_vaccination_experience) %>%
  mutate(age = case_when(age < 30 ~ "1829",
                         age < 40 ~ "3039",
                         age < 50 ~ "4049",
                         age < 60 ~ "5059",
                         age < 70 ~ "6069",
                         age < 80 ~ "7079",
                         age < 90 ~ "8089",
                         age < 100 ~ "9099",
                         age >= 100 ~ "100"),
         sex = recode_factor(sex,
                             "1" = "Male",
                             "2" = "Female",
                             "3" = "Other",
                             "4" = "DWS"),
         education = recode_factor(education,
                                   "1" = "Lower",
                                   "2" = "Lower",
                                   "3" = "Lower",
                                   "4" = "Lower",
                                   "5" = "Lower",
                                   "6" = "Lower",
                                   "7" = "Higher",
                                   "8" = "Higher",
                                   "9" = "Higher",
                                   "10" = "Other"),
         live = recode_factor(live,
                              "1" = "Uusimaa",
                              "2" = "Southwest",
                              "3" = "Other",
                              "4" = "Other",
                              "5" = "Other",
                              "6" = "Other",
                              "7" = "Other",
                              "8" = "Other",
                              "9" = "Other",
                              "10" = "Other",
                              "11" = "Other",
                              "12" = "Other",
                              "13" = "Other",
                              "14" = "Other",
                              "15" = "Other",
                              "16" = "Other",
                              "17" = "Other",
                              "18" = "Other",
                              "19" = "Other",
                              "20" = "Other"),
         hcw = recode_factor(hcw,
                             "1" = "Yes",
                             "2" = "No"),
         had_covid = recode_factor(had_covid,
                                   "1" = "Yes",
                                   "2" = "No"),
         c_vaccine_taken = recode_factor(c_vaccine_taken,
                                         "1" = "Yes",
                                         "2" = "No"),
         c_vaccination_experience = recode_factor(c_vaccination_experience,
                                                  "1" = "VeryNegative",
                                                  "2" = "SomeNegative",
                                                  "3" = "Neutral",
                                                  "4" = "SomePositive",
                                                  "5" = "VeryPositive")) %>%
  pivot_longer(cols = c(age,
                        sex,
                        education,
                        live,
                        hcw,
                        had_covid,
                        c_vaccine_taken,
                        c_vaccination_experience),
               names_to = "variable",
               values_to = "value")

tib_n %>%
  group_by(Cluster,
           variable,
           value) %>%
  summarise(n = n()) %>%
  mutate(freq = (n / sum(n)) * 100,
         freq = round(freq, digits = 1)) %>%
  ungroup() %>%
  kable("simple")
```

```
## `summarise()` has grouped output by 'Cluster', 'variable'. You can override
## using the `.groups` argument.
```

Cluster	variable	value	n	freq
The Vaccination Inclined	age	1829	13	13.3
The Vaccination Inclined	age	3039	13	13.3
The Vaccination Inclined	age	4049	29	29.6
The Vaccination Inclined	age	5059	22	22.4
The Vaccination Inclined	age	6069	11	11.2
The Vaccination Inclined	age	7079	3	3.1
The Vaccination Inclined	age	NA	7	7.1
The Vaccination Inclined	c_vaccination_experience	Neutral	31	31.6
The Vaccination Inclined	c_vaccination_experience	SomeNegative	1	1.0
The Vaccination Inclined	c_vaccination_experience	SomePositive	37	37.8
The Vaccination Inclined	c_vaccination_experience	VeryPositive	27	27.6
The Vaccination Inclined	c_vaccination_experience	NA	2	2.0
The Vaccination Inclined	c_vaccine_taken	No	2	2.0
The Vaccination Inclined	c_vaccine_taken	Yes	96	98.0
The Vaccination Inclined	education	Higher	59	60.2
The Vaccination Inclined	education	Lower	37	37.8
The Vaccination Inclined	education	Other	2	2.0
The Vaccination Inclined	had_covid	No	94	95.9
The Vaccination Inclined	had_covid	Yes	4	4.1
The Vaccination Inclined	hcw	No	63	64.3
The Vaccination Inclined	hcw	Yes	35	35.7
The Vaccination Inclined	live	Other	44	44.9
The Vaccination Inclined	live	Southwest	13	13.3
The Vaccination Inclined	live	Uusimaa	41	41.8
The Vaccination Inclined	sex	DWS	1	1.0
The Vaccination Inclined	sex	Female	82	83.7
The Vaccination Inclined	sex	Male	13	13.3
The Vaccination Inclined	sex	Other	2	2.0
The Ambivalent	age	1829	10	9.4
The Ambivalent	age	3039	20	18.9
The Ambivalent	age	4049	19	17.9
The Ambivalent	age	5059	22	20.8
The Ambivalent	age	6069	12	11.3
The Ambivalent	age	7079	4	3.8
The Ambivalent	age	8089	1	0.9
The Ambivalent	age	NA	18	17.0
The Ambivalent	c_vaccination_experience	Neutral	24	22.6
The Ambivalent	c_vaccination_experience	SomeNegative	10	9.4
The Ambivalent	c_vaccination_experience	SomePositive	8	7.5
The Ambivalent	c_vaccination_experience	VeryNegative	5	4.7
The Ambivalent	c_vaccination_experience	VeryPositive	1	0.9
The Ambivalent	c_vaccination_experience	NA	58	54.7
The Ambivalent	c_vaccine_taken	No	58	54.7
The Ambivalent	c_vaccine_taken	Yes	48	45.3
The Ambivalent	education	Higher	56	52.8
The Ambivalent	education	Lower	46	43.4
The Ambivalent	education	Other	4	3.8
The Ambivalent	had_covid	No	94	88.7
The Ambivalent	had_covid	Yes	12	11.3
The Ambivalent	hcw	No	67	63.2
The Ambivalent	hcw	Yes	37	34.9
The Ambivalent	hcw	NA	2	1.9
The Ambivalent	live	Other	55	51.9
The Ambivalent	live	Southwest	22	20.8
The Ambivalent	live	Uusimaa	28	26.4
The Ambivalent	live	NA	1	0.9
The Ambivalent	sex	DWS	4	3.8
The Ambivalent	sex	Female	80	75.5
The Ambivalent	sex	Male	22	20.8
The Fearing Skeptic	age	1829	4	6.2
The Fearing Skeptic	age	3039	8	12.5
The Fearing Skeptic	age	4049	13	20.3
The Fearing Skeptic	age	5059	14	21.9
The Fearing Skeptic	age	6069	8	12.5
The Fearing Skeptic	age	7079	5	7.8
The Fearing Skeptic	age	NA	12	18.8
The Fearing Skeptic	c_vaccination_experience	Neutral	6	9.4
The Fearing Skeptic	c_vaccination_experience	SomeNegative	1	1.6
The Fearing Skeptic	c_vaccination_experience	VeryNegative	1	1.6
The Fearing Skeptic	c_vaccination_experience	VeryPositive	1	1.6
The Fearing Skeptic	c_vaccination_experience	NA	55	85.9
The Fearing Skeptic	c_vaccine_taken	No	55	85.9
The Fearing Skeptic	c_vaccine_taken	Yes	9	14.1
The Fearing Skeptic	education	Higher	33	51.6
The Fearing Skeptic	education	Lower	30	46.9
The Fearing Skeptic	education	Other	1	1.6
The Fearing Skeptic	had_covid	No	62	96.9
The Fearing Skeptic	had_covid	Yes	2	3.1
The Fearing Skeptic	hcw	No	43	67.2
The Fearing Skeptic	hcw	Yes	21	32.8
The Fearing Skeptic	live	Other	35	54.7
The Fearing Skeptic	live	Southwest	11	17.2
The Fearing Skeptic	live	Uusimaa	18	28.1
The Fearing Skeptic	sex	DWS	3	4.7
The Fearing Skeptic	sex	Female	55	85.9
The Fearing Skeptic	sex	Male	3	4.7
The Fearing Skeptic	sex	Other	2	3.1
The Fearing Skeptic	sex	NA	1	1.6
The Unconvinced	age	1829	13	9.2
The Unconvinced	age	3039	32	22.7
The Unconvinced	age	4049	34	24.1
The Unconvinced	age	5059	20	14.2
The Unconvinced	age	6069	8	5.7
The Unconvinced	age	7079	5	3.5
The Unconvinced	age	NA	29	20.6
The Unconvinced	c_vaccination_experience	Neutral	2	1.4
The Unconvinced	c_vaccination_experience	SomeNegative	4	2.8
The Unconvinced	c_vaccination_experience	VeryNegative	2	1.4
The Unconvinced	c_vaccination_experience	VeryPositive	1	0.7
The Unconvinced	c_vaccination_experience	NA	132	93.6
The Unconvinced	c_vaccine_taken	No	132	93.6
The Unconvinced	c_vaccine_taken	Yes	9	6.4
The Unconvinced	education	Higher	94	66.7
The Unconvinced	education	Lower	43	30.5
The Unconvinced	education	Other	4	2.8
The Unconvinced	had_covid	No	125	88.7
The Unconvinced	had_covid	Yes	16	11.3
The Unconvinced	hcw	No	82	58.2
The Unconvinced	hcw	Yes	59	41.8
The Unconvinced	live	Other	77	54.6
The Unconvinced	live	Southwest	13	9.2
The Unconvinced	live	Uusimaa	51	36.2
The Unconvinced	sex	DWS	9	6.4
The Unconvinced	sex	Female	105	74.5
The Unconvinced	sex	Male	26	18.4
The Unconvinced	sex	NA	1	0.7
The Constrained Critic	age	1829	5	11.1
The Constrained Critic	age	3039	10	22.2
The Constrained Critic	age	4049	8	17.8
The Constrained Critic	age	5059	7	15.6
The Constrained Critic	age	6069	4	8.9
The Constrained Critic	age	7079	1	2.2
The Constrained Critic	age	NA	10	22.2
The Constrained Critic	c_vaccination_experience	SomeNegative	1	2.2
The Constrained Critic	c_vaccination_experience	NA	44	97.8
The Constrained Critic	c_vaccine_taken	No	44	97.8
The Constrained Critic	c_vaccine_taken	Yes	1	2.2
The Constrained Critic	education	Higher	18	40.0
The Constrained Critic	education	Lower	25	55.6
The Constrained Critic	education	Other	2	4.4
The Constrained Critic	had_covid	No	32	71.1
The Constrained Critic	had_covid	Yes	10	22.2
The Constrained Critic	had_covid	NA	3	6.7
The Constrained Critic	hcw	No	23	51.1
The Constrained Critic	hcw	Yes	21	46.7
The Constrained Critic	hcw	NA	1	2.2
The Constrained Critic	live	Other	29	64.4
The Constrained Critic	live	Southwest	4	8.9
The Constrained Critic	live	Uusimaa	11	24.4
The Constrained Critic	live	NA	1	2.2
The Constrained Critic	sex	DWS	8	17.8
The Constrained Critic	sex	Female	23	51.1
The Constrained Critic	sex	Male	13	28.9
The Constrained Critic	sex	Other	1	2.2
The Vaccination Opponent	age	1829	4	4.0
The Vaccination Opponent	age	3039	20	20.0
The Vaccination Opponent	age	4049	31	31.0
The Vaccination Opponent	age	5059	21	21.0
The Vaccination Opponent	age	6069	4	4.0
The Vaccination Opponent	age	7079	1	1.0
The Vaccination Opponent	age	NA	19	19.0
The Vaccination Opponent	c_vaccination_experience	Neutral	2	2.0
The Vaccination Opponent	c_vaccination_experience	VeryNegative	2	2.0
The Vaccination Opponent	c_vaccination_experience	NA	96	96.0
The Vaccination Opponent	c_vaccine_taken	No	96	96.0
The Vaccination Opponent	c_vaccine_taken	Yes	4	4.0
The Vaccination Opponent	education	Higher	53	53.0
The Vaccination Opponent	education	Lower	41	41.0
The Vaccination Opponent	education	Other	4	4.0
The Vaccination Opponent	education	NA	2	2.0
The Vaccination Opponent	had_covid	No	89	89.0
The Vaccination Opponent	had_covid	Yes	10	10.0
The Vaccination Opponent	had_covid	NA	1	1.0
The Vaccination Opponent	hcw	No	61	61.0
The Vaccination Opponent	hcw	Yes	39	39.0
The Vaccination Opponent	live	Other	58	58.0
The Vaccination Opponent	live	Southwest	8	8.0
The Vaccination Opponent	live	Uusimaa	31	31.0
The Vaccination Opponent	live	NA	3	3.0
The Vaccination Opponent	sex	DWS	2	2.0
The Vaccination Opponent	sex	Female	74	74.0
The Vaccination Opponent	sex	Male	22	22.0
The Vaccination Opponent	sex	Other	1	1.0
The Vaccination Opponent	sex	NA	1	1.0

Demographic variable counts and frequencies for whole sample

```r
tib_n %>%
  group_by(variable,
           value) %>%
  summarise(n = n()) %>%
  mutate(freq = (n / sum(n)) * 100,
         freq = round(freq, digits = 1)) %>%
  ungroup() %>%
  kable("simple")
```

```
## `summarise()` has grouped output by 'variable'. You can override using the
## `.groups` argument.
```

variable	value	n	freq
age	1829	49	8.8
age	3039	103	18.6
age	4049	134	24.2
age	5059	106	19.1
age	6069	47	8.5
age	7079	19	3.4
age	8089	1	0.2
age	NA	95	17.1
c_vaccination_experience	Neutral	65	11.7
c_vaccination_experience	SomeNegative	17	3.1
c_vaccination_experience	SomePositive	45	8.1
c_vaccination_experience	VeryNegative	10	1.8
c_vaccination_experience	VeryPositive	30	5.4
c_vaccination_experience	NA	387	69.9
c_vaccine_taken	No	387	69.9
c_vaccine_taken	Yes	167	30.1
education	Higher	313	56.5
education	Lower	222	40.1
education	Other	17	3.1
education	NA	2	0.4
had_covid	No	496	89.5
had_covid	Yes	54	9.7
had_covid	NA	4	0.7
hcw	No	339	61.2
hcw	Yes	212	38.3
hcw	NA	3	0.5
live	Other	298	53.8
live	Southwest	71	12.8
live	Uusimaa	180	32.5
live	NA	5	0.9
sex	DWS	27	4.9
sex	Female	419	75.6
sex	Male	99	17.9
sex	Other	6	1.1
sex	NA	3	0.5
