# Supplementary material for: COVID-19 and influenza vaccine-hesitancy subgroups
Source: PLoS One. 2024 Jul 30;19(7):e0308159. doi: 10.1371/journal.pone.0308159 (PMC11288446; doi:10.1371/journal.pone.0308159)
Supplement: S3 File — (PDF) [file pone.0308159.s003.pdf]

Inluenza2021 Sample Analyses

Preparations

Load packages

```r
library(tidyverse)
```

```
## ── Attaching core tidyverse packages ──────────────────────── tidyverse 2.0.0 ──
## ✔ dplyr     1.1.2     ✔ readr     2.1.4
## ✔ forcats   1.0.0     ✔ stringr   1.5.0
## ✔ ggplot2   3.4.2     ✔ tibble    3.2.1
## ✔ lubridate 1.9.2     ✔ tidyr     1.3.0
## ✔ purrr     1.0.1
## ── Conflicts ────────────────────────────────────────── tidyverse_conflicts() ──
## ✖ dplyr::filter() masks stats::filter()
## ✖ dplyr::lag()    masks stats::lag()
## ℹ Use the conflicted package (<http://conflicted.r-lib.org/>) to force all conflicts to become errors
```

```r
library(lavaan)
```

```
## This is lavaan 0.6-15
## lavaan is FREE software! Please report any bugs.
```

```r
library(NbClust)
library(RcmdrMisc)
```

```
## Loading required package: car
## Loading required package: carData
##
## Attaching package: 'car'
##
## The following object is masked from 'package:dplyr':
##
##     recode
##
## The following object is masked from 'package:purrr':
##
##     some
##
## Loading required package: sandwich
```

```r
library(GGally)
```

```
## Registered S3 method overwritten by 'GGally':
##   method from
##   +.gg   ggplot2
```

```r
library(broom)
library(questionr)
library(patchwork)
library(rstatix)
```

```
##
## Attaching package: 'rstatix'
##
## The following object is masked from 'package:stats':
##
##     filter
```

```r
library(kableExtra)
```

```
##
## Attaching package: 'kableExtra'
##
## The following object is masked from 'package:dplyr':
##
##     group_rows
```

Import data

```r
tib <- read.csv2("./data/processed_data/Influenza2021.csv") %>%
  tibble()
```

Factor nominal variables

```r
tib <- tib %>%
  mutate(across(c(sex,
                  education,
                  live,
                  hcw,
                  subject,
                  c_vaccine_taken,
                  c_vaccine,
                  c_vaccination_experience,
                  had_covid,
                  i_vaccine_taken,
                  i_vaccination_experience),
                factor))
```

Confirmatory Factor Analyses

Format Preference

```r
# Create the model
format_preference <- "format_preference =~ abq1_rev + abq2 + abq3 + abq4 + abq5 + abq6_rev
                                          abq1_rev ~~ abq6_rev"

# Conduct the CFA
m_format_preference <- cfa(format_preference,
                           estimator = "WLSMV",
                           missing = "pairwise",
                           ordered = TRUE,
                           data = tib)

# Show the results
summary(m_format_preference,
        fit.measures = TRUE,
        standardized = TRUE,
        rsquare = TRUE)
```

```
## lavaan 0.6.15 ended normally after 28 iterations
##
##   Estimator                                       DWLS
##   Optimization method                           NLMINB
##   Number of model parameters                        43
##
##   Number of observations                           540
##   Number of missing patterns                         7
##
## Model Test User Model:
##                                               Standard      Scaled
##   Test Statistic                                31.636     108.779
##   Degrees of freedom                                 8           8
##   P-value (Chi-square)                           0.000       0.000
##   Scaling correction factor                                  0.294
##   Shift parameter                                            1.292
##     simple second-order correction
##
## Model Test Baseline Model:
##
##   Test statistic                             16511.877    7939.757
##   Degrees of freedom                                15          15
##   P-value                                        0.000       0.000
##   Scaling correction factor                                  2.082
##
## User Model versus Baseline Model:
##
##   Comparative Fit Index (CFI)                    0.999       0.987
##   Tucker-Lewis Index (TLI)                       0.997       0.976
##
##   Robust Comparative Fit Index (CFI)                         0.970
##   Robust Tucker-Lewis Index (TLI)                            0.944
##
## Root Mean Square Error of Approximation:
##
##   RMSEA                                          0.074       0.153
##   90 Percent confidence interval - lower         0.048       0.128
##   90 Percent confidence interval - upper         0.102       0.179
##   P-value H_0: RMSEA <= 0.050                    0.063       0.000
##   P-value H_0: RMSEA >= 0.080                    0.391       1.000
##
##   Robust RMSEA                                               0.127
##   90 Percent confidence interval - lower                     0.106
##   90 Percent confidence interval - upper                     0.149
##   P-value H_0: Robust RMSEA <= 0.050                         0.000
##   P-value H_0: Robust RMSEA >= 0.080                         1.000
##
## Standardized Root Mean Square Residual:
##
##   SRMR                                           0.028       0.028
##
## Parameter Estimates:
##
##   Standard errors                             Robust.sem
##   Information                                   Expected
##   Information saturated (h1) model          Unstructured
##
## Latent Variables:
##                        Estimate  Std.Err  z-value  P(>|z|)   Std.lv  Std.all
##   format_preference =~
##     abq1_rev              1.000                               0.494    0.494
##     abq2                  1.769    0.102   17.310    0.000    0.875    0.875
##     abq3                  1.841    0.108   17.044    0.000    0.910    0.910
##     abq4                  1.659    0.098   16.932    0.000    0.820    0.820
##     abq5                  1.665    0.097   17.130    0.000    0.823    0.823
##     abq6_rev              1.570    0.080   19.619    0.000    0.776    0.776
##
## Covariances:
##                    Estimate  Std.Err  z-value  P(>|z|)   Std.lv  Std.all
##  .abq1_rev ~~
##    .abq6_rev          0.269    0.023   11.870    0.000    0.269    0.490
##
## Intercepts:
##                    Estimate  Std.Err  z-value  P(>|z|)   Std.lv  Std.all
##    .abq1_rev          0.000                               0.000    0.000
##    .abq2              0.000                               0.000    0.000
##    .abq3              0.000                               0.000    0.000
##    .abq4              0.000                               0.000    0.000
##    .abq5              0.000                               0.000    0.000
##    .abq6_rev          0.000                               0.000    0.000
##     format_prefrnc    0.000                               0.000    0.000
##
## Thresholds:
##                    Estimate  Std.Err  z-value  P(>|z|)   Std.lv  Std.all
##     abq1_rev|t1      -0.959    0.064  -14.957    0.000   -0.959   -0.959
##     abq1_rev|t2      -0.232    0.055   -4.257    0.000   -0.232   -0.232
##     abq1_rev|t3       0.349    0.055    6.313    0.000    0.349    0.349
##     abq1_rev|t4       0.821    0.061   13.410    0.000    0.821    0.821
##     abq1_rev|t5       1.101    0.068   16.240    0.000    1.101    1.101
##     abq1_rev|t6       1.432    0.080   17.928    0.000    1.432    1.432
##     abq2|t1          -0.921    0.063  -14.552    0.000   -0.921   -0.921
##     abq2|t2          -0.317    0.055   -5.762    0.000   -0.317   -0.317
##     abq2|t3           0.187    0.054    3.444    0.001    0.187    0.187
##     abq2|t4           0.614    0.058   10.598    0.000    0.614    0.614
##     abq2|t5           1.050    0.066   15.803    0.000    1.050    1.050
##     abq2|t6           1.528    0.085   18.058    0.000    1.528    1.528
##     abq3|t1          -0.834    0.061  -13.568    0.000   -0.834   -0.834
##     abq3|t2          -0.161    0.054   -2.968    0.003   -0.161   -0.161
##     abq3|t3           0.314    0.055    5.714    0.000    0.314    0.314
##     abq3|t4           0.814    0.061   13.330    0.000    0.814    0.814
##     abq3|t5           1.335    0.076   17.625    0.000    1.335    1.335
##     abq3|t6           1.914    0.111   17.251    0.000    1.914    1.914
##     abq4|t1          -0.637    0.058  -10.932    0.000   -0.637   -0.637
##     abq4|t2          -0.014    0.054   -0.258    0.796   -0.014   -0.014
##     abq4|t3           0.553    0.057    9.676    0.000    0.553    0.553
##     abq4|t4           1.108    0.068   16.283    0.000    1.108    1.108
##     abq4|t5           1.499    0.083   18.030    0.000    1.499    1.499
##     abq4|t6           2.045    0.124   16.516    0.000    2.045    2.045
##     abq5|t1          -0.958    0.064  -14.930    0.000   -0.958   -0.958
##     abq5|t2          -0.317    0.055   -5.762    0.000   -0.317   -0.317
##     abq5|t3           0.051    0.054    0.948    0.343    0.051    0.051
##     abq5|t4           0.677    0.059   11.512    0.000    0.677    0.677
##     abq5|t5           1.050    0.066   15.803    0.000    1.050    1.050
##     abq5|t6           1.625    0.090   18.056    0.000    1.625    1.625
##     abq6_rev|t1      -1.049    0.066  -15.778    0.000   -1.049   -1.049
##     abq6_rev|t2      -0.219    0.055   -4.007    0.000   -0.219   -0.219
##     abq6_rev|t3       0.176    0.054    3.232    0.001    0.176    0.176
##     abq6_rev|t4       0.773    0.060   12.790    0.000    0.773    0.773
##     abq6_rev|t5       1.198    0.071   16.894    0.000    1.198    1.198
##     abq6_rev|t6       1.660    0.092   18.002    0.000    1.660    1.660
##
## Variances:
##                    Estimate  Std.Err  z-value  P(>|z|)   Std.lv  Std.all
##    .abq1_rev          0.756                               0.756    0.756
##    .abq2              0.235                               0.235    0.235
##    .abq3              0.172                               0.172    0.172
##    .abq4              0.327                               0.327    0.327
##    .abq5              0.322                               0.322    0.322
##    .abq6_rev          0.397                               0.397    0.397
##     format_prefrnc    0.244    0.029    8.367    0.000    1.000    1.000
##
## Scales y*:
##                    Estimate  Std.Err  z-value  P(>|z|)   Std.lv  Std.all
##     abq1_rev          1.000                               1.000    1.000
##     abq2              1.000                               1.000    1.000
##     abq3              1.000                               1.000    1.000
##     abq4              1.000                               1.000    1.000
##     abq5              1.000                               1.000    1.000
##     abq6_rev          1.000                               1.000    1.000
##
## R-Square:
##                    Estimate
##     abq1_rev          0.244
##     abq2              0.765
##     abq3              0.828
##     abq4              0.673
##     abq5              0.678
##     abq6_rev          0.603
```

```r
# Show the standardized results
format_preference_loadings <- standardizedsolution(m_format_preference)

format_preference_loadings
```

```
##                  lhs  op               rhs est.std    se       z pvalue
## 1  format_preference  =~          abq1_rev   0.494 0.030  16.735  0.000
## 2  format_preference  =~              abq2   0.875 0.011  82.496  0.000
## 3  format_preference  =~              abq3   0.910 0.009  99.937  0.000
## 4  format_preference  =~              abq4   0.820 0.015  55.732  0.000
## 5  format_preference  =~              abq5   0.823 0.015  56.283  0.000
## 6  format_preference  =~          abq6_rev   0.776 0.017  45.876  0.000
## 7           abq1_rev  ~~          abq6_rev   0.490 0.033  14.849  0.000
## 8           abq1_rev   |                t1  -0.959 0.064 -14.957  0.000
## 9           abq1_rev   |                t2  -0.232 0.055  -4.257  0.000
## 10          abq1_rev   |                t3   0.349 0.055   6.313  0.000
## 11          abq1_rev   |                t4   0.821 0.061  13.410  0.000
## 12          abq1_rev   |                t5   1.101 0.068  16.240  0.000
## 13          abq1_rev   |                t6   1.432 0.080  17.928  0.000
## 14              abq2   |                t1  -0.921 0.063 -14.552  0.000
## 15              abq2   |                t2  -0.317 0.055  -5.762  0.000
## 16              abq2   |                t3   0.187 0.054   3.444  0.001
## 17              abq2   |                t4   0.614 0.058  10.598  0.000
## 18              abq2   |                t5   1.050 0.066  15.803  0.000
## 19              abq2   |                t6   1.528 0.085  18.058  0.000
## 20              abq3   |                t1  -0.834 0.061 -13.568  0.000
## 21              abq3   |                t2  -0.161 0.054  -2.968  0.003
## 22              abq3   |                t3   0.314 0.055   5.714  0.000
## 23              abq3   |                t4   0.814 0.061  13.330  0.000
## 24              abq3   |                t5   1.335 0.076  17.625  0.000
## 25              abq3   |                t6   1.914 0.111  17.251  0.000
## 26              abq4   |                t1  -0.637 0.058 -10.932  0.000
## 27              abq4   |                t2  -0.014 0.054  -0.258  0.796
## 28              abq4   |                t3   0.553 0.057   9.676  0.000
## 29              abq4   |                t4   1.108 0.068  16.283  0.000
## 30              abq4   |                t5   1.499 0.083  18.030  0.000
## 31              abq4   |                t6   2.045 0.124  16.516  0.000
## 32              abq5   |                t1  -0.958 0.064 -14.930  0.000
## 33              abq5   |                t2  -0.317 0.055  -5.762  0.000
## 34              abq5   |                t3   0.051 0.054   0.948  0.343
## 35              abq5   |                t4   0.677 0.059  11.512  0.000
## 36              abq5   |                t5   1.050 0.066  15.803  0.000
## 37              abq5   |                t6   1.625 0.090  18.056  0.000
## 38          abq6_rev   |                t1  -1.049 0.066 -15.778  0.000
## 39          abq6_rev   |                t2  -0.219 0.055  -4.007  0.000
## 40          abq6_rev   |                t3   0.176 0.054   3.232  0.001
## 41          abq6_rev   |                t4   0.773 0.060  12.790  0.000
## 42          abq6_rev   |                t5   1.198 0.071  16.894  0.000
## 43          abq6_rev   |                t6   1.660 0.092  18.002  0.000
## 44          abq1_rev  ~~          abq1_rev   0.756 0.029  25.868  0.000
## 45              abq2  ~~              abq2   0.235 0.019  12.678  0.000
## 46              abq3  ~~              abq3   0.172 0.017  10.353  0.000
## 47              abq4  ~~              abq4   0.327 0.024  13.539  0.000
## 48              abq5  ~~              abq5   0.322 0.024  13.369  0.000
## 49          abq6_rev  ~~          abq6_rev   0.397 0.026  15.125  0.000
## 50 format_preference  ~~ format_preference   1.000 0.000      NA     NA
## 51          abq1_rev ~*~          abq1_rev   1.000 0.000      NA     NA
## 52              abq2 ~*~              abq2   1.000 0.000      NA     NA
## 53              abq3 ~*~              abq3   1.000 0.000      NA     NA
## 54              abq4 ~*~              abq4   1.000 0.000      NA     NA
## 55              abq5 ~*~              abq5   1.000 0.000      NA     NA
## 56          abq6_rev ~*~          abq6_rev   1.000 0.000      NA     NA
## 57          abq1_rev  ~1                     0.000 0.000      NA     NA
## 58              abq2  ~1                     0.000 0.000      NA     NA
## 59              abq3  ~1                     0.000 0.000      NA     NA
## 60              abq4  ~1                     0.000 0.000      NA     NA
## 61              abq5  ~1                     0.000 0.000      NA     NA
## 62          abq6_rev  ~1                     0.000 0.000      NA     NA
## 63 format_preference  ~1                     0.000 0.000      NA     NA
##    ci.lower ci.upper
## 1     0.436    0.552
## 2     0.854    0.895
## 3     0.892    0.928
## 4     0.792    0.849
## 5     0.795    0.852
## 6     0.743    0.809
## 7     0.426    0.555
## 8    -1.084   -0.833
## 9    -0.339   -0.125
## 10    0.240    0.457
## 11    0.701    0.941
## 12    0.968    1.234
## 13    1.275    1.589
## 14   -1.045   -0.797
## 15   -0.425   -0.209
## 16    0.081    0.294
## 17    0.501    0.728
## 18    0.920    1.180
## 19    1.363    1.694
## 20   -0.954   -0.713
## 21   -0.268   -0.055
## 22    0.207    0.422
## 23    0.694    0.934
## 24    1.187    1.484
## 25    1.696    2.131
## 26   -0.751   -0.523
## 27   -0.120    0.092
## 28    0.441    0.666
## 29    0.975    1.242
## 30    1.336    1.662
## 31    1.802    2.287
## 32   -1.083   -0.832
## 33   -0.425   -0.209
## 34   -0.055    0.157
## 35    0.562    0.793
## 36    0.920    1.180
## 37    1.449    1.802
## 38   -1.179   -0.919
## 39   -0.326   -0.112
## 40    0.069    0.283
## 41    0.655    0.892
## 42    1.059    1.337
## 43    1.480    1.841
## 44    0.698    0.813
## 45    0.199    0.271
## 46    0.139    0.204
## 47    0.280    0.374
## 48    0.275    0.369
## 49    0.346    0.449
## 50    1.000    1.000
## 51    1.000    1.000
## 52    1.000    1.000
## 53    1.000    1.000
## 54    1.000    1.000
## 55    1.000    1.000
## 56    1.000    1.000
## 57    0.000    0.000
## 58    0.000    0.000
## 59    0.000    0.000
## 60    0.000    0.000
## 61    0.000    0.000
## 62    0.000    0.000
## 63    0.000    0.000
```

```r
# Calculate factor scores
format_preference_score <- predict(m_format_preference)

# Add the calculated factor scores to the data file
tib <- cbind(tib, format_preference_score)
```

Trust in Health Authorities

```r
# Create the model
trust_hca <- "trust_hca =~ trust_info + trust_recommend + trust_other_rev + trust_intent"

# Conduct the CFA
m_trust_hca <- cfa(trust_hca,
                   estimator = "WLSMV",
                   missing = "pairwise",
                   ordered = TRUE,
                   data = tib)

# Show the results
summary(m_trust_hca,
        fit.measures = TRUE,
        standardized = TRUE,
        rsquare = TRUE)
```

```
## lavaan 0.6.15 ended normally after 17 iterations
##
##   Estimator                                       DWLS
##   Optimization method                           NLMINB
##   Number of model parameters                        28
##
##   Number of observations                           540
##   Number of missing patterns                         3
##
## Model Test User Model:
##                                               Standard      Scaled
##   Test Statistic                                 5.280      30.848
##   Degrees of freedom                                 2           2
##   P-value (Chi-square)                           0.071       0.000
##   Scaling correction factor                                  0.171
##   Shift parameter                                            0.019
##     simple second-order correction
##
## Model Test Baseline Model:
##
##   Test statistic                             40832.293   24336.710
##   Degrees of freedom                                 6           6
##   P-value                                        0.000       0.000
##   Scaling correction factor                                  1.678
##
## User Model versus Baseline Model:
##
##   Comparative Fit Index (CFI)                    1.000       0.999
##   Tucker-Lewis Index (TLI)                       1.000       0.996
##
##   Robust Comparative Fit Index (CFI)                         0.990
##   Robust Tucker-Lewis Index (TLI)                            0.971
##
## Root Mean Square Error of Approximation:
##
##   RMSEA                                          0.055       0.164
##   90 Percent confidence interval - lower         0.000       0.116
##   90 Percent confidence interval - upper         0.115       0.217
##   P-value H_0: RMSEA <= 0.050                    0.349       0.000
##   P-value H_0: RMSEA >= 0.080                    0.295       0.997
##
##   Robust RMSEA                                               0.153
##   90 Percent confidence interval - lower                     0.108
##   90 Percent confidence interval - upper                     0.203
##   P-value H_0: Robust RMSEA <= 0.050                         0.000
##   P-value H_0: Robust RMSEA >= 0.080                         0.995
##
## Standardized Root Mean Square Residual:
##
##   SRMR                                           0.012       0.012
##
## Parameter Estimates:
##
##   Standard errors                             Robust.sem
##   Information                                   Expected
##   Information saturated (h1) model          Unstructured
##
## Latent Variables:
##                    Estimate  Std.Err  z-value  P(>|z|)   Std.lv  Std.all
##   trust_hca =~
##     trust_info        1.000                               0.941    0.941
##     trust_recommnd    1.017    0.009  115.282    0.000    0.957    0.957
##     trust_other_rv    0.885    0.015   58.991    0.000    0.832    0.832
##     trust_intent      1.012    0.008  124.382    0.000    0.953    0.953
##
## Intercepts:
##                    Estimate  Std.Err  z-value  P(>|z|)   Std.lv  Std.all
##    .trust_info        0.000                               0.000    0.000
##    .trust_recommnd    0.000                               0.000    0.000
##    .trust_other_rv    0.000                               0.000    0.000
##    .trust_intent      0.000                               0.000    0.000
##     trust_hca         0.000                               0.000    0.000
##
## Thresholds:
##                    Estimate  Std.Err  z-value  P(>|z|)   Std.lv  Std.all
##     trust_info|t1    -0.704    0.059  -11.903    0.000   -0.704   -0.704
##     trust_info|t2    -0.467    0.056   -8.307    0.000   -0.467   -0.467
##     trust_info|t3    -0.282    0.055   -5.153    0.000   -0.282   -0.282
##     trust_info|t4    -0.107    0.054   -1.977    0.048   -0.107   -0.107
##     trust_info|t5     0.135    0.054    2.493    0.013    0.135    0.135
##     trust_info|t6     0.790    0.061   13.039    0.000    0.790    0.790
##     trst_rcmmnd|t1   -0.692    0.059  -11.739    0.000   -0.692   -0.692
##     trst_rcmmnd|t2   -0.405    0.056   -7.288    0.000   -0.405   -0.405
##     trst_rcmmnd|t3   -0.191    0.054   -3.523    0.000   -0.191   -0.191
##     trst_rcmmnd|t4    0.000    0.054    0.000    1.000    0.000    0.000
##     trst_rcmmnd|t5    0.258    0.055    4.724    0.000    0.258    0.258
##     trst_rcmmnd|t6    0.796    0.061   13.119    0.000    0.796    0.796
##     trst_thr_rv|t1   -1.026    0.066  -15.590    0.000   -1.026   -1.026
##     trst_thr_rv|t2   -0.774    0.060  -12.819    0.000   -0.774   -0.774
##     trst_thr_rv|t3   -0.532    0.057   -9.338    0.000   -0.532   -0.532
##     trst_thr_rv|t4   -0.197    0.054   -3.616    0.000   -0.197   -0.197
##     trst_thr_rv|t5    0.037    0.054    0.689    0.491    0.037    0.037
##     trst_thr_rv|t6    0.500    0.057    8.831    0.000    0.500    0.500
##     trust_intnt|t1   -0.937    0.064  -14.731    0.000   -0.937   -0.937
##     trust_intnt|t2   -0.566    0.057   -9.877    0.000   -0.566   -0.566
##     trust_intnt|t3   -0.334    0.055   -6.056    0.000   -0.334   -0.334
##     trust_intnt|t4   -0.086    0.054   -1.592    0.111   -0.086   -0.086
##     trust_intnt|t5    0.213    0.054    3.913    0.000    0.213    0.213
##     trust_intnt|t6    0.739    0.060   12.363    0.000    0.739    0.739
##
## Variances:
##                    Estimate  Std.Err  z-value  P(>|z|)   Std.lv  Std.all
##    .trust_info        0.115                               0.115    0.115
##    .trust_recommnd    0.083                               0.083    0.083
##    .trust_other_rv    0.307                               0.307    0.307
##    .trust_intent      0.093                               0.093    0.093
##     trust_hca         0.885    0.013   69.914    0.000    1.000    1.000
##
## Scales y*:
##                    Estimate  Std.Err  z-value  P(>|z|)   Std.lv  Std.all
##     trust_info        1.000                               1.000    1.000
##     trust_recommnd    1.000                               1.000    1.000
##     trust_other_rv    1.000                               1.000    1.000
##     trust_intent      1.000                               1.000    1.000
##
## R-Square:
##                    Estimate
##     trust_info        0.885
##     trust_recommnd    0.917
##     trust_other_rv    0.693
##     trust_intent      0.907
```

```r
# Show the standardized results
trust_loadings <- standardizedsolution(m_trust_hca)

trust_loadings
```

```
##                lhs  op             rhs est.std    se       z pvalue ci.lower
## 1        trust_hca  =~      trust_info   0.941 0.007 139.828  0.000    0.928
## 2        trust_hca  =~ trust_recommend   0.957 0.005 180.601  0.000    0.947
## 3        trust_hca  =~ trust_other_rev   0.832 0.015  56.972  0.000    0.804
## 4        trust_hca  =~    trust_intent   0.953 0.005 187.072  0.000    0.943
## 5       trust_info   |              t1  -0.704 0.059 -11.903  0.000   -0.820
## 6       trust_info   |              t2  -0.467 0.056  -8.307  0.000   -0.577
## 7       trust_info   |              t3  -0.282 0.055  -5.153  0.000   -0.390
## 8       trust_info   |              t4  -0.107 0.054  -1.977  0.048   -0.213
## 9       trust_info   |              t5   0.135 0.054   2.493  0.013    0.029
## 10      trust_info   |              t6   0.790 0.061  13.039  0.000    0.671
## 11 trust_recommend   |              t1  -0.692 0.059 -11.739  0.000   -0.808
## 12 trust_recommend   |              t2  -0.405 0.056  -7.288  0.000   -0.514
## 13 trust_recommend   |              t3  -0.191 0.054  -3.523  0.000   -0.298
## 14 trust_recommend   |              t4   0.000 0.054   0.000  1.000   -0.106
## 15 trust_recommend   |              t5   0.258 0.055   4.724  0.000    0.151
## 16 trust_recommend   |              t6   0.796 0.061  13.119  0.000    0.677
## 17 trust_other_rev   |              t1  -1.026 0.066 -15.590  0.000   -1.155
## 18 trust_other_rev   |              t2  -0.774 0.060 -12.819  0.000   -0.893
## 19 trust_other_rev   |              t3  -0.532 0.057  -9.338  0.000   -0.644
## 20 trust_other_rev   |              t4  -0.197 0.054  -3.616  0.000   -0.304
## 21 trust_other_rev   |              t5   0.037 0.054   0.689  0.491   -0.069
## 22 trust_other_rev   |              t6   0.500 0.057   8.831  0.000    0.389
## 23    trust_intent   |              t1  -0.937 0.064 -14.731  0.000   -1.062
## 24    trust_intent   |              t2  -0.566 0.057  -9.877  0.000   -0.678
## 25    trust_intent   |              t3  -0.334 0.055  -6.056  0.000   -0.442
## 26    trust_intent   |              t4  -0.086 0.054  -1.592  0.111   -0.192
## 27    trust_intent   |              t5   0.213 0.054   3.913  0.000    0.106
## 28    trust_intent   |              t6   0.739 0.060  12.363  0.000    0.622
## 29      trust_info  ~~      trust_info   0.115 0.013   9.060  0.000    0.090
## 30 trust_recommend  ~~ trust_recommend   0.083 0.010   8.224  0.000    0.064
## 31 trust_other_rev  ~~ trust_other_rev   0.307 0.024  12.638  0.000    0.260
## 32    trust_intent  ~~    trust_intent   0.093 0.010   9.556  0.000    0.074
## 33       trust_hca  ~~       trust_hca   1.000 0.000      NA     NA    1.000
## 34      trust_info ~*~      trust_info   1.000 0.000      NA     NA    1.000
## 35 trust_recommend ~*~ trust_recommend   1.000 0.000      NA     NA    1.000
## 36 trust_other_rev ~*~ trust_other_rev   1.000 0.000      NA     NA    1.000
## 37    trust_intent ~*~    trust_intent   1.000 0.000      NA     NA    1.000
## 38      trust_info  ~1                   0.000 0.000      NA     NA    0.000
## 39 trust_recommend  ~1                   0.000 0.000      NA     NA    0.000
## 40 trust_other_rev  ~1                   0.000 0.000      NA     NA    0.000
## 41    trust_intent  ~1                   0.000 0.000      NA     NA    0.000
## 42       trust_hca  ~1                   0.000 0.000      NA     NA    0.000
##    ci.upper
## 1     0.954
## 2     0.968
## 3     0.861
## 4     0.963
## 5    -0.588
## 6    -0.357
## 7    -0.175
## 8    -0.001
## 9     0.241
## 10    0.909
## 11   -0.577
## 12   -0.296
## 13   -0.085
## 14    0.106
## 15    0.365
## 16    0.915
## 17   -0.897
## 18   -0.656
## 19   -0.420
## 20   -0.090
## 21    0.143
## 22    0.611
## 23   -0.812
## 24   -0.454
## 25   -0.226
## 26    0.020
## 27    0.320
## 28    0.856
## 29    0.140
## 30    0.103
## 31    0.355
## 32    0.112
## 33    1.000
## 34    1.000
## 35    1.000
## 36    1.000
## 37    1.000
## 38    0.000
## 39    0.000
## 40    0.000
## 41    0.000
## 42    0.000
```

```r
# Calculate factor scores
trust_hca_score <- predict(m_trust_hca)

# Add the calculated factor scores to the data file
tib <- cbind(tib, trust_hca_score)
```

Conspiracy Mentality

```r
# Create the model
conspiracy_mentality <- "conspiracy_mentality =~ cmq1 + cmq2 + cmq3 + cmq4 + cmq5
                                                 cmq1 ~~ cmq2"

# Conduct the CFA
m_conspiracy_mentality <- cfa(conspiracy_mentality,
                              estimator = "WLSMV",
                              missing = "pairwise",
                              ordered = TRUE,
                              data = tib)

# Show the results
summary(m_conspiracy_mentality,
        fit.measures = TRUE,
        standardized = TRUE,
        rsquare = TRUE)
```

```
## lavaan 0.6.15 ended normally after 21 iterations
##
##   Estimator                                       DWLS
##   Optimization method                           NLMINB
##   Number of model parameters                        55
##
##   Number of observations                           540
##   Number of missing patterns                         3
##
## Model Test User Model:
##                                               Standard      Scaled
##   Test Statistic                                18.392      52.350
##   Degrees of freedom                                 4           4
##   P-value (Chi-square)                           0.001       0.000
##   Scaling correction factor                                  0.354
##   Shift parameter                                            0.447
##     simple second-order correction
##
## Model Test Baseline Model:
##
##   Test statistic                             12167.069    7142.631
##   Degrees of freedom                                10          10
##   P-value                                        0.000       0.000
##   Scaling correction factor                                  1.704
##
## User Model versus Baseline Model:
##
##   Comparative Fit Index (CFI)                    0.999       0.993
##   Tucker-Lewis Index (TLI)                       0.997       0.983
##
##   Robust Comparative Fit Index (CFI)                         0.972
##   Robust Tucker-Lewis Index (TLI)                            0.929
##
## Root Mean Square Error of Approximation:
##
##   RMSEA                                          0.082       0.150
##   90 Percent confidence interval - lower         0.046       0.115
##   90 Percent confidence interval - upper         0.121       0.187
##   P-value H_0: RMSEA <= 0.050                    0.068       0.000
##   P-value H_0: RMSEA >= 0.080                    0.577       0.999
##
##   Robust RMSEA                                               0.158
##   90 Percent confidence interval - lower                     0.124
##   90 Percent confidence interval - upper                     0.196
##   P-value H_0: Robust RMSEA <= 0.050                         0.000
##   P-value H_0: Robust RMSEA >= 0.080                         1.000
##
## Standardized Root Mean Square Residual:
##
##   SRMR                                           0.025       0.025
##
## Parameter Estimates:
##
##   Standard errors                             Robust.sem
##   Information                                   Expected
##   Information saturated (h1) model          Unstructured
##
## Latent Variables:
##                           Estimate  Std.Err  z-value  P(>|z|)   Std.lv  Std.all
##   conspiracy_mentality =~
##     cmq1                     1.000                               0.725    0.725
##     cmq2                     1.037    0.025   42.266    0.000    0.752    0.752
##     cmq3                     0.949    0.033   28.408    0.000    0.688    0.688
##     cmq4                     1.255    0.036   34.431    0.000    0.909    0.909
##     cmq5                     1.269    0.036   34.877    0.000    0.919    0.919
##
## Covariances:
##                    Estimate  Std.Err  z-value  P(>|z|)   Std.lv  Std.all
##  .cmq1 ~~
##    .cmq2              0.218    0.023    9.470    0.000    0.218    0.480
##
## Intercepts:
##                    Estimate  Std.Err  z-value  P(>|z|)   Std.lv  Std.all
##    .cmq1              0.000                               0.000    0.000
##    .cmq2              0.000                               0.000    0.000
##    .cmq3              0.000                               0.000    0.000
##    .cmq4              0.000                               0.000    0.000
##    .cmq5              0.000                               0.000    0.000
##     cnsprcy_mntlty    0.000                               0.000    0.000
##
## Thresholds:
##                    Estimate  Std.Err  z-value  P(>|z|)   Std.lv  Std.all
##     cmq1|t1          -2.902    0.313   -9.266    0.000   -2.902   -2.902
##     cmq1|t2          -1.944    0.114  -17.115    0.000   -1.944   -1.944
##     cmq1|t3          -1.645    0.091  -18.071    0.000   -1.645   -1.645
##     cmq1|t4          -1.395    0.078  -17.848    0.000   -1.395   -1.395
##     cmq1|t5          -1.164    0.070  -16.718    0.000   -1.164   -1.164
##     cmq1|t6          -1.052    0.066  -15.853    0.000   -1.052   -1.052
##     cmq1|t7          -0.493    0.056   -8.731    0.000   -0.493   -0.493
##     cmq1|t8          -0.163    0.054   -3.008    0.003   -0.163   -0.163
##     cmq1|t9           0.140    0.054    2.579    0.010    0.140    0.140
##     cmq1|t10          0.498    0.056    8.815    0.000    0.498    0.498
##     cmq2|t1          -2.085    0.128  -16.276    0.000   -2.085   -2.085
##     cmq2|t2          -1.720    0.096  -17.930    0.000   -1.720   -1.720
##     cmq2|t3          -1.313    0.075  -17.533    0.000   -1.313   -1.313
##     cmq2|t4          -0.959    0.064  -14.957    0.000   -0.959   -0.959
##     cmq2|t5          -0.739    0.060  -12.363    0.000   -0.739   -0.739
##     cmq2|t6          -0.161    0.054   -2.968    0.003   -0.161   -0.161
##     cmq2|t7           0.110    0.054    2.022    0.043    0.110    0.110
##     cmq2|t8           0.502    0.057    8.865    0.000    0.502    0.502
##     cmq2|t9           0.916    0.063   14.503    0.000    0.916    0.916
##     cmq3|t1          -1.560    0.086  -18.090    0.000   -1.560   -1.560
##     cmq3|t2          -0.814    0.061  -13.330    0.000   -0.814   -0.814
```

```
##     cmq3|t3          -0.450    0.056   -8.018    0.000   -0.450   -0.450
##     cmq3|t4          -0.058    0.054   -1.076    0.282   -0.058   -0.058
##     cmq3|t5           0.237    0.055    4.342    0.000    0.237    0.237
##     cmq3|t6           0.460    0.056    8.187    0.000    0.460    0.460
##     cmq3|t7           0.763    0.060   12.688    0.000    0.763    0.763
##     cmq3|t8           0.989    0.065   15.253    0.000    0.989    0.989
##     cmq3|t9           1.370    0.077   17.752    0.000    1.370    1.370
##     cmq3|t10          1.681    0.093   18.005    0.000    1.681    1.681
##     cmq4|t1          -1.110    0.068  -16.307    0.000   -1.110   -1.110
##     cmq4|t2          -0.444    0.056   -7.933    0.000   -0.444   -0.444
##     cmq4|t3          -0.171    0.054   -3.140    0.002   -0.171   -0.171
##     cmq4|t4           0.081    0.054    1.506    0.132    0.081    0.081
##     cmq4|t5           0.266    0.055    4.857    0.000    0.266    0.266
##     cmq4|t6           0.702    0.059   11.872    0.000    0.702    0.702
##     cmq4|t7           0.959    0.064   14.957    0.000    0.959    0.959
##     cmq4|t8           1.181    0.070   16.819    0.000    1.181    1.181
##     cmq4|t9           1.472    0.082   18.007    0.000    1.472    1.472
##     cmq4|t10          1.741    0.097   17.881    0.000    1.741    1.741
##     cmq5|t1          -1.347    0.076  -17.669    0.000   -1.347   -1.347
##     cmq5|t2          -0.633    0.058  -10.880    0.000   -0.633   -0.633
##     cmq5|t3          -0.359    0.055   -6.484    0.000   -0.359   -0.359
##     cmq5|t4          -0.105    0.054   -1.936    0.053   -0.105   -0.105
##     cmq5|t5           0.035    0.054    0.645    0.519    0.035    0.035
##     cmq5|t6           0.339    0.055    6.142    0.000    0.339    0.339
##     cmq5|t7           0.633    0.058   10.880    0.000    0.633    0.633
##     cmq5|t8           0.827    0.061   13.489    0.000    0.827    0.827
##     cmq5|t9           1.084    0.067   16.105    0.000    1.084    1.084
##     cmq5|t10          1.324    0.075   17.579    0.000    1.324    1.324
##
## Variances:
##                    Estimate  Std.Err  z-value  P(>|z|)   Std.lv  Std.all
##    .cmq1              0.475                               0.475    0.475
##    .cmq2              0.435                               0.435    0.435
##    .cmq3              0.527                               0.527    0.527
##    .cmq4              0.173                               0.173    0.173
##    .cmq5              0.155                               0.155    0.155
##     cnsprcy_mntlty    0.525    0.030   17.664    0.000    1.000    1.000
##
## Scales y*:
##                    Estimate  Std.Err  z-value  P(>|z|)   Std.lv  Std.all
##     cmq1              1.000                               1.000    1.000
##     cmq2              1.000                               1.000    1.000
##     cmq3              1.000                               1.000    1.000
##     cmq4              1.000                               1.000    1.000
##     cmq5              1.000                               1.000    1.000
##
## R-Square:
##                    Estimate
##     cmq1              0.525
##     cmq2              0.565
##     cmq3              0.473
##     cmq4              0.827
##     cmq5              0.845
```

```r
# Show the standardized results
cm_loadings <- standardizedsolution(m_conspiracy_mentality)

cm_loadings
```

```
##                   lhs  op                 rhs est.std    se       z pvalue
## 1  conspiracy_mentality  =~                cmq1   0.725 0.021  35.329  0.000
## 2  conspiracy_mentality  =~                cmq2   0.752 0.018  41.591  0.000
## 3  conspiracy_mentality  =~                cmq3   0.688 0.021  32.697  0.000
## 4  conspiracy_mentality  =~                cmq4   0.909 0.011  79.200  0.000
## 5  conspiracy_mentality  =~                cmq5   0.919 0.011  86.208  0.000
## 6                  cmq1  ~~                cmq2   0.480 0.036  13.302  0.000
## 7                  cmq1   |                  t1  -2.902 0.313  -9.266  0.000
## 8                  cmq1   |                  t2  -1.944 0.114 -17.115  0.000
## 9                  cmq1   |                  t3  -1.645 0.091 -18.071  0.000
## 10                 cmq1   |                  t4  -1.395 0.078 -17.848  0.000
## 11                 cmq1   |                  t5  -1.164 0.070 -16.718  0.000
## 12                 cmq1   |                  t6  -1.052 0.066 -15.853  0.000
## 13                 cmq1   |                  t7  -0.493 0.056  -8.731  0.000
## 14                 cmq1   |                  t8  -0.163 0.054  -3.008  0.003
## 15                 cmq1   |                  t9   0.140 0.054   2.579  0.010
## 16                 cmq1   |                 t10   0.498 0.056   8.815  0.000
## 17                 cmq2   |                  t1  -2.085 0.128 -16.276  0.000
## 18                 cmq2   |                  t2  -1.720 0.096 -17.930  0.000
## 19                 cmq2   |                  t3  -1.313 0.075 -17.533  0.000
## 20                 cmq2   |                  t4  -0.959 0.064 -14.957  0.000
## 21                 cmq2   |                  t5  -0.739 0.060 -12.363  0.000
## 22                 cmq2   |                  t6  -0.161 0.054  -2.968  0.003
## 23                 cmq2   |                  t7   0.110 0.054   2.022  0.043
## 24                 cmq2   |                  t8   0.502 0.057   8.865  0.000
## 25                 cmq2   |                  t9   0.916 0.063  14.503  0.000
## 26                 cmq3   |                  t1  -1.560 0.086 -18.090  0.000
## 27                 cmq3   |                  t2  -0.814 0.061 -13.330  0.000
## 28                 cmq3   |                  t3  -0.450 0.056  -8.018  0.000
## 29                 cmq3   |                  t4  -0.058 0.054  -1.076  0.282
## 30                 cmq3   |                  t5   0.237 0.055   4.342  0.000
## 31                 cmq3   |                  t6   0.460 0.056   8.187  0.000
## 32                 cmq3   |                  t7   0.763 0.060  12.688  0.000
## 33                 cmq3   |                  t8   0.989 0.065  15.253  0.000
## 34                 cmq3   |                  t9   1.370 0.077  17.752  0.000
## 35                 cmq3   |                 t10   1.681 0.093  18.005  0.000
## 36                 cmq4   |                  t1  -1.110 0.068 -16.307  0.000
## 37                 cmq4   |                  t2  -0.444 0.056  -7.933  0.000
## 38                 cmq4   |                  t3  -0.171 0.054  -3.140  0.002
## 39                 cmq4   |                  t4   0.081 0.054   1.506  0.132
## 40                 cmq4   |                  t5   0.266 0.055   4.857  0.000
## 41                 cmq4   |                  t6   0.702 0.059  11.872  0.000
## 42                 cmq4   |                  t7   0.959 0.064  14.957  0.000
## 43                 cmq4   |                  t8   1.181 0.070  16.819  0.000
## 44                 cmq4   |                  t9   1.472 0.082  18.007  0.000
## 45                 cmq4   |                 t10   1.741 0.097  17.881  0.000
## 46                 cmq5   |                  t1  -1.347 0.076 -17.669  0.000
## 47                 cmq5   |                  t2  -0.633 0.058 -10.880  0.000
## 48                 cmq5   |                  t3  -0.359 0.055  -6.484  0.000
## 49                 cmq5   |                  t4  -0.105 0.054  -1.936  0.053
## 50                 cmq5   |                  t5   0.035 0.054   0.645  0.519
## 51                 cmq5   |                  t6   0.339 0.055   6.142  0.000
## 52                 cmq5   |                  t7   0.633 0.058  10.880  0.000
## 53                 cmq5   |                  t8   0.827 0.061  13.489  0.000
## 54                 cmq5   |                  t9   1.084 0.067  16.105  0.000
## 55                 cmq5   |                 t10   1.324 0.075  17.579  0.000
## 56                 cmq1  ~~                cmq1   0.475 0.030  15.971  0.000
## 57                 cmq2  ~~                cmq2   0.435 0.027  16.009  0.000
## 58                 cmq3  ~~                cmq3   0.527 0.029  18.222  0.000
## 59                 cmq4  ~~                cmq4   0.173 0.021   8.287  0.000
## 60                 cmq5  ~~                cmq5   0.155 0.020   7.894  0.000
## 61 conspiracy_mentality  ~~ conspiracy_mentality   1.000 0.000      NA     NA
## 62                 cmq1 ~*~                cmq1   1.000 0.000      NA     NA
## 63                 cmq2 ~*~                cmq2   1.000 0.000      NA     NA
## 64                 cmq3 ~*~                cmq3   1.000 0.000      NA     NA
## 65                 cmq4 ~*~                cmq4   1.000 0.000      NA     NA
## 66                 cmq5 ~*~                cmq5   1.000 0.000      NA     NA
## 67                 cmq1  ~1                       0.000 0.000      NA     NA
## 68                 cmq2  ~1                       0.000 0.000      NA     NA
## 69                 cmq3  ~1                       0.000 0.000      NA     NA
## 70                 cmq4  ~1                       0.000 0.000      NA     NA
## 71                 cmq5  ~1                       0.000 0.000      NA     NA
## 72 conspiracy_mentality  ~1                       0.000 0.000      NA     NA
##    ci.lower ci.upper
## 1     0.684    0.765
## 2     0.716    0.787
## 3     0.646    0.729
## 4     0.887    0.932
## 5     0.898    0.940
## 6     0.409    0.551
## 7    -3.516   -2.288
## 8    -2.167   -1.722
## 9    -1.823   -1.466
## 10   -1.548   -1.242
## 11   -1.300   -1.027
## 12   -1.183   -0.922
## 13   -0.603   -0.382
## 14   -0.270   -0.057
## 15    0.034    0.246
## 16    0.387    0.609
## 17   -2.336   -1.834
## 18   -1.909   -1.532
## 19   -1.460   -1.166
## 20   -1.084   -0.833
## 21   -0.856   -0.622
## 22   -0.268   -0.055
## 23    0.003    0.216
## 24    0.391    0.612
## 25    0.792    1.039
## 26   -1.729   -1.391
## 27   -0.934   -0.694
## 28   -0.559   -0.340
## 29   -0.164    0.048
## 30    0.130    0.344
## 31    0.350    0.570
## 32    0.645    0.881
## 33    0.862    1.116
## 34    1.219    1.521
## 35    1.498    1.864
## 36   -1.243   -0.976
## 37   -0.554   -0.335
## 38   -0.277   -0.064
## 39   -0.025    0.188
## 40    0.159    0.373
## 41    0.587    0.818
## 42    0.833    1.084
## 43    1.044    1.319
## 44    1.312    1.632
## 45    1.550    1.932
## 46   -1.496   -1.197
## 47   -0.747   -0.519
## 48   -0.467   -0.250
## 49   -0.211    0.001
## 50   -0.071    0.141
## 51    0.231    0.447
## 52    0.519    0.747
## 53    0.707    0.947
## 54    0.952    1.216
## 55    1.176    1.472
## 56    0.417    0.533
## 57    0.382    0.488
## 58    0.470    0.584
## 59    0.132    0.214
## 60    0.116    0.193
## 61    1.000    1.000
## 62    1.000    1.000
## 63    1.000    1.000
## 64    1.000    1.000
## 65    1.000    1.000
## 66    1.000    1.000
## 67    0.000    0.000
## 68    0.000    0.000
## 69    0.000    0.000
## 70    0.000    0.000
## 71    0.000    0.000
## 72    0.000    0.000
```

```r
# Calculate factor scores
conspiracy_mentality_score <- predict(m_conspiracy_mentality)

# Add the calculated factor scores to the data file
tib <- cbind(tib, conspiracy_mentality_score)
```

Hierarchical Cluster Analysis

Data preparations

```r
# Drop observations with na in the variables used for clustering
tib_hca <- tib %>%
  drop_na(trust_vaccines,
          trust_hca,
          format_preference,
          conspiracy_mentality,
          i_vaccine_easy_pre,
          i_vaccine_safe_pre,
          i_vaccine_efficacy_pre,
          i_threat_pre,
          i_altruism_pre,
          i_vaccine_probability_pre)

# Create z-transformed scores for the variables used for clustering
tib_hca <- tib_hca %>%
  mutate(trust_vaccines_z = as.numeric(scale(trust_vaccines)),
         trust_hca_z = as.numeric(scale(trust_hca)),
         format_preference_z = as.numeric(scale(format_preference)),
         conspiracy_mentality_z = as.numeric(scale(conspiracy_mentality)),
         i_vaccine_easy_pre_z = as.numeric(scale(i_vaccine_easy_pre)),
         i_vaccine_safe_pre_z = as.numeric(scale(i_vaccine_safe_pre)),
         i_vaccine_efficacy_pre_z = as.numeric(scale(i_vaccine_efficacy_pre)),
         i_threat_pre_z = as.numeric(scale(i_threat_pre)),
         i_altruism_pre_z = as.numeric(scale(i_altruism_pre)),
         i_vaccine_probability_pre_z = as.numeric(scale(i_vaccine_probability_pre)))

# Create a new data frame with only the relevant variables for clustering
tib_hca_variables <- tib_hca %>%
  select(trust_vaccines_z,
         trust_hca_z,
         format_preference_z,
         conspiracy_mentality_z,
         i_vaccine_easy_pre_z,
         i_vaccine_safe_pre_z,
         i_vaccine_efficacy_pre_z,
         i_threat_pre_z,
         i_altruism_pre_z,
         i_vaccine_probability_pre_z)
```

Create a distance matrix and conduct the hierarchical cluster analysis

```r
# Create a distance matrix with euclidean distances
d <- dist(tib_hca_variables, method = "euclidean")

# Conduct hierarchical clustering using Ward's method
hc_ward <- hclust(d, method = "ward.D2")
```

Plot the dendrogram

```r
# Ward dendrogram
plot(hc_ward,
     cex = 0.5,
     hang = -1,
     labels = FALSE,
     xlab = "",
     ylab = "",
     main = "",
     sub = "")
```

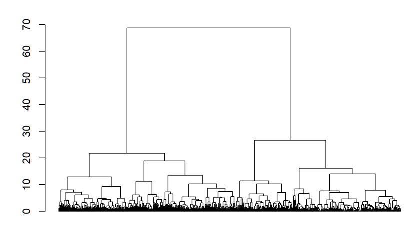

Determine the optimal number of clusters

```r
# Calculate the Duda-Hart Je(2)/Je(1) value (bigger = better)
duda <- NbClust(tib_hca_variables,
                distance = "euclidean",
                method = "ward.D2",
                max.nc = 10,
                index = "duda")

# Calculate the associated pseudo-T2 value (smaller = better)
pseudot2 <- NbClust(tib_hca_variables,
                    distance = "euclidean",
                    method = "ward.D2",
                    max.nc = 10,
                    index = "pseudot2")

# Show Duda-values
duda$All.index
```

```
##      2      3      4      5      6      7      8      9     10
## 0.7433 0.8563 0.8172 0.7987 0.7398 0.8307 0.8076 0.8321 0.7531
```

```r
# Show pseudo-T2 values
pseudot2$All.index
```

```
##       2       3       4       5       6       7       8       9      10
## 89.1021 46.4789 37.8154 42.3293 45.7219 22.8244 25.2497 17.7525 18.0340
```

```r
# Show best cluster solution
duda$Best.nc
```

```
## Number_clusters     Value_Index
##          3.0000          0.8563
```

Visualize the groups

```r
# Ward dendrogram
plot(hc_ward,
     cex = 0.6,
     hang = -1,
     labels = FALSE)

# Show the Ward groups on the dendrogram
rect.hclust(hc_ward,
            k = 3,
            border = 8:14)
```

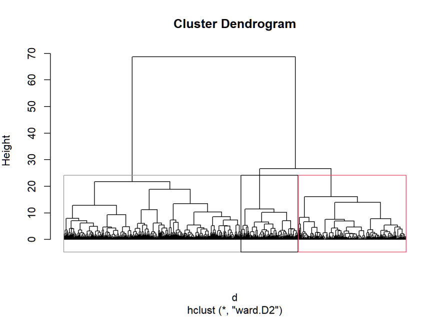

Insert cluster membership into the original data file

```r
# Create 6 groups based on the Ward clustering results
groups_ward <- cutree(hc_ward, k = 3)

# Check the group sizes
table(groups_ward)
```

```
## groups_ward
##   1   2   3
## 279 170  90
```

```r
# Insert cluster membership
tib_hca$Cluster <- assignCluster(tib_hca_variables, tib_hca, groups_ward)

tib_hca <- tib_hca %>%
  mutate(Cluster = as.factor(Cluster))
```

Visualizations

Data wrangling for visualizations

```r
# Create a new data frame with cluster means
cluster_means <- tib_hca %>%
  select(Cluster,
         trust_hca_z,
         trust_vaccines_z,
         i_vaccine_easy_pre_z,
         i_vaccine_safe_pre_z,
         i_vaccine_efficacy_pre_z,
         i_threat_pre_z,
         i_altruism_pre_z,
         format_preference_z,
         conspiracy_mentality_z,
         i_vaccine_probability_pre_z) %>%
  group_by(Cluster) %>%
  summarise(trust_hca_z_mean = mean(trust_hca_z),
            trust_vaccines_z_mean = mean(trust_vaccines_z),
            i_vaccine_easy_pre_z_mean = mean(i_vaccine_easy_pre_z),
            i_vaccine_safe_pre_z_mean = mean(i_vaccine_safe_pre_z),
            i_vaccine_efficacy_pre_z_mean = mean(i_vaccine_efficacy_pre_z),
            i_threat_pre_z_mean = mean(i_threat_pre_z),
            i_altruism_pre_z_mean = mean(i_altruism_pre_z),
            format_preference_z_mean = mean(format_preference_z),
            conspiracy_mentality_z_mean = mean(conspiracy_mentality_z),
            i_vaccine_probability_pre_z_mean = mean(i_vaccine_probability_pre_z))

# Rename the clusters
cluster_means <- cluster_means %>%
  mutate(Cluster = dplyr::recode(Cluster,
                                 "1" = "The Vaccination Inclined",
                                 "2" = "The Vaccination Opponent",
                                 "3" = "The Complacent"),
         Cluster = factor(Cluster, c("The Vaccination Inclined",
                                     "The Complacent",
                                     "The Vaccination Opponent")))
```

Visualize the results with parallel coordinate plots

```r
# Import Times New Roman
windowsFonts(Times = windowsFont("Times New Roman"))

# Create a custom color palette (colorblind friendly)
cbbPalette <- c("#0072B2",
                "#009E73",
                "#C81E17")

# Plot with cluster means
cluster_means_pcp <- cluster_means %>%
  select(Cluster,
         format_preference_z_mean,
         conspiracy_mentality_z_mean,
         trust_hca_z_mean,
         i_vaccine_easy_pre_z_mean,
         i_altruism_pre_z_mean,
         i_threat_pre_z_mean,
         i_vaccine_efficacy_pre_z_mean,
         i_vaccine_safe_pre_z_mean,
         trust_vaccines_z_mean,
         i_vaccine_probability_pre_z_mean)

# Reverse code format preference and conspiracy mentality
cluster_means_pcp <- cluster_means_pcp %>%
 mutate(format_preference_z_mean = format_preference_z_mean * (-1),
        conspiracy_mentality_z_mean = conspiracy_mentality_z_mean * (-1))

# Plot with cluster means
ggparcoord(cluster_means_pcp,
           columns = 2:11,
           groupColumn = 1,
           showPoints = FALSE,
           scale = "globalminmax") +
        theme_minimal() + # a nice clean theme
        theme(text = element_text(size = 12, family = "Times"),
              panel.grid.major.y = element_blank(),
              legend.title = element_blank()) +
        coord_flip() +
        geom_hline(yintercept = 0,
                   linewidth = 1,
                   color = "darkgrey",
                   linetype = "dashed") +
        geom_line(size = 1.3) +
        scale_colour_manual(values = cbbPalette) +
        ylab("Standardized Score") +
        scale_x_discrete("", labels = c("format_preference_z_mean" = "Format Preference*",
                                        "conspiracy_mentality_z_mean" = "Conspiracy Mentality*",
                                        "i_altruism_pre_z_mean" = "Vaccination Altruism",
                                        "i_threat_pre_z_mean" = "Disease Threat",
                                        "i_vaccine_efficacy_pre_z_mean" = "Vaccine Efficacy",
                                        "i_vaccine_safe_pre_z_mean" = "Specific Vaccine Safety",
                                        "i_vaccine_easy_pre_z_mean" = "Vaccination Convenience",
                                        "trust_vaccines_z_mean" = "General Vaccine Safety",
                                        "trust_hca_z_mean" = "Trust in Health Authorities",
                                        "i_vaccine_probability_pre_z_mean" = "Seasonal Vaccine"))
```

```
## Warning: Using `size` aesthetic for lines was deprecated in ggplot2 3.4.0.
## ℹ Please use `linewidth` instead.
## This warning is displayed once every 8 hours.
## Call `lifecycle::last_lifecycle_warnings()` to see where this warning was
## generated.
```

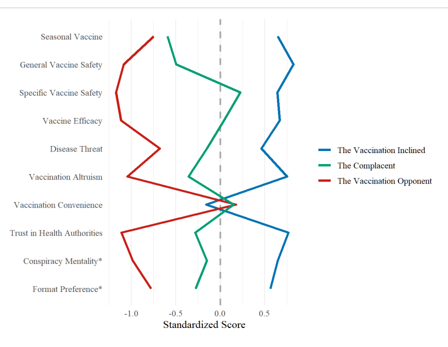

Analyzing Group Differences in Background Variables

Rename clusters

```r
# Rename the clusters and order clusters
tib_hca <- tib_hca %>%
  mutate(Cluster = dplyr::recode(Cluster,
                                 "1" = "The Vaccination Inclined",
                                 "2" = "The Vaccination Opponent",
                                 "3" = "The Complacent"),
         Cluster = factor(Cluster, c("The Vaccination Inclined",
                                     "The Complacent",
                                     "The Vaccination Opponent")))
```

Age

```r
m1_age <- aov(age ~ Cluster, data = tib_hca)

m1_age_results <- m1_age %>% tidy()

m1_age_results
```

```
## # A tibble: 2 × 6
##   term         df  sumsq meansq statistic  p.value
##   <chr>     <dbl>  <dbl>  <dbl>     <dbl>    <dbl>
## 1 Cluster       2  2058.  1029.      5.47  0.00450
## 2 Residuals   470 88475.   188.     NA    NA
```

```r
t_age <- pairwise.t.test(tib_hca$age, tib_hca$Cluster) %>% tidy()

t_age
```

```
## # A tibble: 3 × 3
##   group1                   group2                   p.value
##   <chr>                    <chr>                      <dbl>
## 1 The Complacent           The Vaccination Inclined 0.00630
## 2 The Vaccination Opponent The Vaccination Inclined 0.0878
## 3 The Vaccination Opponent The Complacent           0.186
```

```r
ggplot(m1_age, aes(x = Cluster,
                   y = age,
                   color = Cluster)) +
  geom_boxplot() +
  theme_bw() +
  stat_summary(geom = "crossbar",
               fun = "mean",
               color = "red")
```

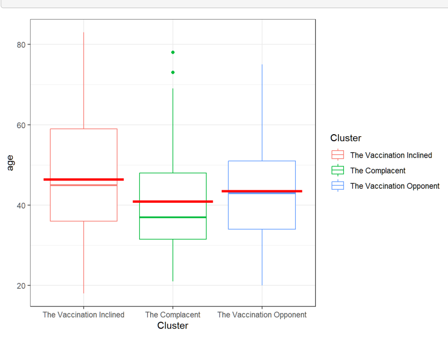

Gender

```r
tib_gender <- tib_hca %>%
  dplyr::filter(sex == "1" | sex == "2") %>%
  droplevels() %>%
  drop_na(Cluster) %>%
  mutate(gender = dplyr::recode(sex,
                                "1" = "Man",
                                "2" = "Woman"))


tib_gender_table <- table(tib_gender$Cluster, tib_gender$gender)

chisq_gender <- chisq.test(tib_gender_table)

chisq_gender %>% tidy()
```

```
## # A tibble: 1 × 4
##   statistic p.value parameter method
##       <dbl>   <dbl>     <int> <chr>
## 1      12.8 0.00167         2 Pearson's Chi-squared test
```

```r
chisq.residuals(tib_gender_table, std = FALSE)
```

```
##
##                              Man Woman
##   The Vaccination Inclined -0.93  0.51
##   The Complacent           -1.69  0.93
##   The Vaccination Opponent  2.47 -1.36
```

```r
chisq.residuals(tib_gender_table, std = TRUE)
```

```
##
##                              Man Woman
##   The Vaccination Inclined -1.55  1.55
##   The Complacent           -2.11  2.11
##   The Vaccination Opponent  3.38 -3.38
```

```r
gender_contrib <- round(100*chisq_gender$residuals^2/chisq_gender$statistic, 3)

gender_contrib
```

```
##
##                               Man  Woman
##   The Vaccination Inclined  6.796  2.051
##   The Complacent           22.431  6.769
##   The Vaccination Opponent 47.591 14.362
```

```r
ggplot(tib_gender, aes(x = Cluster, fill = gender)) +
  geom_bar(position = "fill") +
  theme_bw() +
  labs(y = "Fraction", fill = "Gender") +
  coord_flip()
```

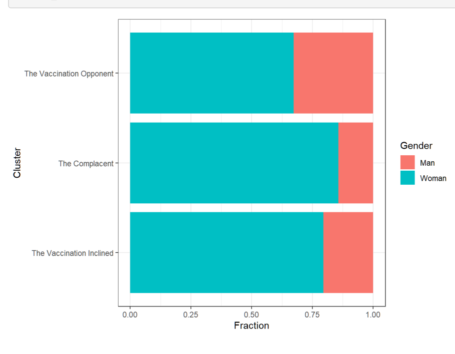

Education

```r
tib_education <- tib_hca %>%
  select(Cluster, education) %>%
  drop_na(Cluster) %>%
  filter(education != "10") %>%
  droplevels() %>%
  mutate(education = dplyr::recode(education,
                                   "1" = "Lower",
                                   "2" = "Lower",
                                   "3" = "Lower",
                                   "4" = "Lower",
                                   "5" = "Lower",
                                   "6" = "Lower",
                                   "7" = "Higher",
                                   "8" = "Higher",
                                   "9" = "Higher"),
         Cluster = factor(Cluster))

table(tib_education)
```

```
##                              education
## Cluster                      Lower Higher
##   The Vaccination Inclined     92    183
##   The Complacent               36     51
##   The Vaccination Opponent     76     86
```

```r
tib_education_table <- table(tib_education$Cluster, tib_education$education)

tib_education_table
```

```
##
##                              Lower Higher
##   The Vaccination Inclined     92    183
##   The Complacent               36     51
##   The Vaccination Opponent     76     86
```

```r
chisq.test(tib_education_table)
```

```
##
##  Pearson's Chi-squared test
##
## data:  tib_education_table
## X-squared = 8.0304, df = 2, p-value = 0.01804
```

```r
chisq_education <- chisq.test(tib_education_table)
```

```
chisq_education %>% tidy()
```

```
## # A tibble: 1 × 4
##   statistic p.value parameter method
##       <dbl>   <dbl>     <int> <chr>
## 1      8.03  0.0180         2 Pearson's Chi-squared test
```

```
chisq.residuals(tib_education_table, std = FALSE)
```

```
##
##                            Lower Higher
##   The Vaccination Inclined -1.46   1.16
##   The Complacent            0.37  -0.29
##   The Vaccination Opponent  1.63  -1.30
```

```
chisq.residuals(tib_education_table, std = TRUE)
```

```
##
##                            Lower Higher
##   The Vaccination Inclined -2.70   2.70
##   The Complacent            0.51  -0.51
##   The Vaccination Opponent  2.51  -2.51
```

```
education_contrib <- round(100*chisq_education$residuals^2/chisq_education$statistic, 3)

education_contrib
```

```
##
##                             Lower Higher
##   The Vaccination Inclined 26.384 16.820
##   The Complacent            1.668  1.063
##   The Vaccination Opponent 33.017 21.048
```

```
ggplot(tib_education, aes(x = Cluster, fill = education)) +
  geom_bar(position = "fill") +
  theme_bw() +
  labs(y = "Fraction", fill = "Level of Education") +
  coord_flip()
```

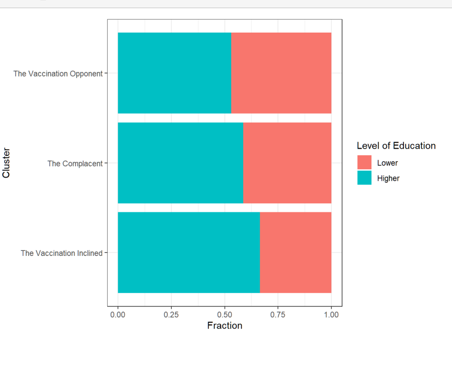

Home Region

```
tib_live <- tib_hca %>%
  select(Cluster, live) %>%
  drop_na(Cluster) %>%
  drop_na(live) %>%
  mutate(live = dplyr::recode(live,
                              "1" = "Uusimaa",
                              "2" = "Southwest Finland",
                              "3" = "Other",
                              "4" = "Other",
                              "5" = "Other",
                              "6" = "Other",
                              "7" = "Other",
                              "8" = "Other",
                              "9" = "Other",
                              "10" = "Other",
                              "11" = "Other",
                              "12" = "Other",
                              "13" = "Other",
                              "14" = "Other",
                              "15" = "Other",
                              "16" = "Other",
                              "17" = "Other",
                              "18" = "Other",
                              "19" = "Other",
                              "20" = "Other",))

tib_live_table <- table(tib_live$Cluster, tib_live$live)

chisq_live <- chisq.test(tib_live_table)

chisq_live
```

```
##
##  Pearson's Chi-squared test
##
## data:  tib_live_table
## X-squared = 14.196, df = 4, p-value = 0.006697
```

```
chisq.residuals(tib_live_table, std = FALSE)
```

```
##
##                            Uusimaa Southwest Finland Other
##   The Vaccination Inclined    0.82              0.52 -0.95
##   The Complacent             -0.35              1.90 -0.73
##   The Vaccination Opponent   -0.81             -2.07  1.77
```

```
chisq.residuals(tib_live_table, std = TRUE)
```

```
##
##                            Uusimaa Southwest Finland Other
##   The Vaccination Inclined    1.47              0.82 -1.97
##   The Complacent             -0.48              2.25 -1.14
##   The Vaccination Opponent   -1.20             -2.70  3.05
```

```
live_contrib <- round(100*chisq_live$residuals^2/chisq_live$statistic, 3)

live_contrib
```

```
##
##                            Uusimaa Southwest Finland  Other
##   The Vaccination Inclined   4.751             1.913  6.395
##   The Complacent             0.868            25.401  3.721
##   The Vaccination Opponent   4.579            30.294 22.078
```

```
ggplot(tib_live, aes(x = Cluster, fill = live)) +
  geom_bar(position = "fill") +
  theme_bw() +
  labs(y = "Fraction", fill = "Live") +
  coord_flip()
```

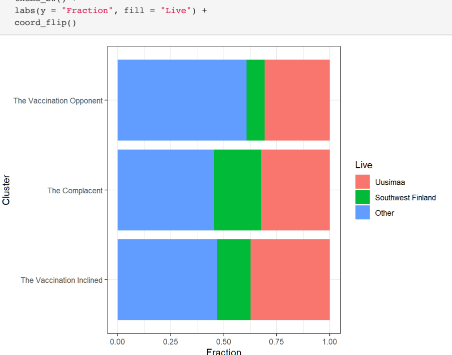

Healthcare Experience

```
tib_hcw <- tib_hca %>%
  select(Cluster, hcw) %>%
  drop_na(Cluster) %>%
  drop_na(hcw) %>%
  mutate(hcw = dplyr::recode(hcw,
                             "1" = "HC Experience",
                             "2" = "No HC Experience"))

tib_hcw_table <- table(tib_hcw$Cluster, tib_hcw$hcw)

chisq_hcw <- chisq.test(tib_hcw_table)

chisq_hcw
```

```
##
##  Pearson's Chi-squared test
##
## data:  tib_hcw_table
## X-squared = 5.1259, df = 2, p-value = 0.07708
```

```
chisq.residuals(tib_hcw_table, std = FALSE)
```

```
##
##                            HC Experience No HC Experience
##   The Vaccination Inclined         -1.24             0.64
##   The Complacent                    0.01            -0.01
##   The Vaccination Opponent          1.58            -0.82
```

```
chisq.residuals(tib_hcw_table, std = TRUE)
```

```
##
##                            HC Experience No HC Experience
##   The Vaccination Inclined         -2.01             2.01
##   The Complacent                    0.02            -0.02
##   The Vaccination Opponent          2.15            -2.15
```

```
ggplot(tib_hcw, aes(x = Cluster, fill = hcw)) +
  geom_bar(position = "fill") +
  theme_bw() +
  labs(y = "Fraction", fill = "Healthcare Experience") +
  coord_flip()
```

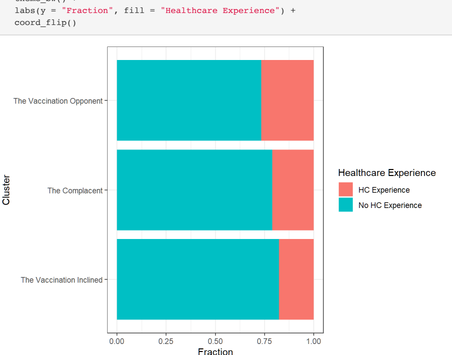

Had COVID-19

```
tib_covid <- tib_hca %>%
  select(Cluster, had_covid) %>%
  drop_na(Cluster) %>%
  drop_na(had_covid) %>%
  mutate(had_covid = dplyr::recode(had_covid,
                                   "1" = "Had COVID",
                                   "2" = "No COVID"))



tib_covid_table <- table(tib_covid$Cluster, tib_covid$had_covid)

chisq_covid <- chisq.test(tib_covid_table, simulate.p.value = TRUE)

chisq_covid
```

```
##
##  Pearson's Chi-squared test with simulated p-value (based on 2000
##  replicates)
##
## data:  tib_covid_table
## X-squared = 6.1267, df = NA, p-value = 0.04948
```

```
chisq.residuals(tib_covid_table, std = FALSE)
```

```
##
##                            Had COVID No COVID
##   The Vaccination Inclined     -1.61     0.45
##   The Complacent                0.55    -0.15
##   The Vaccination Opponent      1.67    -0.47
```

```
chisq.residuals(tib_covid_table, std = TRUE)
```

```
##
##                            Had COVID No COVID
##   The Vaccination Inclined     -2.41     2.41
##   The Complacent                0.62    -0.62
##   The Vaccination Opponent      2.09    -2.09
```

```
covid_contrib <- round(100*chisq_covid$residuals^2/chisq_covid$statistic, 3)

covid_contrib
```

```
##
##                            Had COVID No COVID
##   The Vaccination Inclined    42.204    3.339
##   The Complacent               4.864    0.385
##   The Vaccination Opponent    45.601    3.607
```

```
ggplot(tib_covid, aes(x = Cluster, fill = had_covid)) +
  geom_bar(position = "fill") +
  theme_bw() +
  labs(y = "Fraction", fill = "Had COVID-19") +
  coord_flip()
```

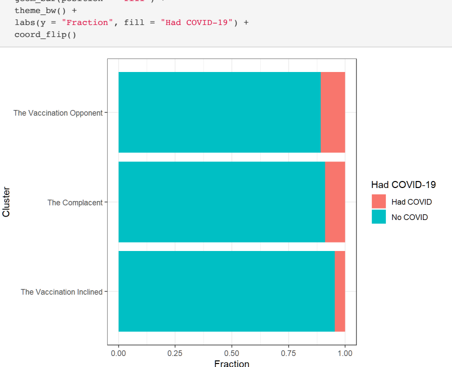

Influenza Vaccination Status

```
tib_ivax_status <- tib_hca %>%
  select(Cluster, i_vaccine_taken) %>%
  drop_na(Cluster) %>%
  drop_na(i_vaccine_taken) %>%
  mutate(ivax_status = dplyr::recode(i_vaccine_taken,
                                     "1" = "Vaccinated",
                                     "2" = "Not Vaccinated"))

tib_ivax_status_table <- table(tib_ivax_status$Cluster, tib_ivax_status$ivax_status)

chisq_ivax_status <- chisq.test(tib_ivax_status_table)

chisq_ivax_status
```

```
##
##  Pearson's Chi-squared test
##
## data:  tib_ivax_status_table
## X-squared = 26.369, df = 2, p-value = 1.879e-06
```

```
chisq.residuals(tib_ivax_status_table, std = FALSE)
```

```
##
##                            Vaccinated Not Vaccinated
##   The Vaccination Inclined       3.36          -1.08
##   The Complacent                -1.51           0.48
##   The Vaccination Opponent      -3.21           1.03
```

```
chisq.residuals(tib_ivax_status_table, std = TRUE)
```

```
##
##                            Vaccinated Not Vaccinated
##   The Vaccination Inclined       5.09          -5.09
##   The Complacent                -1.74           1.74
##   The Vaccination Opponent      -4.07           4.07
```

```
ivax_status_contrib <- round(100*chisq_ivax_status$residuals^2/chisq_ivax_status$statistic, 3)

ivax_status_contrib
```

```
##
##                            Vaccinated Not Vaccinated
##   The Vaccination Inclined     42.918          4.406
##   The Complacent                8.681          0.891
##   The Vaccination Opponent     39.089          4.013
```

```
ggplot(tib_ivax_status, aes(x = Cluster, fill = ivax_status)) +
  geom_bar(position = "fill") +
  theme_bw() +
  labs(y = "Fraction", fill = "Influenza Vaccination Status") +
  coord_flip()
```

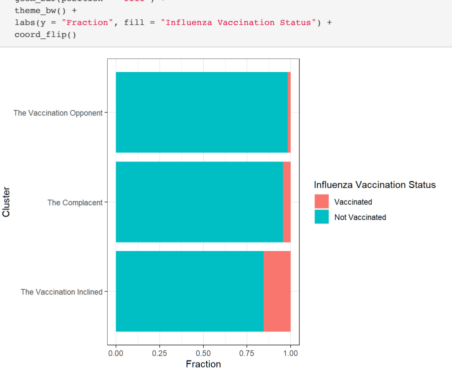

Create a plot for demographics by cluster membership

```
tib_age <- tib_hca %>%
  select(Cluster, age) %>%
  mutate(Cluster = factor(Cluster, c("The Vaccination Inclined",
                                     "The Complacent",
                                     "The Vaccination Opponent")))

age1 <- ggplot(tib_age, aes(x = Cluster, y = age, group = Cluster)) +
  geom_boxplot(fill = "#648499") +
  theme_bw() +
  stat_summary(geom = "crossbar", fun = "mean", color = "red") +
  ylab("Age") +
  theme(legend.position = "none") +
  ggtitle("(A).")

tib_gender <- tib_gender %>%
  mutate(Cluster = factor(Cluster, c("The Vaccination Opponent",
                                     "The Complacent",
                                     "The Vaccination Inclined")))

gender1 <- ggplot(tib_gender, aes(x = Cluster, fill = gender)) +
  geom_bar(position = "fill") +
  theme_bw() +
  labs(y = element_blank(),
       fill = "Gender") +
  scale_y_reverse() +
  coord_flip() +
  scale_fill_manual(values = c("#648499", "#BBBBBB"), breaks = c("Man", "Woman")) +
  theme(legend.position = "right", legend.justification = "left", axis.ticks.x = element_blank(), axis.text.x = e
lement_blank()) +
  ggtitle("(B).")

tib_education <- tib_education %>%
  mutate(Cluster = factor(Cluster, c("The Vaccination Opponent",
                                     "The Complacent",
                                     "The Vaccination Inclined")))

education1 <- ggplot(tib_education, aes(x = Cluster, fill = education)) +
  geom_bar(position = "fill") +
  theme_bw() +
  labs(y = element_blank(), fill = "Level of Education") +
  scale_y_reverse() +
  coord_flip() +
  scale_fill_manual(values = c("#648499", "#BBBBBB")) +
  theme(legend.position = "right", legend.justification = "left", axis.ticks.x = element_blank(), axis.text.x = e
lement_blank()) +
  ggtitle("(C).")

tib_live <- tib_live %>%
  mutate(Cluster = factor(Cluster, c("The Vaccination Opponent",
                                     "The Complacent",
                                     "The Vaccination Inclined")))

home1 <- ggplot(tib_live, aes(x = Cluster, fill = live)) +
  geom_bar(position = "fill") +
  theme_bw() +
  labs(y = element_blank(), fill = "Home Region") +
  scale_y_reverse() +
  coord_flip() +
  scale_fill_manual(values = c("#648499", "#BBBBBB", "#E4E4E4"),
                    labels = c("Uusimaa", "Southwest Finland", "Other")) +
  theme(legend.position = "right", legend.justification = "left", axis.ticks.x = element_blank(), axis.text.x = e
lement_blank()) +
  ggtitle("(D).")

tib_hcw <- tib_hcw %>%
  mutate(Cluster = factor(Cluster, c("The Vaccination Opponent",
                                     "The Complacent",
                                     "The Vaccination Inclined")))

hcw1 <- ggplot(tib_hcw, aes(x = Cluster, fill = hcw)) +
  geom_bar(position = "fill") +
  theme_bw() +
  labs(y = element_blank(), fill = "Healthcare Experience") +
  scale_y_reverse() +
  coord_flip() +
  scale_fill_manual(values = c("#648499", "#BBBBBB")) +
  theme(legend.position = "right", legend.justification = "left", axis.ticks.x = element_blank(), axis.text.x = e
lement_blank()) +
  ggtitle("(E).")

tib_covid <- tib_covid %>%
  mutate(Cluster = factor(Cluster, c("The Vaccination Opponent",
                                     "The Complacent",
                                     "The Vaccination Inclined")))

covid1 <- ggplot(tib_covid, aes(x = Cluster, fill = had_covid)) +
  geom_bar(position = "fill") +
  theme_bw() +
  labs(y = element_blank(), fill = "Had COVID-19") +
  scale_y_reverse() +
  coord_flip() +
  scale_fill_manual(values = c("#648499", "#BBBBBB")) +
  theme(legend.position = "right", legend.justification = "left", axis.ticks.x = element_blank(), axis.text.x = e
lement_blank()) +
  ggtitle("(F).")

tib_ivax_status <- tib_ivax_status %>%
  mutate(Cluster = factor(Cluster, c("The Vaccination Opponent",
                                     "The Complacent",
                                     "The Vaccination Inclined")))
ivax1 <- ggplot(tib_ivax_status, aes(x = Cluster, fill = ivax_status)) +
  geom_bar(position = "fill") +
  theme_bw() +
  labs(y = element_blank(), fill = "Influenza Vaccination Status") +
  scale_y_reverse() +
  coord_flip() +
  scale_fill_manual(values = c("#648499", "#BBBBBB")) +
  theme(legend.position = "right", legend.justification = "left", axis.ticks.x = element_blank(), axis.text.x = e
lement_blank()) +
  ggtitle("(F).")

(age1) / (gender1 | education1) / (home1 | hcw1) / (ivax1 | plot_spacer())
```

```
## Warning: Removed 66 rows containing non-finite values (`stat_boxplot()`).
```

```
## Warning: Removed 66 rows containing non-finite values (`stat_summary()`).
```

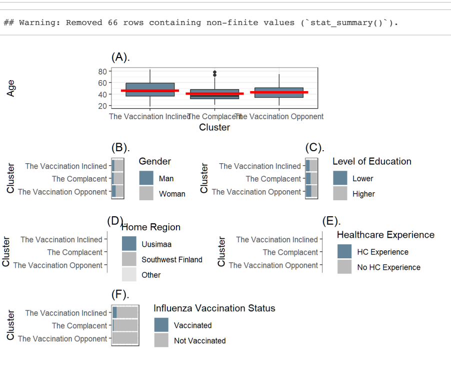

Descriptives

Means and SDs for unstandardized scores

```
tib_means <- tib_hca %>%
  group_by(Cluster) %>%
  get_summary_stats(c(i_vaccine_probability_pre,
                      format_preference,
                      conspiracy_mentality,
                      i_altruism_pre,
                      i_threat_pre,
                      i_vaccine_efficacy_pre,
                      i_vaccine_safe_pre,
                      i_vaccine_easy_pre,
                      trust_vaccines,
                      trust_hca),
                    type = "mean_sd") %>%
  ungroup() %>%
  mutate(mean = round(mean, digits = 2),
         sd = round(sd, digits = 2))

kable(tib_means, "simple")
```

Cluster	variable	n	mean	sd
The Vaccination Inclined	i_vaccine_probability_pre	279	38.60	27.55
The Vaccination Inclined	format_preference	279	-0.26	0.37
The Vaccination Inclined	conspiracy_mentality	279	-0.43	0.48
The Vaccination Inclined	i_altruism_pre	279	62.48	22.83
The Vaccination Inclined	i_threat_pre	279	34.14	21.95
The Vaccination Inclined	i_vaccine_efficacy_pre	279	70.25	20.09
The Vaccination Inclined	i_vaccine_safe_pre	279	85.51	16.43
The Vaccination Inclined	i_vaccine_easy_pre	279	78.82	20.77
The Vaccination Inclined	trust_vaccines	279	5.62	1.22
The Vaccination Inclined	trust_hca	279	0.61	0.48
The Complacent	i_vaccine_probability_pre	90	4.89	8.57
The Complacent	format_preference	90	0.13	0.36
The Complacent	conspiracy_mentality	90	0.11	0.34
The Complacent	i_altruism_pre	90	26.30	20.68
The Complacent	i_threat_pre	90	20.20	20.52
The Complacent	i_vaccine_efficacy_pre	90	50.67	23.10
The Complacent	i_vaccine_safe_pre	90	71.67	19.21
The Complacent	i_vaccine_easy_pre	90	85.09	16.77
The Complacent	trust_vaccines	90	2.59	1.48
The Complacent	trust_hca	90	-0.24	0.39
The Vaccination Opponent	i_vaccine_probability_pre	170	0.68	2.77
The Vaccination Opponent	format_preference	170	0.37	0.38
The Vaccination Opponent	conspiracy_mentality	170	0.68	0.51
The Vaccination Opponent	i_altruism_pre	170	3.94	8.08
The Vaccination Opponent	i_threat_pre	170	8.15	14.33
The Vaccination Opponent	i_vaccine_efficacy_pre	170	14.58	14.95
The Vaccination Opponent	i_vaccine_safe_pre	170	25.25	24.08
The Vaccination Opponent	i_vaccine_easy_pre	170	85.64	20.67
The Vaccination Opponent	trust_vaccines	170	1.22	0.64
The Vaccination Opponent	trust_hca	170	-0.91	0.41

Means and SDs for standardized scores

```
tib_z_means <- tib_hca %>%
  mutate(i_vaccine_probability_pre_z = as.numeric(i_vaccine_probability_pre_z),
         format_preference_z = as.numeric(format_preference_z),
         conspiracy_mentality_z = as.numeric(conspiracy_mentality_z),
         i_altruism_pre_z = as.numeric(i_altruism_pre_z),
         i_threat_pre_z = as.numeric(i_threat_pre_z),
         i_vaccine_efficacy_pre_z = as.numeric(i_vaccine_efficacy_pre_z),
         i_vaccine_safe_pre_z = as.numeric(i_vaccine_safe_pre_z),
         i_vaccine_easy_pre_z = as.numeric(i_vaccine_easy_pre_z),
         trust_vaccines_z = as.numeric(trust_vaccines_z),
         trust_hca_z = as.numeric(trust_hca_z)) %>%
  group_by(Cluster) %>%
  get_summary_stats(c(i_vaccine_probability_pre_z,
                      format_preference_z,
                      conspiracy_mentality_z,
                      i_altruism_pre_z,
                      i_threat_pre_z,
                      i_vaccine_efficacy_pre_z,
                      i_vaccine_safe_pre_z,
                      i_vaccine_easy_pre_z,
                      trust_vaccines_z,
                      trust_hca_z),
                    type = "mean_sd") %>%
  ungroup() %>%
  mutate(mean = round(mean, digits = 2),
         sd = round(sd, digits = 2))

kable(tib_z_means, "simple")
```

Cluster	variable	n	mean	sd
The Vaccination Inclined	i_vaccine_probability_pre_z	279	0.65	1.01
The Vaccination Inclined	format_preference_z	279	-0.56	0.80
The Vaccination Inclined	conspiracy_mentality_z	279	-0.65	0.71
The Vaccination Inclined	i_altruism_pre_z	279	0.75	0.70
The Vaccination Inclined	i_threat_pre_z	279	0.46	0.96
The Vaccination Inclined	i_vaccine_efficacy_pre_z	279	0.67	0.64
The Vaccination Inclined	i_vaccine_safe_pre_z	279	0.64	0.49
The Vaccination Inclined	i_vaccine_easy_pre_z	279	-0.16	1.02
The Vaccination Inclined	trust_vaccines_z	279	0.82	0.53
The Vaccination Inclined	trust_hca_z	279	0.77	0.58
The Complacent	i_vaccine_probability_pre_z	90	-0.59	0.32
The Complacent	format_preference_z	90	0.28	0.77
The Complacent	conspiracy_mentality_z	90	0.15	0.50
The Complacent	i_altruism_pre_z	90	-0.36	0.64
The Complacent	i_threat_pre_z	90	-0.15	0.90
The Complacent	i_vaccine_efficacy_pre_z	90	0.04	0.74
The Complacent	i_vaccine_safe_pre_z	90	0.22	0.58
The Complacent	i_vaccine_easy_pre_z	90	0.15	0.82
The Complacent	trust_vaccines_z	90	-0.49	0.64
The Complacent	trust_hca_z	90	-0.28	0.48
The Vaccination Opponent	i_vaccine_probability_pre_z	170	-0.75	0.10
The Vaccination Opponent	format_preference_z	170	0.78	0.81
The Vaccination Opponent	conspiracy_mentality_z	170	0.98	0.75
The Vaccination Opponent	i_altruism_pre_z	170	-1.04	0.25
The Vaccination Opponent	i_threat_pre_z	170	-0.68	0.63
The Vaccination Opponent	i_vaccine_efficacy_pre_z	170	-1.11	0.48
The Vaccination Opponent	i_vaccine_safe_pre_z	170	-1.17	0.72
The Vaccination Opponent	i_vaccine_easy_pre_z	170	0.18	1.01
The Vaccination Opponent	trust_vaccines_z	170	-1.09	0.28
The Vaccination Opponent	trust_hca_z	170	-1.11	0.50

Demographic variable counts and frequencies by cluster

```
tib_n <- tib_hca %>%
  select(Cluster,
         age,
         sex,
         education,
         live,
         hcw,
         i_vaccine_taken,
         i_vaccination_experience) %>%
  mutate(age = case_when(age < 30 ~ "1829",
                         age < 40 ~ "3039",
                         age < 50 ~ "4049",
                         age < 60 ~ "5059",
                         age < 70 ~ "6069",
                         age < 80 ~ "7079",
                         age < 90 ~ "8089",
                         age < 100 ~ "9099",
                         age >= 100 ~ "100"),
         sex = recode_factor(sex,
                             "1" = "Male",
                             "2" = "Female",
                             "3" = "Other",
                             "4" = "DWS"),
         education = recode_factor(education,
                                   "1" = "Lower",
                                   "2" = "Lower",
                                   "3" = "Lower",
                                   "4" = "Lower",
                                   "5" = "Lower",
```

```
                                        "6" = "Lower",
                                        "7" = "Higher",
                                        "8" = "Higher",
                                        "9" = "Higher",
                                        "10" = "Other"),
         live = recode_factor(live,
                              "1" = "Uusimaa",
                              "2" = "Southwest",
                              "3" = "Other",
                              "4" = "Other",
                              "5" = "Other",
                              "6" = "Other",
                              "7" = "Other",
                              "8" = "Other",
                              "9" = "Other",
                              "10" = "Other",
                              "11" = "Other",
                              "12" = "Other",
                              "13" = "Other",
                              "14" = "Other",
                              "15" = "Other",
                              "16" = "Other",
                              "17" = "Other",
                              "18" = "Other",
                              "19" = "Other",
                              "20" = "Other"),
         hcw = recode_factor(hcw,
                             "1" = "Yes",
                             "2" = "No"),
         i_vaccine_taken = recode_factor(i_vaccine_taken,
                                         "1" = "Yes",
                                         "2" = "No"),
         i_vaccination_experience = recode_factor(i_vaccination_experience,
                                                  "1" = "VeryNegative",
                                                  "2" = "SomeNegative",
                                                  "3" = "Neutral",
                                                  "4" = "SomePositive",
                                                  "5" = "VeryPositive")) %>%
  pivot_longer(cols = c(age,
                        sex,
                        education,
                        live,
                        hcw,
                        i_vaccine_taken,
                        i_vaccination_experience),
               names_to = "variable",
               values_to = "value")
tib_n %>%
  group_by(Cluster,
           variable,
           value) %>%
  summarise(n = n()) %>%
  mutate(freq = (n / sum(n)) * 100,
         freq = round(freq, digits = 1)) %>%
  kable("simple")
```

```
## `summarise()` has grouped output by 'Cluster', 'variable'. You can override
## using the `.groups` argument.
```

Cluster	variable	value	n	freq
The Vaccination Inclined	age	1829	33	11.8
The Vaccination Inclined	age	3039	61	21.9
The Vaccination Inclined	age	4049	59	21.1
The Vaccination Inclined	age	5059	39	14.0
The Vaccination Inclined	age	6069	48	17.2
The Vaccination Inclined	age	7079	12	4.3
The Vaccination Inclined	age	8089	1	0.4
The Vaccination Inclined	age	NA	26	9.3
The Vaccination Inclined	education	Higher	183	65.6
The Vaccination Inclined	education	Lower	92	33.0
The Vaccination Inclined	education	Other	4	1.4
The Vaccination Inclined	hcw	No	229	82.1
The Vaccination Inclined	hcw	Yes	49	17.6
The Vaccination Inclined	hcw	NA	1	0.4
The Vaccination Inclined	i_vaccination_experience	Neutral	25	9.0
The Vaccination Inclined	i_vaccination_experience	SomeNegative	1	0.4
The Vaccination Inclined	i_vaccination_experience	SomePositive	7	2.5
The Vaccination Inclined	i_vaccination_experience	VeryPositive	10	3.6
The Vaccination Inclined	i_vaccination_experience	NA	236	84.6
The Vaccination Inclined	i_vaccine_taken	No	235	84.2
The Vaccination Inclined	i_vaccine_taken	Yes	43	15.4
The Vaccination Inclined	i_vaccine_taken	NA	1	0.4
The Vaccination Inclined	live	Other	131	47.0
The Vaccination Inclined	live	Southwest	44	15.8
The Vaccination Inclined	live	Uusimaa	104	37.3
The Vaccination Inclined	sex	DWS	3	1.1
The Vaccination Inclined	sex	Female	214	76.7
The Vaccination Inclined	sex	Male	55	19.7
The Vaccination Inclined	sex	Other	5	1.8
The Vaccination Inclined	sex	NA	2	0.7
The Complacent	age	1829	17	18.9
The Complacent	age	3039	25	27.8
The Complacent	age	4049	18	20.0
The Complacent	age	5059	9	10.0
The Complacent	age	6069	8	8.9
The Complacent	age	7079	2	2.2
The Complacent	age	NA	11	12.2
The Complacent	education	Higher	51	56.7
The Complacent	education	Lower	36	40.0
The Complacent	education	Other	3	3.3
The Complacent	hcw	No	71	78.9
The Complacent	hcw	Yes	19	21.1
The Complacent	i_vaccination_experience	Neutral	3	3.3
The Complacent	i_vaccination_experience	SomeNegative	1	1.1
The Complacent	i_vaccination_experience	NA	86	95.6
The Complacent	i_vaccine_taken	No	86	95.6
The Complacent	i_vaccine_taken	Yes	4	4.4
The Complacent	live	Other	41	45.6
The Complacent	live	Southwest	20	22.2
The Complacent	live	Uusimaa	29	32.2
The Complacent	sex	DWS	5	5.6
The Complacent	sex	Female	72	80.0
The Complacent	sex	Male	12	13.3
The Complacent	sex	Other	1	1.1
The Vaccination Opponent	age	1829	19	11.2
The Vaccination Opponent	age	3039	40	23.5
The Vaccination Opponent	age	4049	38	22.4
The Vaccination Opponent	age	5059	25	14.7
The Vaccination Opponent	age	6069	17	10.0
The Vaccination Opponent	age	7079	2	1.2
The Vaccination Opponent	age	NA	29	17.1
The Vaccination Opponent	education	Higher	86	50.6
The Vaccination Opponent	education	Lower	76	44.7
The Vaccination Opponent	education	Other	7	4.1
The Vaccination Opponent	education	NA	1	0.6
The Vaccination Opponent	hcw	No	124	72.9
The Vaccination Opponent	hcw	Yes	45	26.5
The Vaccination Opponent	hcw	NA	1	0.6
The Vaccination Opponent	i_vaccination_experience	SomeNegative	2	1.2
The Vaccination Opponent	i_vaccination_experience	VeryNegative	1	0.6
The Vaccination Opponent	i_vaccination_experience	NA	167	98.2
The Vaccination Opponent	i_vaccine_taken	No	166	97.6
The Vaccination Opponent	i_vaccine_taken	Yes	3	1.8
The Vaccination Opponent	i_vaccine_taken	NA	1	0.6
The Vaccination Opponent	live	Other	101	59.4
The Vaccination Opponent	live	Southwest	14	8.2
The Vaccination Opponent	live	Uusimaa	51	30.0
The Vaccination Opponent	live	NA	4	2.4
The Vaccination Opponent	sex	DWS	11	6.5
The Vaccination Opponent	sex	Female	105	61.8
The Vaccination Opponent	sex	Male	51	30.0
The Vaccination Opponent	sex	Other	1	0.6
The Vaccination Opponent	sex	NA	2	1.2

Demographic variable counts and frequencies for whole sample

```
tib_n %>%
  group_by(variable,
           value) %>%
  summarise(n = n()) %>%
  mutate(freq = (n / sum(n)) * 100,
         freq = round(freq, digits = 1)) %>%
  kable("simple")
```

```
## `summarise()` has grouped output by 'variable'. You can override using the
## `.groups` argument.
```

variable	value	n	freq
age	1829	69	12.8
age	3039	126	23.4
age	4049	115	21.3
age	5059	73	13.5
age	6069	73	13.5
age	7079	16	3.0
age	8089	1	0.2
age	NA	66	12.2
education	Higher	320	59.4
education	Lower	204	37.8
education	Other	14	2.6
education	NA	1	0.2
hcw	No	424	78.7
hcw	Yes	113	21.0
hcw	NA	2	0.4
i_vaccination_experience	Neutral	28	5.2
i_vaccination_experience	SomeNegative	4	0.7
i_vaccination_experience	SomePositive	7	1.3
i_vaccination_experience	VeryNegative	1	0.2
i_vaccination_experience	VeryPositive	10	1.9
i_vaccination_experience	NA	489	90.7
i_vaccine_taken	No	487	90.4
i_vaccine_taken	Yes	50	9.3
i_vaccine_taken	NA	2	0.4
live	Other	273	50.6
live	Southwest	78	14.5
live	Uusimaa	184	34.1
live	NA	4	0.7
sex	DWS	19	3.5
sex	Female	391	72.5
sex	Male	118	21.9
sex	Other	7	1.3
sex	NA	4	0.7
